# Protected and De-protected Platinum(IV) Glycoconjugates With GLUT1 and OCT2-Mediated Selective Cancer Targeting: Demonstrated Enhanced Transporter-Mediated Cytotoxic Properties *in vitro* and *in vivo*

**DOI:** 10.3389/fchem.2018.00386

**Published:** 2018-09-21

**Authors:** Jing Ma, Hanfang Liu, Zhuoqing Xi, Jiuzhou Hou, Yingguang Li, Jie Niu, Tong Liu, Shuning Bi, Xin Wang, Chaojie Wang, Jiajia Wang, Songqiang Xie, Peng G. Wang

**Affiliations:** ^1^Institute of Chemical Biology, College of Pharmacy, Henan University, Kaifeng, China; ^2^Henan University Joint National Laboratory for Antibody Drug Engineering, School of Basic Medicine Science, Henan University, Kaifeng, China; ^3^The Key Laboratory of Natural Medicine and Immuno-Engineering, Henan University, Kaifeng, China; ^4^Tianjin Key Laboratory of Molecular Drug Research, State Key Laboratory of Elemento-organic Chemistry, College of Pharmacy, Nankai University, Tianjin, China

**Keywords:** platinum(IV) glycoconjugates, glucose transporter 1, organic cation transporter 2, cancer targeting, transporter-mediated cytotoxic properties

## Abstract

Physiological characteristics of human malignancies are increased glycolysis and overexpression of glucose transporters (GLUTs). ^18^Flurodeoxyglucose-positron emission tomography (FDG-PET) has successfully developed as clinical modality for the diagnosis and staging of many cancers based on the Warburg effect. To leverage this glucose transporter mediated metabolic disparity between normal and malignant cells, in the current report, protected, and de-protected glucose, mannose, galactose, rhamnose, maltose, and lactose-conjugated platinum(IV) complexes were designed and synthesized. The suggested potential of facilitated intravenous to oral switching of glycosylated platinum(IV) prodrugs with cancer-targeting properties were evaluated for glucose transporter 1 (GLUT1) and organic cation transporter 2 (OCT2)-mediated selective properties *in vitro* and *in vivo*. The cytotoxicity of **2d**, **5d**, and **6d** were ~23-fold greater than that of the positive controls cisplatin, oxaliplatin, and satraplatin, respectively. The leading compound **6d**, the IC_50_ of which with the GLUT1 inhibitor 4,6-oethylidene-α-D-glucose (EDG) and phloretin (31.80 and 38.71 μM) are 36- and 44-folds higher, respectively, than the 48 h IC_50_ (0.89 μM), is superior to the reported **5**-**8**, exhibiting enhanced cancer targeting. The compounds also showed reduced toxicity to normal cells (293T IC_50_ = 12.06 μM and 3T3 cells IC_50_ > 100 μM) and exhibited no cross-resistance to cisplatin. Moreover, the encouraging selectivity of **6d** for MCF-7 cells *in vivo* indicated that the pyranoside performs an important function in cancer targeting.

## Introduction

Platinum(IV)-based anticancer prodrugs can overcome the limitations of platinum(II) drugs such as cisplatin, carboplatin, and oxaliplatin, which are widely used clinically (Wang and Lippard, 2005; Kelland, 2007; Wheate et al., 2010) Recently, platinum(IV) prodrugs as candidates have been designed, synthesized and evaluated as antitumor agents (Wang et al., 2015; Johnstone et al., 2016). Most platinum(IV) drugs with dual axial ligands offer the opportunity to append additional functional groups for pro-drug activation, tumor targeting, or drug delivery (Basu et al., 2016). Compared with platinum(II) complexes, platinum(IV) complexes not only exhibit increased stability and reduced side effects but also facilitate the intravenous-to-oral switch in cancer chemotherapy (Han et al., 2015). Recent advances in platinum(IV) complexes, in combination with tumor targeting and drug delivery, have been highlighted, addressing the shortcomings of platinum(II) and platinum(IV) complexes, such as their instability in blood, irreversible binding to plasma proteins and nonspecific distribution, as well as the passive and active targeting effects to improve platinum(II) anticancer therapy (Cheff and Hall, 2017). Liposomes (Liu D. et al., 2013), polymers (Haxton and Burt, 2009), inorganic nanomaterials (Min et al., 2010, 2012; Wong et al., 2013), metal–organic frameworks (Oberoi et al., 2013) and calcium phosphate (CaP) nanoparticles (Shi et al., 2015) have also been used to design of platinum(IV) prodrugs for drug delivery. The tumor selective action and attenuated systemic toxicity of the drugs can be enhanced by functionalization of axial ligands. Satraplatin, the best potential platinum(IV) drug for oral administration, has entered phase III clinical trials. However, to the best of our knowledge, satraplatin enters cells without cancer-targeting properties.

**Figure 1:**
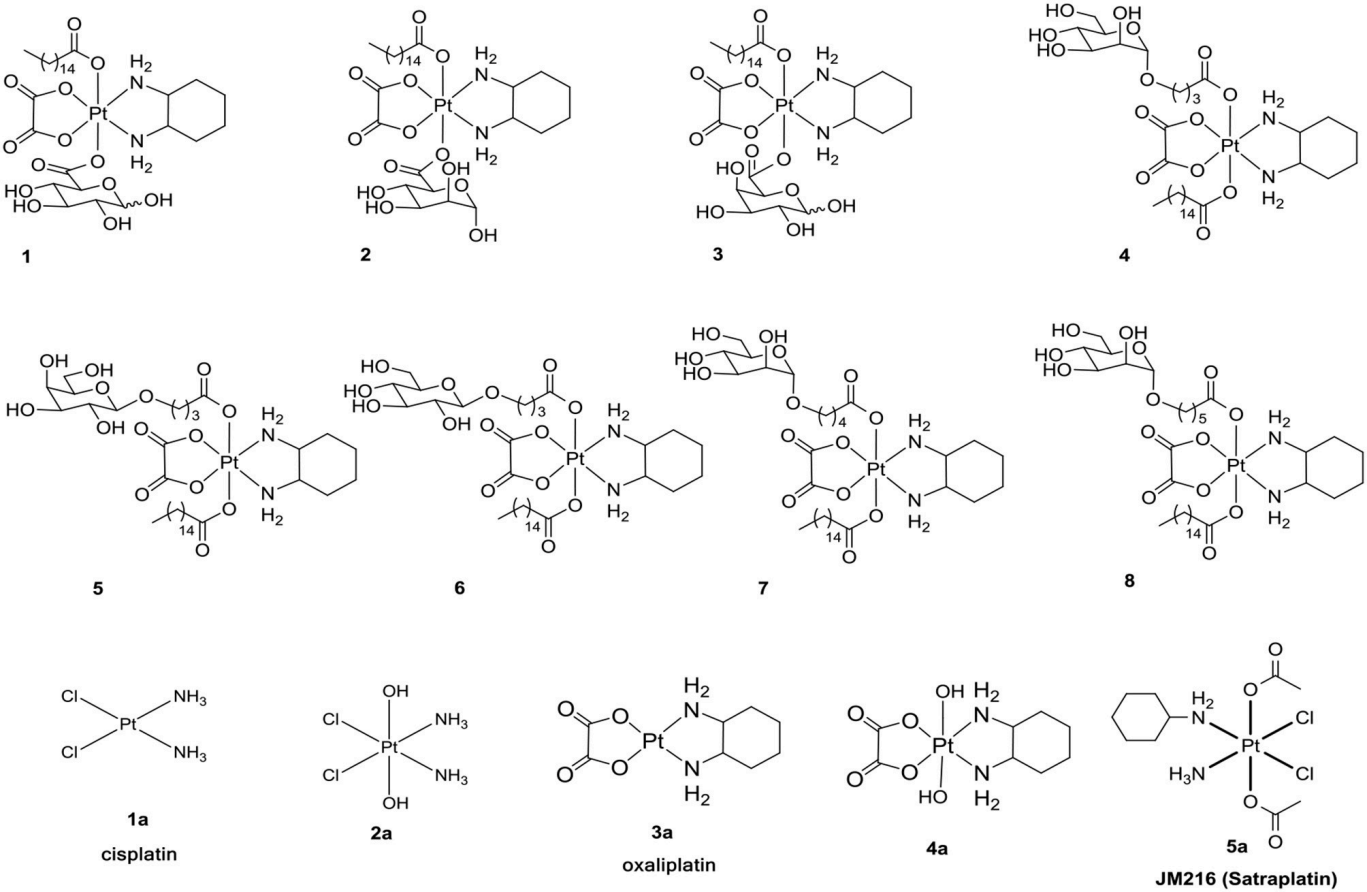
Structures of **1**-**8** and **1a**-**5a** as positive controls.

Increased understanding of this dysfunctional metabolism, known as the Warburg effect, has led to an interest in cancer therapy. One promising strategy for such targeting is glycoconjugation, in which incorporated glucose and other monosaccharides of the drug can efficiently improve cancer targeting and selectivity (Ben-Haim and Ell, 2009; Calvaresi and Hergenrother, 2013; He et al., 2016). The glycoconjugation of platinum(II) was developed to enhance targeted therapy and to decrease toxicity (Chen et al., 1999; Hartinger et al., 2008; Möker and Thiem, 2012; Liu P. et al., 2013; Li et al., 2015; Patra et al., 2016a,b). Due to the special inertia of platinum(IV) complexes, unwanted side reactions with biological nucleophiles are resisted to reduce undesired side effects and to increase the lifetime in biological fluids. We have reported the glycoconjugation of platinum(II) and platinum(IV) prodrugs (Chen et al., 1999; Ma et al., 2016, 2017a,b; Wang et al., 2016). However, in the following experiments, we found that there is a slight dependency between the transporters and the reported compounds **1-8** (Figure 1) (Ma et al., 2017a).

Based on the previous results regarding the design the glycosylated drugs (Calvaresi and Hergenrother, 2013), we deduced that steric hindrance of hexadecanoic acid blocked the combination of the reported compounds (**1**-**8**) with transporters. In the following design of platinum(IV) pro-drugs, we found that the platinum(IV) complexes platin-A with mono-functionalized ligands of aspirin were reported to have a positive shift of 42 mV at a pH of 6.4 and a negative shift of−563 mV at a pH of 7.4 (Warburg, 1956; Vander Heiden et al., 2009; Szablewski, 2013; Cheng et al., 2014; Pathak et al., 2014). The reduction properties indicated that a lower pH in the cancer microenvironment would facilitate reduction of platinum(IV) complexes with mono-functionalized ligands to release active platinum(II) complexes. Moreover, mono-functionalized platinum(IV) complexes exhibited low toxicity to normal cells. The use of the monohydroxido monocarboxylato framework was also reported due to its intermediate properties in the design of targeted platinum(IV) derivatives of oxaliplatin, with reasonable rates of reduction compared with either the dihydroxido or the dicarboxylato framework (Zhang et al., 2012).

**Scheme 1:**
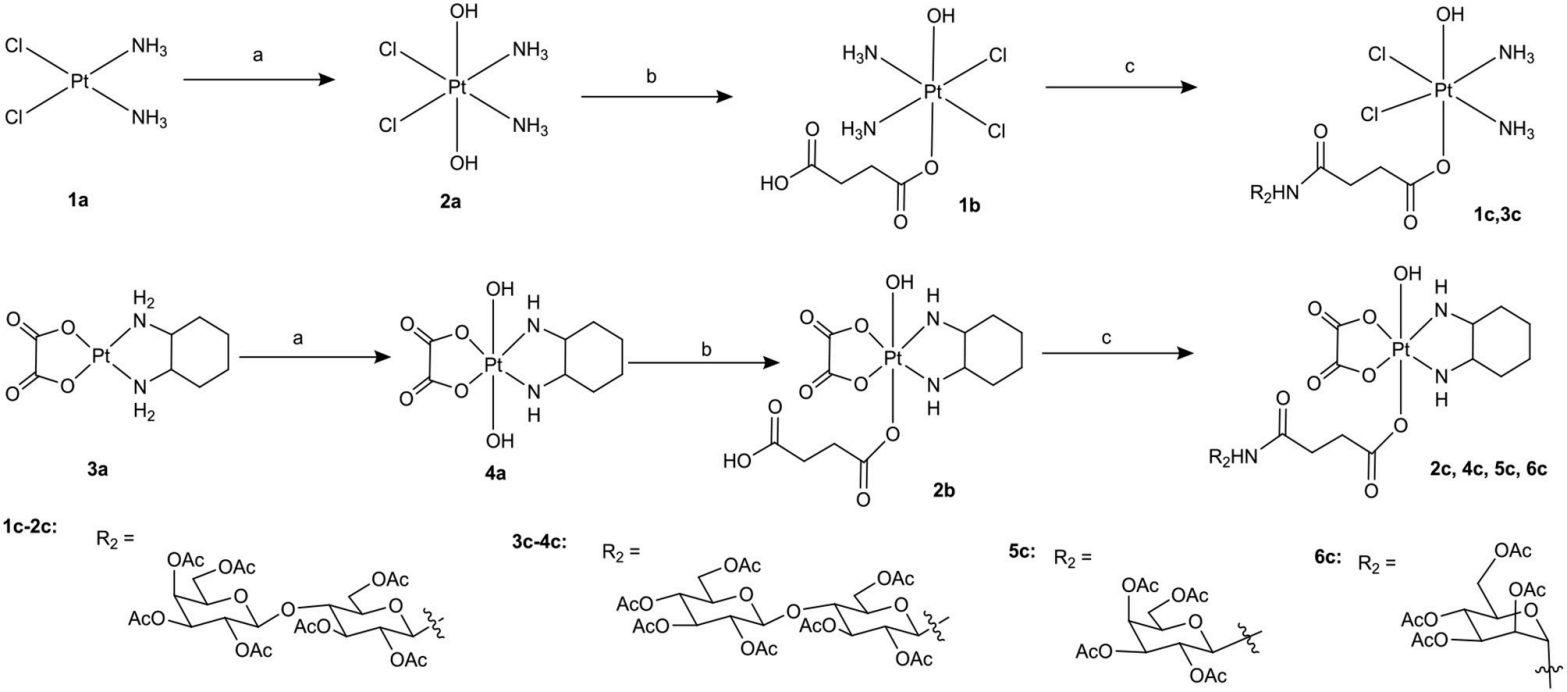
Synthetic route of target compounds **1c**-**6c** and **5a**. Conditions and reagents: (a) H_2_O_2_ (30% w/v)/H_2_O; (b) Succinic anhydride, DMSO; (c) HATU, DIPEA, and DMF, r.t.

**Scheme 2:**
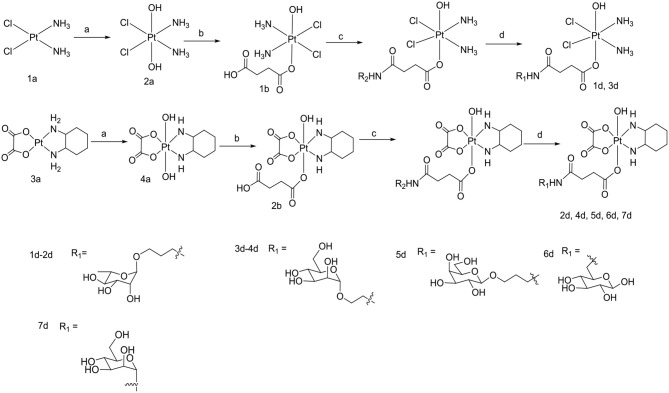
Synthetic routes of target compounds **1d**-**7d**. Conditions and reagents: (a) H_2_O_2_ (30% w/v)/H_2_O; (b) Succinic anhydride, DMSO; (c) HATU, DIPEA, and DMF, r.t.; (d) BCl_3_ and DCM, 78°C.

Inspired by these observations and as an extension of our study, it was of great interest for us to design and synthesize a series of mono-functionalized glycosylated platinum(IV) analogs **1c**-**6c** and **1d**-**7d** (Scheme 1, 2) and to evaluate their bioactivities *in vitro* and *in vivo*. Herein, we disclose the synthesis of the mono-functionalized platinum(IV) prodrugs cisplatin and oxaliplatin as core, glucose, mannose, rhamnose, and galactose abundant in different cancer cells as monosaccharides and lactose and maltose as disaccharides, which are also overexpressed in cancer cells. The crystal structure of human GLUT1 was also recently published (Sun et al., 2012; Deng et al., 2014). This structure reveals that all of the hydroxy groups of D-glucose except that on C6 are involved in hydrogen-bonding interactions with various amino acid residues of the transporter. Modification at the C6 position of D-glucose should not, therefore, interfere with receptor binding. Previous reports have also suggested that the C6 position of D-glucose can tolerate various functional groups while retaining substrate specificity for, and internalization by, GLUT1 (Gynther et al., 2009; Kumar et al., 2012). In fact, C6-glucose conjugates of 4-nitrobenzofurazan, ketoprofen, and indomethacin have been reported to bind to GLUT1 with even higher affinity than unmodified D-glucose (Barros et al., 2009; Gynther et al., 2009; Kumar et al., 2012). Based on these observations, the C6-modified Pt(IV) complex **6d** was designed to bind with GLUT efficiently. Currently, functionalization is most commonly conducted using anhydrides and acyl chlorides, which undergo direct carboxylation reactions with platinum(IV) axial hydroxido ligands (Kelland et al., 1992). Therefore, succinic anhydride was introduced to the platinum(IV) axial hydroxido ligands.

## Results

### Synthesis of target compounds

To the best of our knowledge, the synthesis routes of **1c**-**6c** and **1d**-**7d** provided in detail in the supporting information have not been reported. Hydrogen peroxide oxidation of cisplatin (**1a**) and oxaliplatin (**3a**) in water affords **2a** and **4a**. In the reaction of platinum(IV) derivatives **2a** or **4a** with succinic anhydride with the molar ratio of 1:1 at room temperature in DMSO (10 mL/0.2 g Pt), only monosubstituted derivatives **1b** or **2b** were obtained. **1c**-**6c** (Scheme 1) can be obtained from the combination of various per-*O*-acetylated 1-aminosugars and the carboxylic function of the platinum(IV) complexes. Further, derivatives **1d**-**7d** (Scheme 2) can be obtained in the reaction between per-*O*-benzylated aminoalkyl *O*-glycosides and platinum(IV) complexes with carboxylic function in the succinic linker. The benzyl protecting groups can be removed in the presence of anhydrous DCM, BCl_3_ (1 M) at −78° for 2 h. The pure desired products can be obtained after purification by column chromatography.

The platinum(IV) compounds **1c**-**6c** and **1d**-**7d** were fully characterized by (^1^H and ^13^C) NMR spectroscopy and electrospray ionization mass spectrometry (ESI-MS) (in the Supplementary Material). The purity of the platinum complexes was confirmed to be ≥95% by analytical HPLC.

#### Antitumor activities *in vitro*

We evaluated the cytotoxicity of 1c-6c, 1d-7d and of the positive control 1a-5a against a panel of human cancer cells using MTT (3-(4,5-dimethylthiazol-2-yl)-2,5-diphenyltetrazolium bromide) assay. The inhibitory effects (IC_50_ in μM) of carbohydrate platinum(IV) complexes on cancer cells are summarized in Table 1. The IC_50_ values were calculated based on three parallel experiments.

**Table 1:** Cytotoxicity profiles of glycosylated Pt(IV) complexes (IC_50_ in μM) in seven human carcinoma cell lines.

	**HeLa**	**MCF-7**	**LNCaP**	**PC3**	**HepG-2**	**A549**	**A549R**	**RF^a^**
1a	0.80 ± 0.93	2.28 ± 0.56	23.83 ± 0.89	22.85 ± 1.45	3.94 ± 0.39	10.85 ± 1.98	35.78 ± 1.69	3.3
2a	11.36 ± 1.56	27.01 ± 0.34	50.78 ± 0.79	61.42 ± 0.67	50.00 ± 1.79	>100	13.40 ± 2.57	/
3a	3.96 ± 0.75	5.22 ± 2.78	16.78 ± 0.34	39.74 ± 0.76	6.64 ± 1.79	10.06 ± 1.20	39.67 ± 0.69	3.94
4a	15.78 ± 0.34	>100	22.97 ± 0.89	>100	>100	>100	29.67 ± 0.64	/
5a	3.10 ± 0.89	8.01 ± 0.56	5.23 ± 1.77	16.77 ± 1.56	6.70 ± 1.89	2.17 ± 1.68	9.96 ± 0.57	4.59
1c	18.21 ± 1.01	24.18 ± 1.44	>100	>100	11.62 ± 1.58	36.38 ± 1.39	4.26 ± 1.90	0.12
2c	4.05 ± 0.87	8.29 ± 1.87	4.70 ± 0.89	>100	59.34 ± 1.49	14.54 ± 1.47	8.37 ± 1.90	0.58
3c	0.91 ± 0.54	27.22 ± 1.56	>100	1.13 ± 0.45	3.27 ± 1.67	3.38 ± 1.49	14.64 ± 1.78	4.33
4c	>100	7.21 ± 0.78	>100	22.88 ± 0.78	10.67 ± 1.49	14.33 ± 1.48	25.20 ± 1.79	1.79
5c	32.21 ± 0.34	17.89 ± 0.12	22.12 ± 1.45	7.45 ± 0.56	>100	17.48 ± 0.89	74.08 ± 1.67	4.24
6c	16.77 ± 0.45	20.76 ± 0.45	20.10 ± 1.78	7.06 ± 0.56	50.92 ± 1.46	23.81 ± 0.78	>100	/
1d	3.10 ± 0.78	39.64 ± 0.79	/	14.33 ± 0.56	14.09 ± 1.46	10.56 ± 2.31	10.04 ± 1.69	0.95
2d	0.66 ± 0.65	0.61 ± 0.66	10.89 ± 0.89	7.86 ± 0.56	7.90 ± 1.46	0.89 ± 2.98	1.94 ± 1.69	2.18
3d	3.01 ± 0.67	45.06 ± 0.77	>100	>100	13.18 ± 1.38	1.90 ± 2.78	>100	/
4d	2.63 ± 0.78	15.55 ± 0.56	>100	>100	17.16 ± 1.46	1.94 ± 1.78	>100	/
5d	0.58 ± 0.78	0.35 ± 1.34	12.04 ± 0.79	6.27 ± 1.45	1.77 ± 1.39	2.29 ± 1.78	5.67 ± 1.69	2.48
6d	2.88 ± 0.67	0.89 ± 1.34	20.01 ± 0.79	66.62 ± 1.45	4.10 ± 1.46	29.88 ± 1.78	47.00 ± 1.69	1.57
7d	3.01 ± 0.67	1.36 ± 1.34	20.78 ± 0.79	>100	4.30 ± 1.46	36.46 ± 1.78	42.00 ± 1.69	1.15

a *RF: Resistance factor = IC_50_(A549R)/IC_50_(A549)*.

The cytotoxicity of compounds 1c-6c and 1d-7d shows a rather broad antitumor spectrum and is greater than that of the positive controls 1a, 3a, and 5a. Moreover, compared with the oxoplatins 2a and 4a, the introduction of the de-protected monosaccharide and protected disaccharide to the platinum(IV) complexes dramatically enhances the activity, and different glycosyl fragments play important roles in the antitumor activity. In addition, as we all know, tumor-specific and transporter-targeted sugar conjugates possess promising features that are desired in antitumor agents for targeted therapy drugs, while the carbohydrate platinum(IV) complexes with different cores, such as cisplatin and oxaliplatin, also display different antitumor efficacy. In addition, the glycosylated platinum(IV) compounds can overcome the drug resistance of A549R cells (RF 0.12–4.33), especially compounds 1c and 2c with RF 0.12 and 0.58, respectively.

As shown in Table 1, for HeLa, MCF-7, and HepG-2 cells, the cytotoxicity of 7d is 5.57, 15.27- and 11.8-fold higher, respectively, than 6c, the structure of which is similar to 7d except for protection by acetyl. Additionally, for A549R, the cytotoxicity of 7d is higher than that of 6c (IC_50_ > 100 μM). The results indicate that the de-protected glycosylated platinum(IV) complex 7d is superior to the protected complex 6c, which might contribute the different trans-membrane mechanism.

Of the per-*O*-acetylated glycosylated platinum(IV) derivatives 1c-6c, compounds 1c-4c equipped with disaccharide effectively inhibit the growth of the tested cell lines, especially HeLa, MCF-7, HepG-2, and A549R. Of the de-protected glycosylated platinum(IV) derivatives 1d-7d, rhamnoside (2d), galactoside (5d), and glucoside (6d) glycosylated platinum(IV) derivatives with oxaliplatin core show particularly greater antitumor activity, which is 0.5- to 23-fold higher than 1a, 3a, and 5a for HeLa, MCF-7, A549, A549R, and HepG-2 cells, respectively. Moreover, as for A549R, LnCaP, and PC3, cisplatin and oxaliplatin have been reported to have low anti-tumor effects with IC_50_ values of 16.78–39.74 μM. However, the IC_50_ values of 2d and 5d (1.94–12.01 μM) indicated potential effects clinically. Additionally, glucose platinum(IV) prodrugs (6d) show decreased toxicity to normal cells and increased toxicity to cancer cells, especially to HeLa, MCF-7, HepG-2, and A549. These properties suggest that the compound shows great potential for further investigation.

All of the above results indicate that platinum(IV) prodrugs containing different sugars, different platinum cores, different chain lengths and positional isomers show significant cytotoxicity to cancer cells. Further, for 1c-6c and 1d-7d, the different trans-membrane mechanisms and different reduction potentials render them different from each other, perhaps enabling these compounds to have greater potential for further investigations with targeted therapy.

#### Docking studies of platinum(IV) prodrugs (6d)

**Figure 2:**
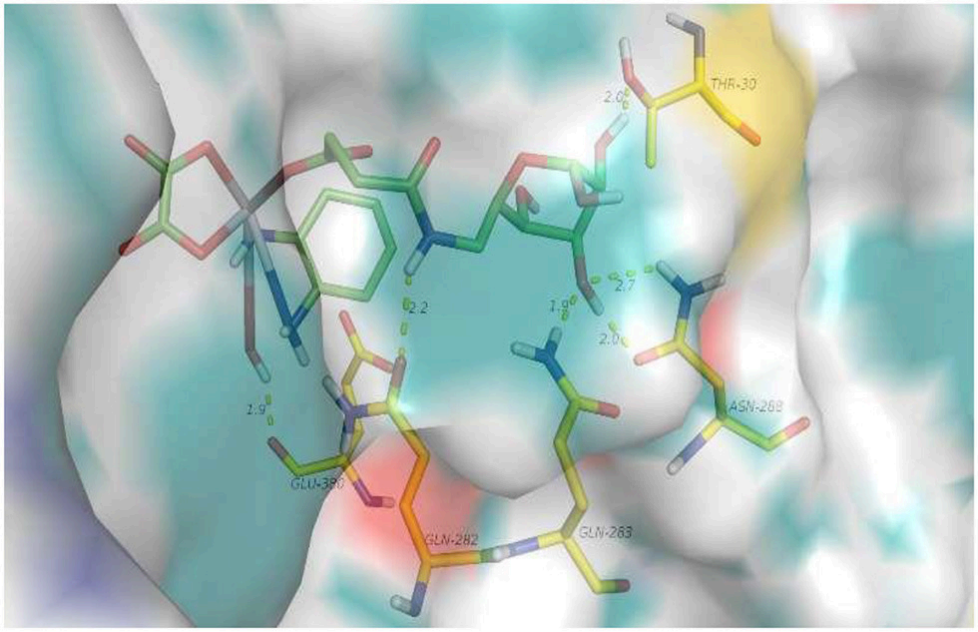
**6d** interacting with a variety of amino acid residues of GLUT1 (4PYP).

First, a docking study of 6d was performed to determine whether it can be transferred by target GLUT1. As shown in Figure 2, hydrogen-bonding interactions of 6d with GLUT1 occur with GLU-380, THR-30, GLN-263, and ASN-268. GLU-380 is capable of interacting with the hydroxy and amino of the platinum moiety. Hydrogen-bonding interactions of the 3-hydroxy and 4-hydroxy of O-6GlcAz can be found with THR-30, GLN-263, and ASN-268. The stable interaction of GLUT1 abundant in cancer cells would contribute greatly to the targeting of pyranoside conjugated platinum(IV) complexes (6d).

#### Effects of EDG(1), phloretin(2), Ctd.(3), and the mixture of EDG and Ctd.(4) on the IC_50_ values and cellular uptake of MCF-7 cells

For cytotoxicity assays, the GLUT1 inhibitors EDG (1) and phloretin (2), the OCT2 inhibitor Ctd. (cimetidine) (3), and the mixture of EDG and Ctd.(4) were selected to undergo a similar procedure as antitumor activity *in vitro*. MCF-7 cells were seeded on a 96 well plate in 100 μL of RPMI and were incubated for 24 h at 37°C, and EDG-containing RPMI medium, phloretin-containing RPMI medium and Ctd.-containing RPMI medium were used to render the serial dilution of the concentrated solutions of the platinum compounds, and 100 μL/well was added (resulting in final inhibitor concentrations of 75 mM, 100 μM and 3 mM). The cytotoxicity profiles of the compounds were evaluated using MTT assay.

**Figure 3:**
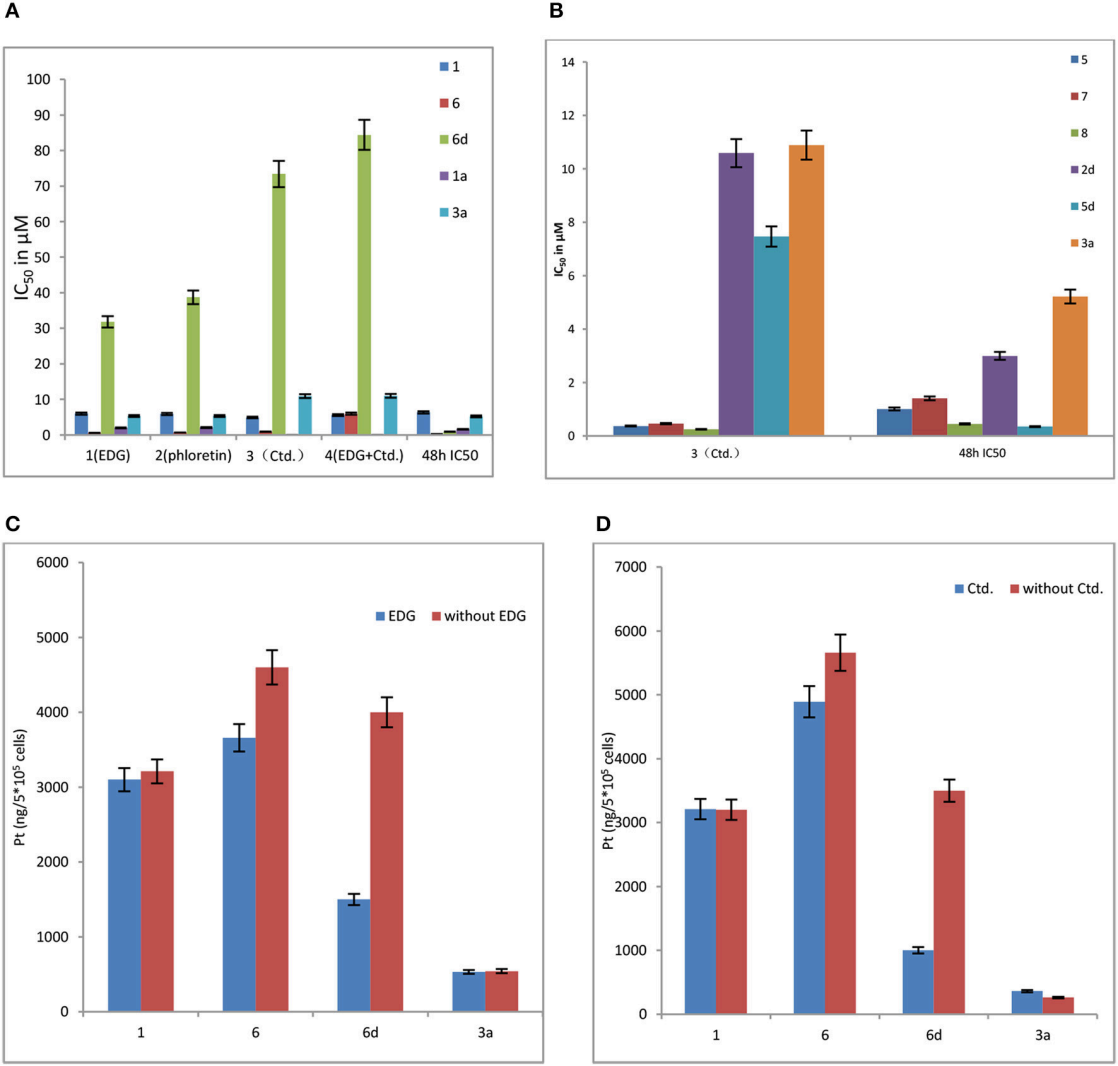
**(A)** Effects of EDG (1), phloretin (2), Ctd. (3), and a mixture of EDG and Ctd. (4) on the IC_50_ values of compounds **1, 6, 6d, 1a**, and **3a** in MCF-7 cells. **(B)** Effects of Ctd.(3) on the IC_50_ values of on compounds **5, 7, 8, 2d, 5d**, and **3a** in MCF-7 cells. **(C)** Effects of EDG on the cellular uptake of compounds **1, 6, 6d**, and **3a** in MCF-7 cells. **(D)** Effects of Ctd. on the cellular uptake of compounds **1, 6, 6d**, and **3a** in MCF-7 cells.

As shown in Figure 3A, 6d behaves significant sensitivity to GLUT1 with inhibition of EDG and phloretin. The IC_50_ values of **6d** with EDG and phloretin (31.80 and 38.71 μM) are 36- and 44-folds higher, respectively, than those of 48 h IC_50_ (0.89 μM), indicating that the targeting of GLUT1 mediates the transport. We previously reported the glycosylated platinum(IV) complexes **1** (IC_50_ = 6.36 μM) and **6** (IC_50_ = 0.19 μM) (Ma et al., 2017a). The IC_50_ values of **1** and **6** with EDG and phloretin are **0-3** folds higher than the IC_50_ without inhibitor. The extent of cellular inhibition of Glc-Pts in the presence of EDG and phloretin is in the order of **6d** >> **6** > **1** (Figure 3A), and this trend tracks with the cellular uptake of these compounds (Figure 3C), providing further support for the proposed GLUT1 translocation efficiencies for C6-glucose conjugates. EDG and phloretin do not affect compounds **1a** and **3a**, as evidenced by their IC_50_ values, which were not greatly different. The results remind us that, when we design the sugar-platinum(IV) complexes, C6-modified oxaliplatin pro-drugs are candidates showing close relationships with the transporters. In addition, as shown in Figures 3A,B,D, Ctd. for the inhibition of OCT2 also works with **2d**, **5d**, and **6d**. The IC_50_ values of **6d** with Ctd. (3) and the mixture of EDG and Ctd. (4) are 82- and 95-folds higher, respectively, than that the 48 h IC_50_ (Figure 3A). The IC_50_ of **6d** was further decreased by 59% after treatment with a mixture of EDG and Ctd., compared with treatment with EDG alone. These results further confirm the involvement of GLUT1 and OCT2 in the cellular uptake and cellular accumulation of **6d**. The extent of the cellular inhibition of Glc-Pts in the presence of Ctd. is in the order of **6d** >> **6** > **1** (Figures 3A,C), and this trend tracks with the cellular uptake of these compounds (Figure 3D). Because energy-dependent organic cation transporters (OCTs) contribute to the cellular uptake of **6** and **6d**, we propose that the differential uptake induced by the presence of EDG most likely arises from the energy-depleted conditions produced by glucose transport inhibition.

Organic cation transporter 2 (OCT2) plays important roles in the cellular accumulation and consequent cytotoxicity of platinum complexes containing the (1R,2R)-cyclohexane-1,2-diamine (DACH) ligand (Zhang et al., 2006). Because the Glc-Pts reported here bear the chelating DACH ligand, we investigated the potential of **2d** and **5d** to undergo translocation *via* OCT2 (Figure 3B), which is a transporter overexpressed in certain types of cancer cells and tumor samples from patients (Zhang et al., 2006; Burger et al., 2010).

The above observations indicate that 2d, 5d and the C6-modified glycoside compound (6d) are superior for targeting transport. Moreover, these above results also suggest that the pyranoside conjugated platinum(IV) complexes have enhanced cancer-targeting properties, providing new insight into the potential of the glycosylated Pt(IV) anticancer prodrugs.

#### Viability of the cancer cells MCF-7, HeLa, and the normal cells 293T and 3T3 in a 48 h incubation assay

**Figure 4:**
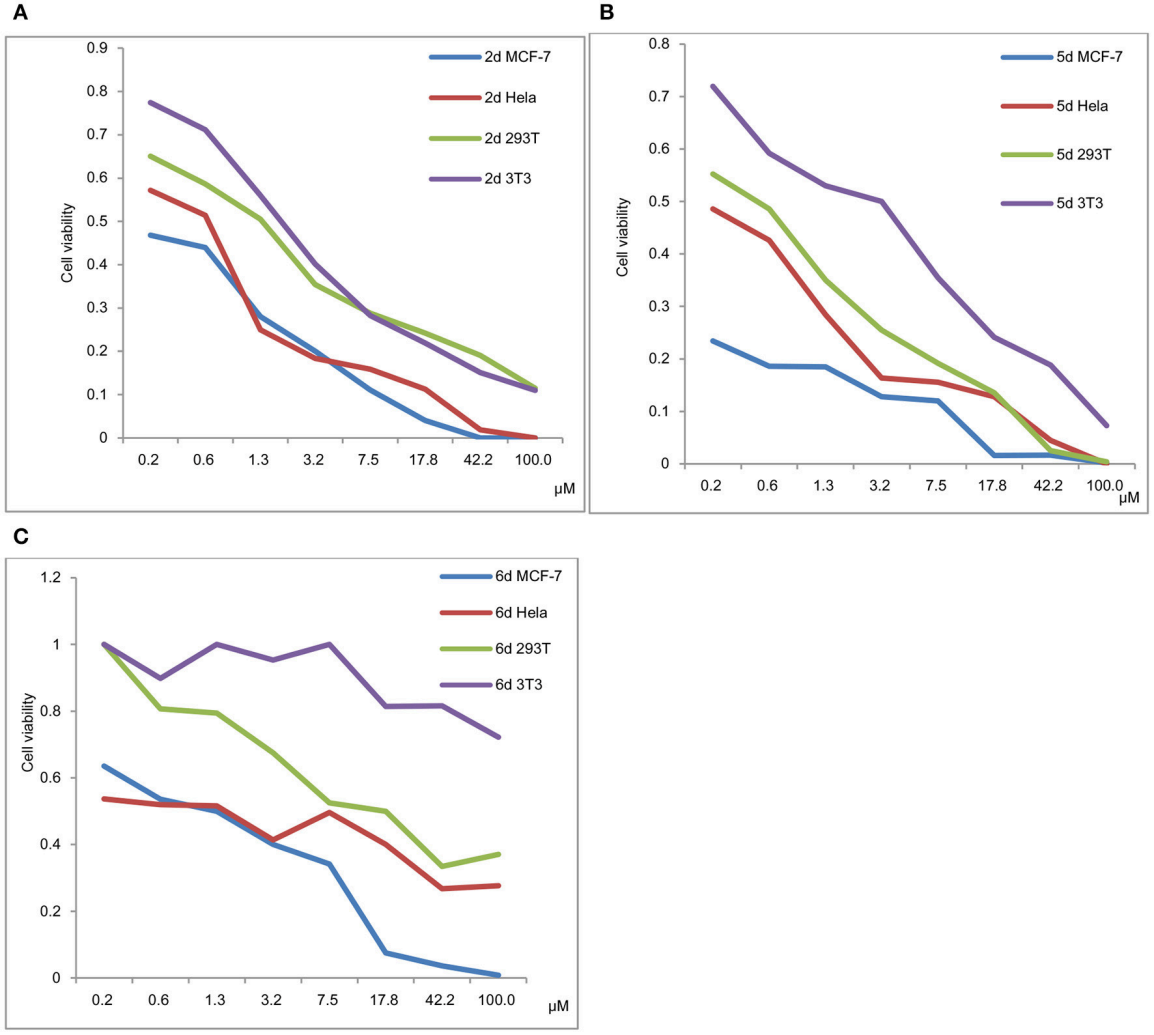
**(A)** Viability of MCF-7, HeLa, 293T and 3T3 in a 48 h incubation assay (**2d**). **(B)** Viability of MCF-7, HeLa, 293T and 3T3 in a 48 h incubation assay (**5d**). **(C)** Viability of MCF-7, HeLa, 293T and 3T3 in a 48 h incubation assay (**6d**).

An ideal anticancer compound should be selective for cancer cells over normal healthy cells, thereby mitigating the undesired toxic side effects associated with chemotherapy. We, therefore, evaluated the selectivity of 2d, 5d, and 6d using the cancer cell lines MCF-7 and HeLa and the normal cells 293T and 3T3 (Figure 4).

The viability of MCF-7, HeLa, 293T and 3T3 in a 48 h incubation assay for 2d, 5d, and 6d is shown in Figures 4A–C, respectively. The results exhibited that the IC_50_ values of 293T and 3T3 were higher than those of MCF-7 and HeLa for 2d, 5d, and 6d. We can deduce that de-protection of the mono-functionalized platinum(IV) prodrugs 2d, 5d, and 6d reduced the viability of cancer cells more efficiently than that of normal cells. As shown in Figure 4, despite the cytotoxicity of 6d being lower than 2d and 5d for tested cancer cells, decreased toxicity can be observed for 6d to the normal cells (293T IC_50_ = 12.06 μM and 3T3 cells IC_50_ > 100 μM).

**Figure 5:**
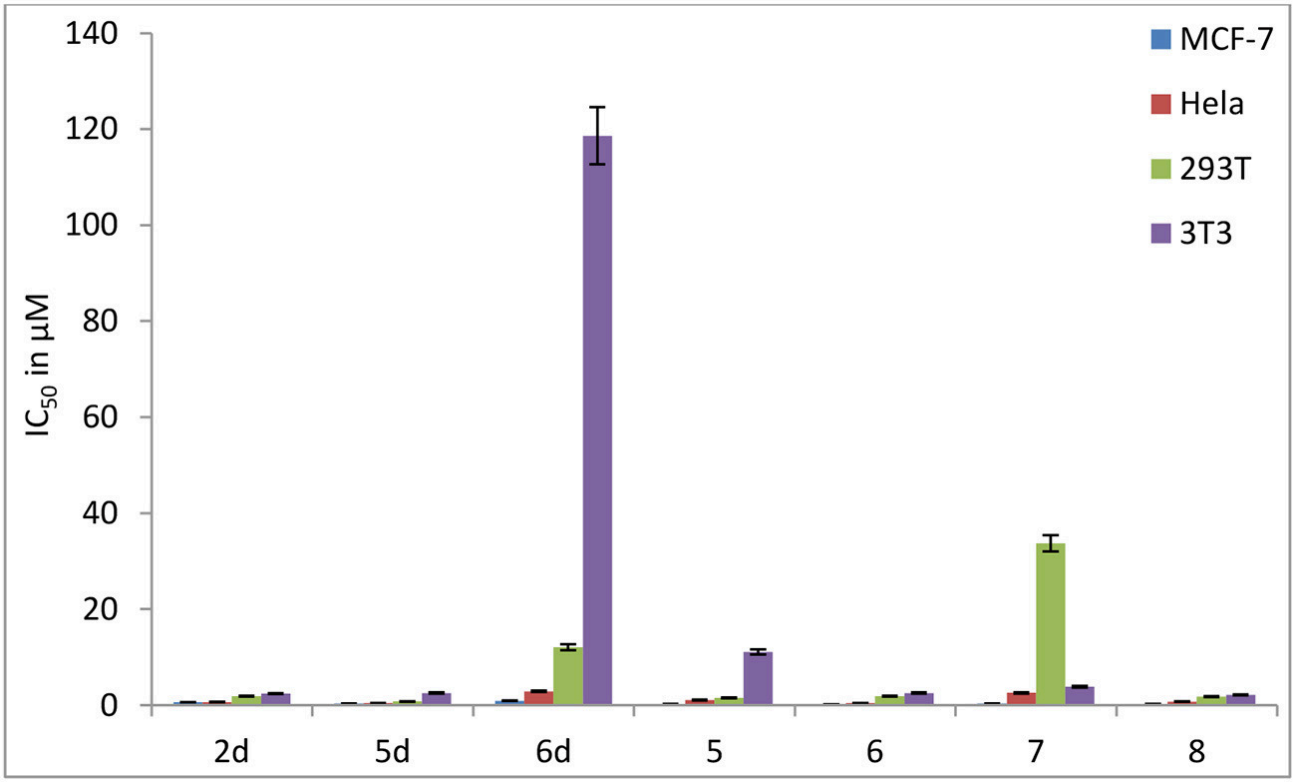
Inhibitory effect (IC_50_ in μM) of carbohydrate platinum(IV) complexes **2d**, **5d**, **6d**, **5**, **6**, **7**, and **8** on cancer cells and normal cells (3T3 and 293T).

In contrast to the previously reported lipophilic leading compounds 5-8,^15a^ the cytotoxicity of 2d, 5d, and 6d is comparable for HeLa and MCF-7 cells. The results above show that there is a significant dependency between the cytotoxicity of the platinum(IV) complexes 2d, 5d, and 6d and the transporters. Moreover, tumor-specific and transporter-targeted sugar conjugates would possess more favorable toxicity profiles and enhanced tumor selectivity and antitumor potency due to GLUT and OCT-mediated drug uptake, which is desired in antitumor agents for targeted therapy drugs. As shown in Figure 5, compared with the previously reported lipophilic leading compounds 5-8, hydrophilic compounds **2d, 5d**, and **6d** demonstrated the deduced toxicity profiles. In the particular, the leading compound 6d, the IC_50_ of which with EDG and phloretin (31.80 and 38.71 μM) is 36- and 43-folds higher, respectively, than the 48 h IC_50_ (0.89 μM), exhibits enhanced tumor selectivity and antitumor potency with normal cells (293T IC_50_ = 12.06 μM and 3T3 cells IC_50_ > 100 μM).

#### Stability of 6d in RPMI 1640 and whole human blood

**Figure 6:**
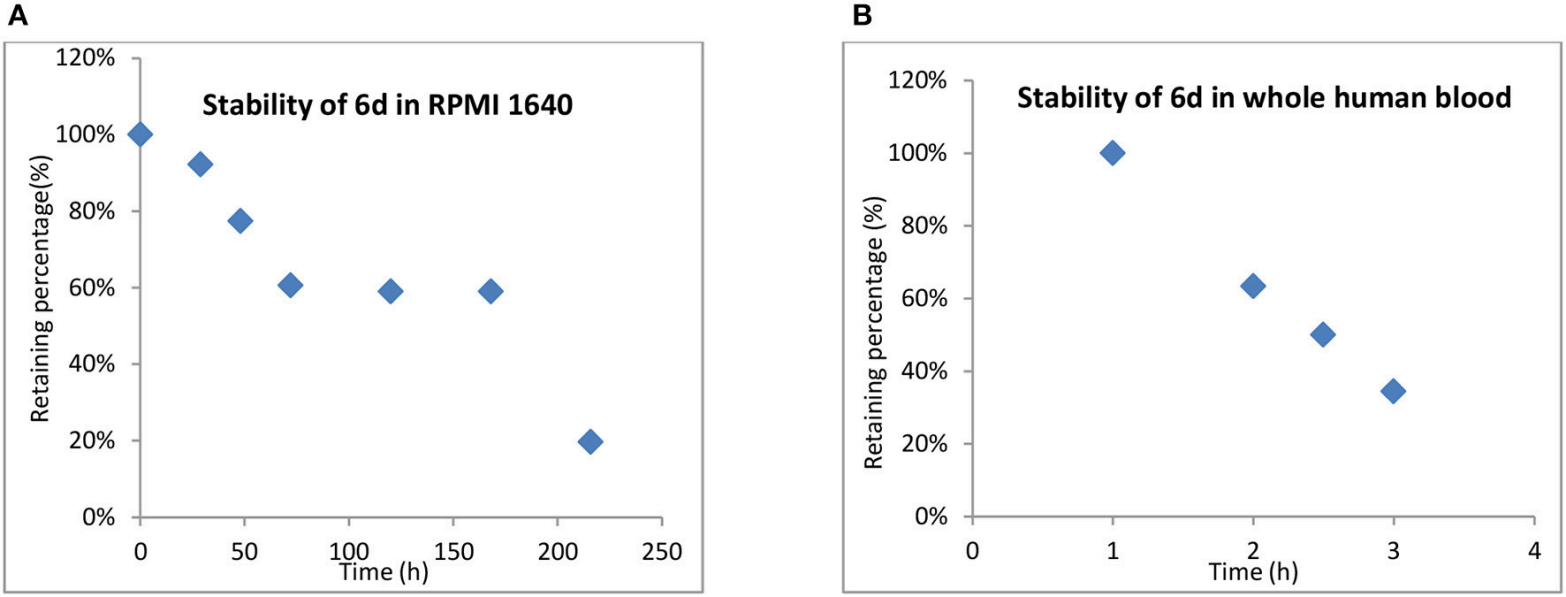
Stability of **6d** in RPMI 1640 **(A)** and whole human blood **(B)**.

One of the advantages of inert Pt(IV) prodrugs is their enhanced stability in RPMI 1640 and whole human blood. Like the result shown in Figure 6, the stability of 6d in RPMI 1640 and whole human blood was measured.

In RPMI medium, slow activation of 6d by the nucleophiles present in the medium was observed. No significant decomposition was shown at up to 12 h, 66% of 6d remained unchanged even after 48 h, and the half-life up to 170 h in RPMI 1,640 suggested that the compound was highly stable in biological media and was superior to platinum(II) drugs. (Figure 6). **6d** also showed more stability in whole human blood, with the t_1/2_ almost up to 2.5 h compared with 21.6 and 6.3 min for cisplatin and satraplatin, respectively.

#### Whole cell uptake studies and DNA platination for HeLa and MCF-7 cells

**Figure 7:**
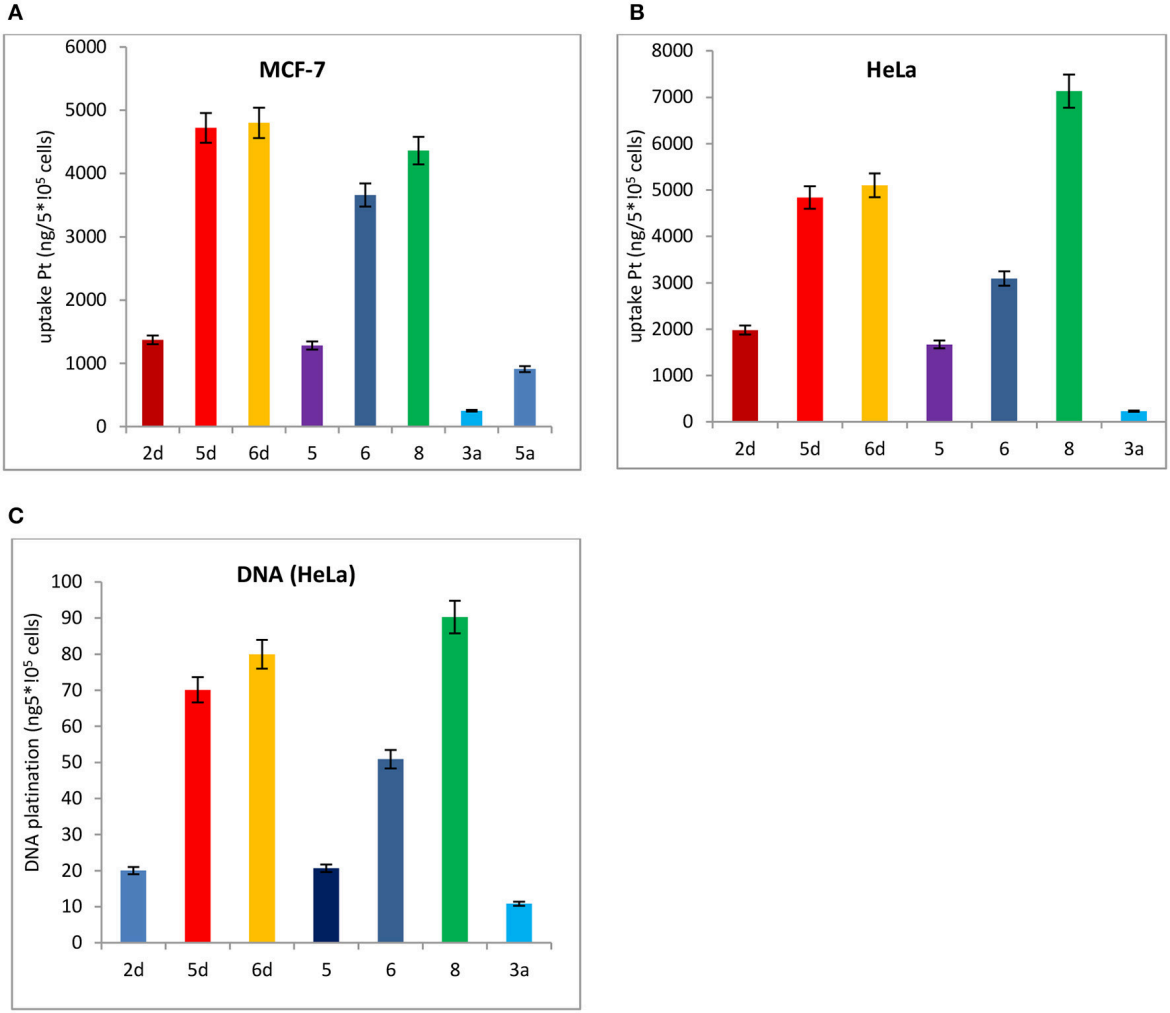
Cell uptake and DNA platination of **2d, 5d, 6d, 5, 6, 8, 3a**, and **5a**. **(A)** Cell uptake of **2d, 5d, 6d, 5, 6, 8, 3a**, and that the compound was in MCF-7 cells. **(B)** Cell uptake of **2d, 5d, 6d, 5, 6, 8, 3a**, and **5a** in HeLa cells. **(C)** DNA platination of **2d, 5d, 6d, 5, 6, 8, 3a**, and **5a** isolated from HeLa cells.

Subsequently, the cell uptake and DNA platination of the leading compounds **2d, 5d, 6d**, and the positive controls **3a** and **5a** with potent cytotoxicity to HeLa and MCF-7 cells are quantified by inductively coupled plasma mass spectrometry (ICP-MS) using ^196^Pt detection, as shown in Figure 7. The reported leading compounds **5, 6**, and **8** are also listed in Figure 7.

The cell uptake and DNA platination of **2d, 5d, 6d, 5, 6**, and **8** are all superior to those of the positive controls **3a** and **5a**. Compared with the reported leading compounds **5, 6** and **8**, the cell uptake and DNA platination of 5d and 6d are superior to those of 5, 6, and 8. After incubation with **2d, 5d**, and **6d**, the accumulation of platinum in MCF-7 cells is 5- to 19-fold and 2- to 5-fold higher, respectively, than that of oxaliplatin (**3a**) and satraplatin (**5a**). In HeLa cells, they are 2- to 21-fold higher than that of oxaliplatin (**3a**) and satraplatin (**5a**). The platinum in DNA isolated from HeLa cells was also measured and was shown to be 2- to 7-fold higher than that from oxaliplatin (**3a**) and satraplatin (**5a**).

#### Reduction and DNA binding properties of 6d and oxaliplatin with and without ascorbic acid

**Figure 8:**
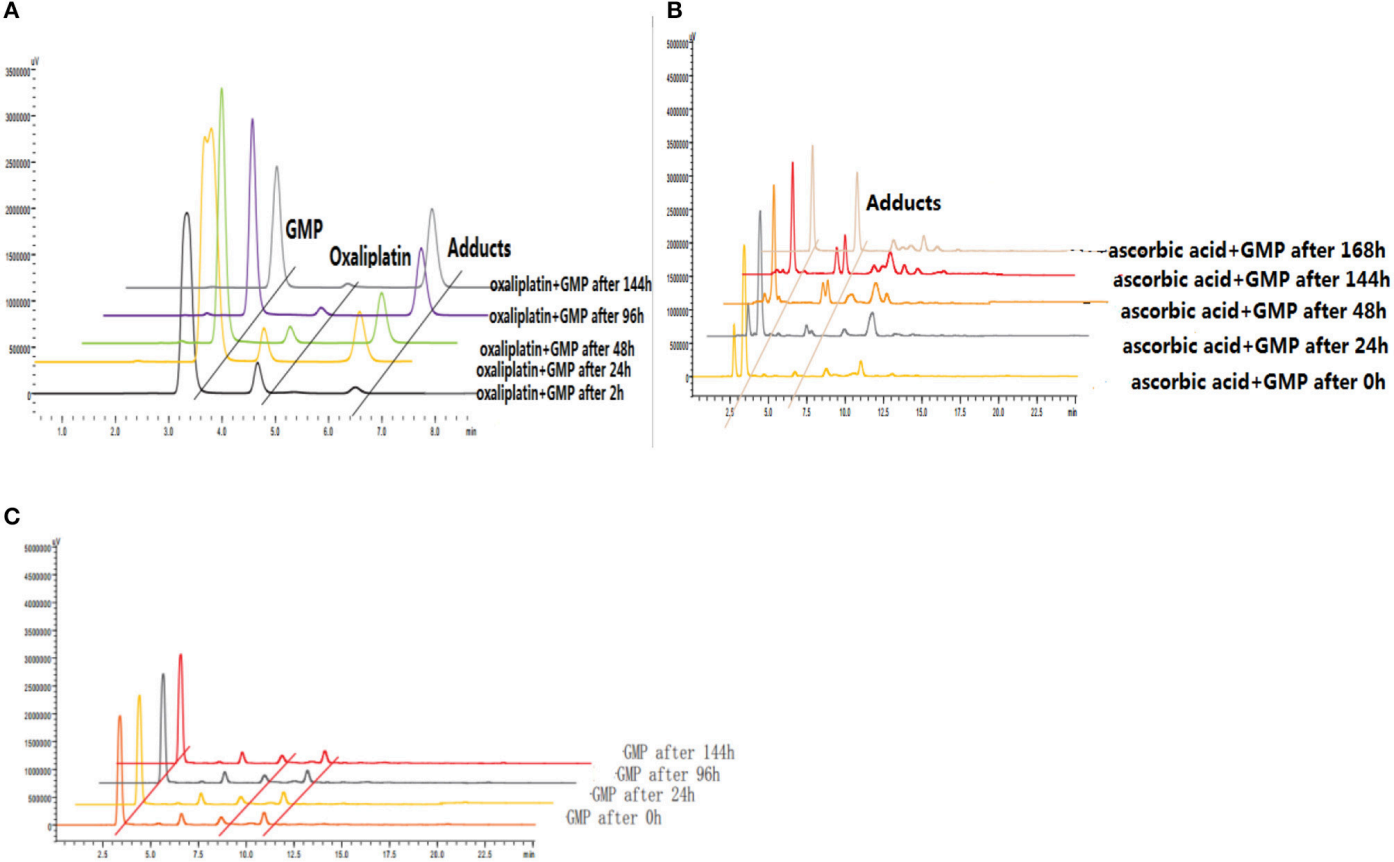
**(A)** Reduction and DNA-binding properties of oxaliplatin after 2, 24, 48, 96, and 144 h. **(B)** Reduction and DNA-binding properties of **6d** with ascorbic acid after 0, 24, 48, 144, and 168 h. **(C)** Reduction and DNA-binding properties of **6d** without ascorbic acid after 0, 24, 96, and 144 h.

The reduction and DNA-binding properties of **3a** and **6d** with and without ascorbic acid after 0, 2, 24, 48, 96, and 144 h were measured to determine the potential reduction mechanism of **6d** (Figure 8). The unknown peak fractions were separated and identified by ESI-MS analysis, and the results showed the same products as with oxaliplatin. The bis-substituted products originating from the combination of oxaliplatin with 5′-dGMP were generated after reduction by reductants.

#### Biodistribution of Rha-Pt (2d), Gal-Pt (5d), Glc-Pt (6d), and satraplatin (5a) *in vivo*

**Figure 9:**
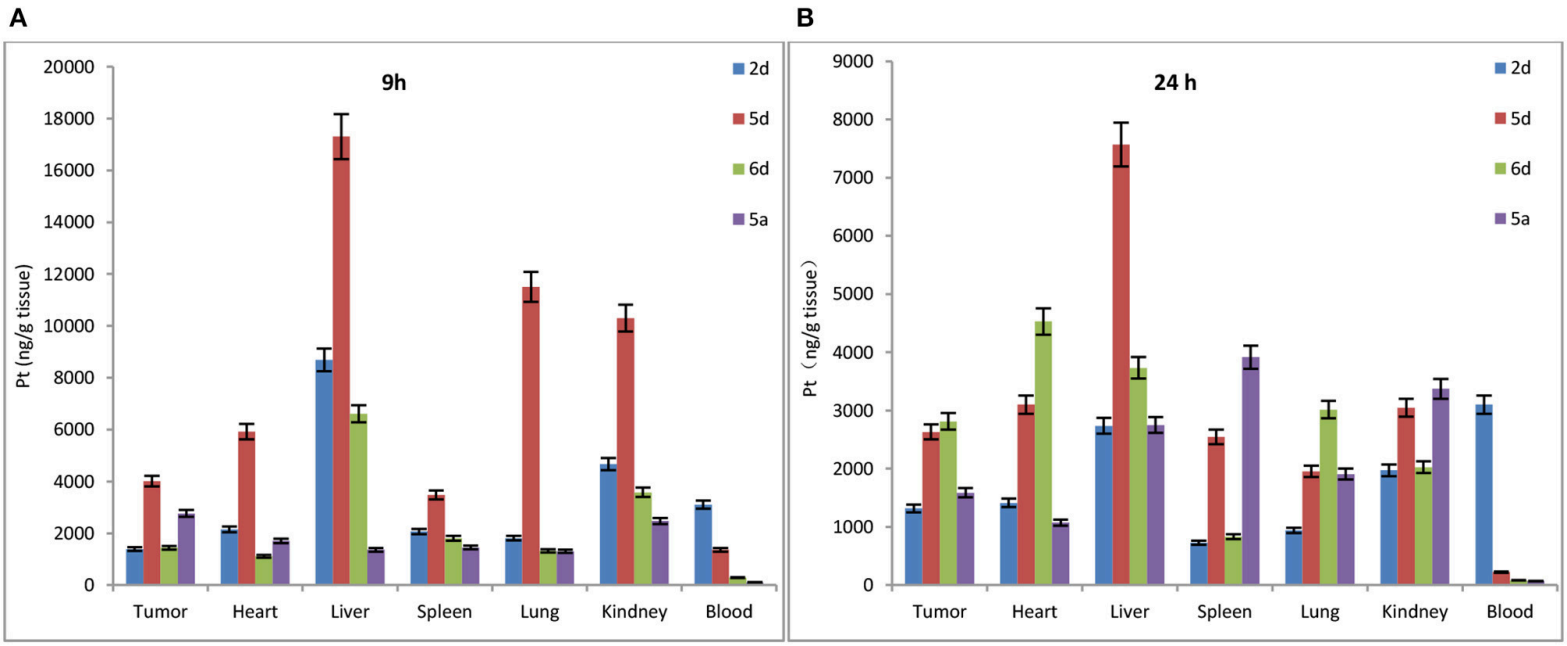
Biodistribution of Rha-Pt (**2d**), Gal-Pt (**5d**), Glc-Pt (**6d**), and satraplatin (**5a**) *in vivo* for 9 h **(A)** and 24 h **(B)**.

To determine whether de-protection of mono-functionalized platinum(IV) prodrugs for targeted therapy with significant GLUT1 and OCT2 substrates can also perform cancer targeting *in vivo*, the biodistribution of Rha-Pt (**2d**), Gal-Pt (**5d**), Glc-Pt (**6d**), and satraplatin (**5a**) as positive controls in MCF-7 bearing animals were assessed by ICP-MS at 9 and 24 h post-administration (i.v.). The IC_50_ values of Rha-Pt (**2d**), Gal-Pt (**5d**), Glc-Pt (**6d**), and satraplatin (**5a**) are 0.61, 0.35, 0.89 and 8.01 μM *in vitro*, respectively, which are not greatly different from that of MCF-7. Surprisingly, accumulation of Pt in Gal-Pt (**5d**) and Glc-Pt (**6d**) from tumor tissue is 2-fold higher than that of satraplatin (**5a**) after 24 h of incubation, as shown in Figures 9A,B, showing a great difference from the results *in vitro*. The encouraging selectivity of Gal-Pt (**5d**) and Glc-Pt (**6d**) to MCF-7 cells *in vivo* indicates that the pyranoside performs an important function of cancer targeting and has decreased toxicity in platinum(IV) prodrugs *in vivo*.

#### Effects of EDG (1), phloretin (2), Ctd. (3), and the mixture of EDG and Ctd. (4) on the IC_50_ values of MDCK cells

**Figure 10:**
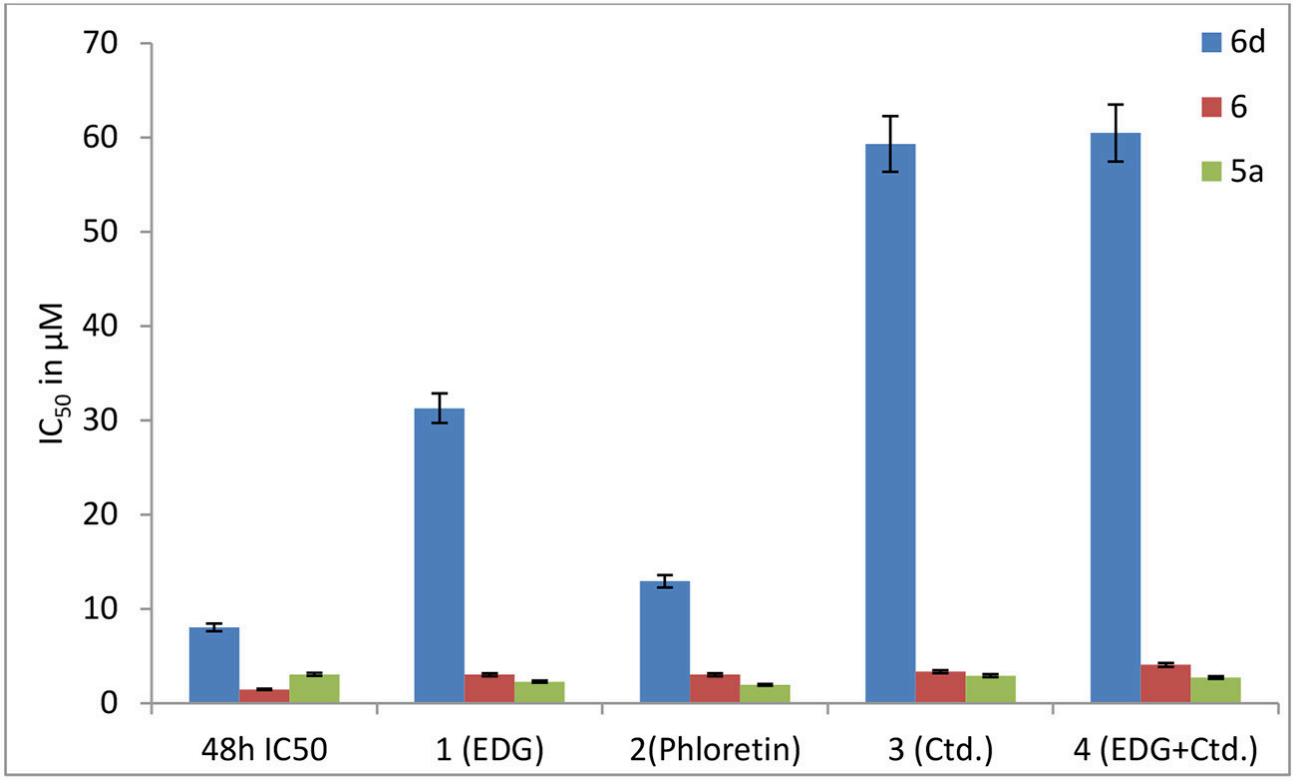
Effects of EDG (1), phloretin (2), Ctd. (3), and the mixture of EDG and Ctd. (4) on the IC_50_ values of **6, 6d** and **5a** in MDCK cells.

Finally, Madin-Darby canine kidney (MDCK) cells, which are widely used to establish the model of the intestinal epithelium and to evaluate the oral administration and screening of substrates and inhibitors *in vitro*, were used to find a prodrug with the enhanced potential for oral administration. To determine whether targeted transporters are effective for MDCK cells, we investigated the mechanisms of **6d** and **5a**, which were also co-incubated with the GLUT1 inhibitors 4,6-oethylidene-α-D-glucose (EDG) and phloretin, the OCT2 inhibitor Ctd., and the combination of EDG and Ctd. (Figure 10).

The results show that the IC_50_ values of **6d** with EDG (1), phloretin (2), Ctd. (3) and EDG + Ctd. (4) are higher than 48 h MTT assays, indicating close relationships of **6d** with the transporters. However, the IC_50_ values of the positive control (**5a**) co-cultured with the GLUT1 inhibitors EDG (1) and phloretin (2), the OCT2 inhibitor Ctd. (3) and the combination of EDG (1) and Ctd. (3) resulted in no great differences from the IC_50_ at 48 h. It is worth mentioning that **6d**, the IC_50_ of which with 1 (EDG), 3 (Ctd.), and 4 (EDG + Ctd.) is 4- to 8-fold higher than that at 48 h, shows more close relationships with GLUTs and OCTs of MDCK cells than with the reported leading compound **6**, indicating an advanced interval to the oral switching of Pt(IV) prodrugs.

## Discussion

What we can do to develop oral platinum(IV) prodrugs is design a potent series of small molecule platinum(IV) drugs with enhanced cytotoxicity to cancer cells and with the following properties: (1) effects across membranes with mechanical barriers *via* enhanced permeability and retention (EPR) or transporter overexpression on the surface of intestinal membranes and cancer cells; (2) improved stability in human blood and increased blood-circulation time to prevent premature activation and induce to apoptosis in cancer cells primarily through DNA damage; and (3) preferential accumulation in cancer cells and decreased toxicity to normal cells. In our present study, protected and de-protected mono-functionalized platinum(IV) prodrugs as significant GLUT1 and OCT2 substrates for targeted therapy were designed, synthesized and evaluated for detailed mechanisms *in vitro* and *in vivo*. Overall, **2d**, **5d**, and **6d** were more effective than the positive controls cisplatin (**1a**), oxaliplatin (**3a**), and satraplatin (**5a**), and **1d**-**7d** with different pyranosides, such as glucose, galactose, mannose, and rhamnose, also showed specific tumor targeting *in vitro*. They also demonstrated the potential to exploit the two transporters GLUT1 and OCT2 of MCF-7 and MDCK, both of which are overexpressed on the surface of tumor cells. Moreover, preferentially accumulating in and annihilating cancer cells over noncancerous cells (293T and 3T3 cells) *in vitro* indicated low toxicity of antineoplastic chemotherapy with platinum drugs. The biodistribution of **6d** *in vivo* made a great difference from the results *in vitro*. The encouraging selectivity of **6d** *in vivo* to the MCF-7 tumor cells could contribute to the design of platinum(IV) prodrugs with facilitated intravenous to oral switching. All of the results above showed great differences and enhanced effects of cancer targeting clinically.

## Materials and methods

### Experimental section

#### HPLC studies

The purities of all of the target compounds were determined by HPLC. The HPLC analyses were also performed as on a Waters E2695-2998 equipped with a Venusil MP C18 column (150×4.6 mm, 5 μM). The purity of the platinum complexes was confirmed to be ≥95% by analytical HPLC (in the Supplementary Material). The linear gradient was as follows (Table 2).

**Table 2:** Methods of the HPLC analyses for the purities of all of the target compounds.

**Time(min)**	**A (water)**	**B (methanol)**
	90	10
5	90	10
35	0	100
45	0	100

#### General procedure for the synthesis of the targeted compounds 1c-6c and 1d-7d

##### Synthesis of target compounds

Compounds **1c**-**6c** were prepared by forming an amide bond in the reaction between per-*O*-acetyl 1-aminosugars and Pt(IV) complexes (**1b** or **2b**) with a carboxylic function in a succinic spacer (Scheme 1). The key step involved the preparation of oxoplatins **2a** and **4a**, which were reacted successively in water (12 mL) with cisplatin (0.2 g) or oxaliplatin (0.2 g) and H_2_O_2_ (20 mL) at 60°C with yields of 60% and 55%, respectively. **1b** and **2b** were prepared from oxoplatin **2a** (1 equiv) or **4a** (1 equiv) and succinic anhydride (1 equiv) in anhydrous DMSO at room temperature overnight. DMSO was removed under a vacuum to yield a yellow oil, and the product was washed with acetone and diethylether and dried in a vacuum. The target compounds **1b** and **2b** were obtained as pale yellow solids after purification by recrystallization with yields of 75% and 78%, respectively. Finally, to a solution of **1b** or **2b** (1 equiv) in DMF was added a DMF solution containing HATU. The mixture was stirred for 10 min at room temperature. A DMF solution containing amines and DIPEA was added to the resulting solution to obtain **1c**-**6c** and **1e**-**7e** in yields of 20% to 50%. The benzyl group of **1e**-**7e** was removed in anhydrous DCM, BCl_3_ (1 M) (0.80 mL, 0.80 mmol) at −78° for 2 h. The DCM was removed and purified by column chromatography to obtain **1d**-**7d**. The mixture was stirred at room temperature for 24 h in the dark. The product was characterized using ^1^H-NMR, ^13^C-NMR, ESI-MS spectrometry and elemental analysis (see ESI^+^).

##### Preparation of 1c

The complex **1c** was synthesized as previously described. To a solution of **1b** (1 equiv) in DMF was added a DMF solution containing HATU (1.5 equiv). This mixture was stirred for 10 min at room temperature. To the resulting solution was added a DMF solution containing **5b** (1.2 equiv) and DIPEA (2.4 equiv). The mixture was stirred at room temperature for 24 h in the dark. The DMF was then removed under a vacuum to yield a yellow oil. Compound **1c** was purified by silica gel column chromatography as a yellow solid with a yield of 20%. ^1^H NMR (400 MHz, methanol-d4) δ 5.47–5.32 (m, 1H), 5.29–5.08 (m, 1H), 5.00 (ddd, *J* = 14.1, 10.3, 5.8 Hz, 1H), 4.79–4.59 (m, 1H), 4.43 (t, *J* = 12.1 Hz, 1H), 4.22–4.01 (m, 3H), 3.99–3.84 (m, 1H), 3.81–3.62 (m, 1H), 3.26–2.38 (m, 4H), 2.36–0.85 (m, 24H); and ^13^C NMR (400 MHz, methanol-d4) δ 171.02, 170.98, 170.65, 170.57, 170.55, 170.40, 170.05, 169.77, 169.32, 100.67, 100.60, 88.90, 76.60, 75.62, 71.00, 70.42, 69.29, 67.21, 62.36, 61.94, 60.95, 54.45, 42.50, 19.83, 19.74, 19.30, 19.26, 19.21, 19.10, 18.97. HRMS: Calcd. for C_30_H_47_Cl_2_N_3_O_20_Pt (M+OH^−^): 1051.1811, found: 1051.1212.

##### Preparation of 2c

**2c** was synthesized according to **1c** with a yield of 35%. ^1^H NMR (400 MHz, methanol-d4) δ 5.36 (d, *J* = 3.2 Hz, 4H), 4.69 (d, *J* = 7.9 Hz, 1H), 4.43 (s, 1H), 4.31–4.00 (m, 4H), 3.94 (m, 2H), 3.12–2.96 (m, 2H), 2.73–2.49 (m, 6H), 2.29–1.81 (m, 22H), 1.65 (s, 4H), 1.34 (dd, *J* = 19.1, 13.1 Hz, 5H); and ^13^C NMR (400 MHz, methanol-d4) δ 172.43, 172.37, 172.02, 171.95, 171.79, 171.51, 171.44, 171.15, 170.70, 102.07, 90.14, 76.94, 72.47, 72.23, 71.81, 71.10, 70.96, 70.69, 68.59, 63.24, 62.33, 35.50, 32.60, 32.35, 25.09, 21.12, 20.73, 20.68, 20.63, 20.59, 20.48, 20.35. HRMS: Calcd. for C_38_H_55_N_3_O_24_Pt (M+2NH_3_): 1166.3354, found: 1166.3051.

##### Preparation of 3c

**3c** was synthesized according to **1c** with a yield of 25%. ^1^H NMR (400 MHz, methanol-d4) δ 5.80–5.19 (m, 3H), 5.06 (dd, *J* = 19.5, 9.6 Hz, 1H), 4.60–4.41 (m, 1H), 4.38–4.18 (m, 2H), 4.15–3.45 (m, 6H), 3.18–2.39 (m, 3H), 2.36–0.96 (m, 21H); and ^13^C NMR (400 MHz, methanol-d4) δ 172.29, 172.00, 171.75, 171.59, 171.53, 171.45, 171.18, 97.14, 78.55, 77.11, 75.18, 74.81, 71.65, 70.67, 69.82, 69.63, 64.32, 63.02, 61.53, 32.84, 22.73, 21.20, 20.88, 20.77, 20.71, 20.65, 20.56, 14.31. HRMS: Calcd. for C_30_H_47_Cl_2_N_3_O_20_Pt (M+OH^−^): 1051.1811, found: 1051.1768.

##### Preparation of 4c

4c was synthesized according to **1c** with a yield of 35%. ^1^H NMR (400 MHz, methanol-d4) δ 5.51–5.09 (m, 4H), 4.97 (t, *J* = 9.8 Hz, 1H), 4.77–4.66 (m, 2H), 4.63 (d, *J* = 10.1 Hz, 1H), 4.48–4.33 (m, 1H), 4.17 (dd, *J* = 12.4, 9.5 Hz, 3H), 4.08–3.83 (m, 3H), 3.79–2.09 (m, 10H), 2.07–1.86 (m, 21H), 1.59 (m, 4H); and ^13^C NMR (400 MHz, methanol-d4) δ 183.09, 175.55, 172.32, 172.29, 171.97, 171.75, 171.59, 171.54, 171.15, 167.32, 167.26, 97.11, 78.66, 76.97, 75.23, 74.78, 72.68, 71.64, 70.61, 69.79, 69.62, 64.34, 63.01, 62.24, 32.49, 32.41, 32.28, 25.44, 25.09, 22.84, 21.23, 20.88, 20.82, 20.80, 20.70, 20.59. HRMS: Calcd. for C_38_H_55_N_3_O_24_Pt (M+2NH_3_): 1166.3354, found: 1166.3114.

##### Preparation of 5c

**5c** was synthesized according to 1c with a yield of 40%. ^1^H NMR (400 MHz, methanol-d4) δ 5.53–5.13 (m, 3H), 4.47 (m, 1H), 4.21–3.99 (m, 2H), 3.60 (d, *J* = 7.0 Hz, 1H), 3.02–2.72 (m, 2H), 2.69–2.42 (m, 5H), 2.35–1.81 (m, 12H), 1.27 (ddd, *J* = 86.5, 76.4, 25.1 Hz, 8H); and ^13^C NMR (400 MHz, methanol-d4) δ 174.41, 172.03, 171.91, 158.02, 79.28, 73.39, 69.80, 68.89, 62.55, 61.48, 32.68, 32.63, 32.53, 32.46, 25.12, 20.62, 20.60, 20.56, 20.51. HRMS: Calcd. for C_26_H_39_N_3_O_17_Pt (M^+^): 860.1927, found: 860.1949.

##### Preparation of 6c

**6c** was synthesized according to **1c** with a yield of 48%. ^1^H NMR (400 MHz, methanol-d4) δ 5.64–5.11 (m, 4H), 4.16 (ddd, *J* = 14.1, 11.4, 5.8 Hz, 2H), 3.87 (s, 1H), 3.20–2.22 (m, 8H), 2.01 (ddd, *J* = 29.8, 29.0, 22.3 Hz, 12H), 1.86–0.80 (m, 7H); and ^13^C NMR (400 MHz, methanol-d4) δ 172.45, 172.36, 171.56, 157.05, 77.78, 75.03, 73.09, 70.82, 66.89, 63.49, 32.48, 32.14, 31.68, 31.38, 25.16, 20.77, 20.63, 20.55, 20.53. HRMS: Calcd. for C_26_H_39_N_3_O_17_Pt (M^+^): 860.1927, found: 860.1976.

##### Preparation of 1d

The benzyl group of **1e** was removed with anhydrous DCM, BCl_3_ (1 M) (0.80 mL, 0.80 mmol) at −78° for 2 h. The DCM was removed and purified by column chromatography to obtain the white solid **1d** (87 mg, 48%). ^1^H NMR (400 MHz, methanol-d4) δ 5.07 (m, 1H), 4.78–4.34 (m, 1H), 3.87 (dd, *J* = 50.9, 36.9 Hz, 1H), 3.60 (d, *J* = 19.2 Hz, 1H), 2.68 (dd, *J* = 131.2, 41.9 Hz, 4H), 1.81–0.67 (m, 8H); and ^13^C NMR (400 MHz, methanol-d4) δ 174.89, 174.58, 101.66, 73.96, 72.32, 72.10, 69.88, 66.91, 42.87, 32.73, 31.36, 30.15, 17.93. HRMS: Calcd. for C_13_H_32_Cl_2_N_3_O_12_Pt (M+3OH^−^): 688.2020, found: 688.2085.

##### Preparation of 2d

**2d** was synthesized according to **1d** with a yield of 35%. ^1^H NMR (400 MHz, methanol-d4) δ 5.21 (s, 1H), 4.65 (m, 1H), 4.26–3.47 (m, 3H), 3.31 (m, 4H), 2.21 (ddd, *J* = 385.9, 203.1, 101.8 Hz, 19H); and ^13^C NMR (400 MHz, methanol-d4) δ 175.43, 174.91, 166.97, 101.64, 73.95, 72.28, 72.09, 69.89, 66.90, 42.96, 40.23, 32.56, 32.38, 31.36, 30.16, 24.99, 18.06. HRMS: Calcd. for C_21_H_39_N_3_O_15_Pt (M+2OH^−^): 768.1229, found: 768.1289.

##### Preparation of 3

**3d** was synthesized according to **1d** with a yield of 30%. ^1^H NMR (400 MHz, methanol-d4) δ 5.23 (d, J = 85.9 Hz, 1H), 4.77 (t, J = 9.3 Hz, 1H), 3.83 (d, J = 9.2 Hz, 1H), 3.70 (dd, J = 12.8, 8.6 Hz, 2H), 3.64–3.32 (m, 5H), 3.24 (m, 1H), 2.84–1.80 (m, 4H); and ^13^C NMR (400 MHz, methanol-d4) δ 151.15, 150.03, 101.74, 74.90, 72.62, 68.79, 67.22, 63.03, 61.65, 40.48, 32.51. HRMS: Calcd. for C_12_H_27_Cl_2_N_3_O_10_Pt (M^+^): 639.3430, found: 6 39.3399.

##### Preparation of 4d

**4d** was synthesized according to **1d** with a yield of 38%. ^1^H NMR (400 MHz, methanol-d4) δ 5.11 (d, *J* = 27.7 Hz, 1H), 4.79 (m, 1H), 3.84 (d, *J* = 9.6 Hz, 2H), 3.78–3.32 (m, 5H), 3.23 (dd, *J* = 14.7, 7.4 Hz, 1H), 2.93 (m, 6H), 1.82–1.08 (m, 8H); and ^13^C NMR (400 MHz, methanol-d4) δ 183.52, 175.29, 167.26, 167.19, 101.78, 74.87, 72.17, 68.76, 67.38, 63.01, 55.95, 43.86, 40.49, 32.70, 32.61, 25.27, 25.15, 18.81, 17.43, 13.33. HRMS: Calcd. for C_21_H_40_N_3_O_14_Pt (M+2H^+^): 753.2158, found: 753.2054.

##### Preparation of 5d

**5d** was synthesized according to **1d** with a yield of 38%. ^1^H NMR (400 MHz, methanol-d4) δ 5.70 (d, *J* = 65.2 Hz, 1H), 3.70 (dd, *J* = 67.9, 30.6 Hz, 6H), 3.32 (t, *J* = 25.8 Hz, 4H), 2.94–2.05 (m, 6H), 1.50 (dd, *J* = 132.6, 28.8 Hz, 10H); and ^13^C NMR (400 MHz, methanol-d4) δ 174.92, 174.82, 174.43, 113.54, 85.94, 76.88, 74.57, 72.41, 69.60, 60.33, 40.33, 37.33, 33.19, 32.52, 31.31, 30.17, 25.07. HRMS: Calcd. for C_21_H_37_N_3_O_14_Pt (M^+^): 750.1923, found: 750.1947.

##### Preparation of 6d

**6d** was synthesized according to **1d** with a yield of 38%. ^1^H NMR (400 MHz, methanol-d4) δ 5.35 – 4.87 (m, 1H), 4.32 (d, *J* = 7.3 Hz, 1H), 3.98–3.84 (m, 1H), 3.66–3.26 (m, 2H), 3.23–3.02 (m, 2H), 2.87 (t, *J* = 29.8 Hz, 6H), 1.85 (d, *J* = 12.3 Hz, 1H), 1.30–1.00 (m, 7H); and ^13^C NMR (400 MHz, methanol-d4) δ 173.06, 164.17, 164.04, 98.30, 94.10, 76.33, 76.01, 73.81, 73.65, 73.32, 71.57, 43.93, 40.47, 21.04, 19.44, 18.92, 17.48, 14.61, 12.95. HRMS: Calcd. for C_18_H_33_N_3_O_13_Pt (M+2H^+^): 694.1661, found: 694.1106.

##### Preparation of 7d

**7d** was synthesized according to **1d** with a yield of 38%. ^1^H NMR (400 MHz, methanol-d4) δ 5.31 (d, *J* = 43.0 Hz, 1H), 4.86–4.59 (m, 1H), 3.79 (ddd, *J* = 91.4, 70.8, 31.3 Hz, 5H), 3.29–1.96 (m, 6H), 1.44 (dd, *J* = 72.4, 66.3 Hz, 8H); and ^13^C NMR (400 MHz, methanol-d4) δ 200.89, 172.25, 169.78, 95.86, 73.94, 72.91, 72.28, 68.72, 62.62, 45.86, 30.82, 30.60, 30.31, 28.09, 25.08. HRMS: Calcd. for C_19_H_34_N_3_O_13_Pt (M^+^): 707.1739, found: 707.1690.

#### DNA-binding properties of oxaliplatin and 6d using RP-HPLC

To investigate the binding properties of DNA with Pt(II) complexes, 5′-GMP was used as a model of DNA. The DNA-binding properties of oxaliplatin (1 mM) and 5′-GMP (1 mM) at 37°C after 2, 24, 48, 96, and 144 h were determined using RP-HPLC. The results revealed new peaks of Oxp-Pt(II)-GMP (the conjugated complexes of oxaliplatin with 5′-GMP) generated by the mixture and demonstrated the potency of 5′-GMP to combine with Pt(II) complexes.

Further experiments were designed to test the reduction potential of the Pt(IV) complexes. The reduction and DNA-binding properties of **6** (1 mM) and 5′-GMP (3 mM) with and without ascorbic acid (5 mM) at 37°C after 0, 24, 48, 96, and 144 h were measured using RP-HPLC. The results proved that the glycosylated platinum(IV) complexes could be reduced by ascorbic acid and could release Pt(II) complexes. Then, the Pt(II) compounds combined with 5′-GMP to form OxpPt(II)-GMP, which was confirmed by HRMS. HPLC analyses were performed on a Waters E2695-2998 equipped with a Venusil MP C18 column (150 × 4.6 mm, 5 μm).

## Author contributions

All authors listed have made a substantial, direct and intellectual contribution to the work, and approved it for publication. JM, JW, SX, and PW contributed to the idea of the paper and put it into effect. HL, ZX, JH, and YL contributed to the antitumor activities *in vitro* and JN, TL, SB, XW, and CW *in vivo*. YL and TL contributed to the analysis, interpretation of data for the work. JW contributed to the design of the work.

### Conflict of interest statement

The authors declare that the research was conducted in the absence of any commercial or financial relationships that could be construed as a potential conflict of interest.

## Supplementary material

Data Sheet 1 Full experimental details, NMR data and bioassay information.

### PDB ID codes

Docked model of **6d** interacting with a variety of amino acid residues of GLUT1 (PDB code 4PYP) is shown in Figure 3 (PDB).

## Abbreviations

5′-GMP: 5′-guanosine monophosphate
GLUTs: Glucose transporters
OCTs: Organic cation transporters
HSA: Human serum albumin
SGLTs: Sodium-dependent glucose transporters
EDG: 4,6-oethylidene-a-D-glucose
Ctd.: Cimetidine
GLUT 1: Glucose transporter 1
OCT2: Organic cation transporter 2
DACH, (1R, 2R)-cyclohexane-1: 2-diamine
MDCK: Madine-darby canine kidney
DCM: dichloromethane
RPMI 1640: Roswell Park Memorial Institute 1640.
