# Supplementary material for: Protected and De-protected Platinum(IV) Glycoconjugates With GLUT1 and OCT2-Mediated Selective Cancer Targeting: Demonstrated Enhanced Transporter-Mediated Cytotoxic Properties in vitro and in vivo
Source: Front Chem. 2018 Sep 21;6:386. doi: 10.3389/fchem.2018.00386 (PMC6160541; doi:10.3389/fchem.2018.00386)
Supplement: Data Sheet 1 — Full experimental details, NMR data and bioassay information. [file Data_Sheet_1.PDF]

Electronic Supporting Information

for

Protected and de-protected platinum(IV) glycoconjugates with GLUT1 and OCT2-mediated selective cancer targeting: demonstrated enhanced transporter-mediated cytotoxic properties *in vitro* and *in vivo*

Jing Ma,[1] Hanfang Liu,[1] Zhuoqing Xi,[1] Jiuzhou Hou,[1] Yingguang Li,[1] Jie Niu,[1] Tong Liu,[1] Shuning Bi,[1] Xin Wang,[4] Chaojie Wang,[3] Jiajia Wang,[2,*] Songqiang Xie,[1,*] Peng George Wang[4,*]

[1] Institute of Chemical Biology, College of Pharmacy, Henan University, Kaifeng, 475004, China

[2] Henan University Joint National Laboratory for Antibody Drug Engineering; School of Basic Medicine Science, Henan University, Kaifeng, P.R. China

[3] The Key Laboratory of Natural Medicine and Immuno-Engineering, Henan University, Kaifeng, 475004, China

[4] College of Pharmacy, Tianjin Key Laboratory of Molecular Drug Research, State Key Laboratory of Elemento-organic Chemistry, Nankai University, Tianjin 300071 P.R. China

Table of Contents **Pages**

Experimental Details S3

Purity of target compounds S3

Synthetic procedures S4

In Vitro Cellular Cytotoxicity Assays S9

Cellular Platinum Uptake and DNA Platination S9

Biodistribution of Glc-Pt, Man-Pt, Gal-Pt and Satraplatin in MCF-7 bearing animals S10

Computational details S10

Reduction of Pt(IV) complexes by Vc S10

NMR spectra of compounds S11

References S39

Experimental Details

Materials

Cisplatin and oxaliplatin were purchased from Yurui Chemical Co. Ltd. (Shanghai, China). All of the other chemicals were obtained from commercial suppliers, such as Alfa Aesar, Aldrich, J&K, and GL Biochem Ltd., were used as received and were of analytical grade. If necessary, the reactions were conducted in dry solvents and under an argon atmosphere. ^{1}H NMR and ^{13}C NMR spectra were recorded on a Bruker AVANCE AV400 (400 MHz and 100 MHz). High resolution mass spectra (HRMS) were obtained on an IonSpec QFT mass spectrometer with ESI ionization. 3-(4,5-dimethylthiazol-2-yl) -2,5–diphenyltetrazolium bromide (MTT), ascorbic acid (AsA), 5'-GMP (purity ≥ 98.0%), DMEM (for 3T3 and HepG-2 cells) and RPMI1640 (for A549, A549R, Hela, LNCaP, MCF-7, PC3 and 293T cells) medium containing 10 % fetal bovine serum were purchased from GL Biochem Ltd. A Genomic DNA Mini Preparation Kit from Beyotime (China) was used for cellular drug uptake and DNA platination, and an Annexin V-FITC Apoptosis Detection Kit from KeyGEN Biotech (China) was used for Annexin V/PI coupled flow cytometric analysis. Phosphate-buffered saline (PBS) contains 137 mM NaCl, 2.7 mM KCl, 10 mM Na_2HPO_4 and 2 mM KH_2PO_4. Fetal bovine serum (FBS), 0.25% trypsin/EDTA solution, and penicillin-streptomycin solution were purchased from Invitrogen (Grand Island, NY, USA). A549R cells were maintained with 2 μg/mL cisplatin. HPLC analyses were performed on a Waters E2695−2998 system equipped with a Venusil MP C18 column (150 × 4.6 mm, 5 μm). HPLC profiles were recorded with a UV detector at 273 nm at room temperature. The mobile phase consisted of MeOH, and H_2O was used, at a flow rate of 1 mL/min. Reactions involved in the preparation of platinum compounds were conducted in the dark. HeLa (cervical carcinoma cell line), MCF-7 (breast gland adenocarcinoma), LNCaP and PC3 (prostatic cancer), HepG-2 (hepatocellular carcinoma), A549 and A549R (lung carcinoma) were used for the MTT assay.

BALB/c-nu mice (28-42 d) were purchased from the Laboratory Animal Center, Academy of Military Medical Science (Beijing, China). All of the animals received care in compliance with the guidelines outlined in the Guide for the Care and Use of Laboratory Animals.

Synthetic procedures

Scheme for 5b

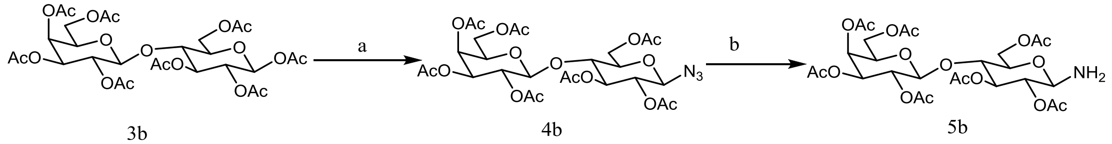

3b 4b 5b

Scheme 1. Synthetic route of **5b**. Conditions and reagents: (a) $TMSN_3$, BF_3OEt_2, and DCM; (b) Pd/C, H_2, and MeOH.

Preparation of 4b

Boron trifluoride etherate (94.32 mmol) was added to a solution of **3b** (27.76 mmol) and $TMSN_3$ (27.65 mmol) in dry CH_2Cl_2. The reaction mixture was stirred in the dark under a nitrogen atmosphere. TLC analysis was performed using ethyl acetate/hexane (1:1, v/v). CH_2Cl_2 (200 mL) was added, the reaction mixture was neutralized by adding saturated sodium bicarbonate solution (200 mL), and the resulting solution was washed with deionized water. The combined organic layers were dried over magnesium sulfate and were filtered and concentrated to dryness under reduced pressure. The resulting oil was then purified using column chromatography on silica gel. The relevant fractions were collected, combined and concentrated to dryness under reduced pressure to yield **4b** (41%): ^{1}H NMR (400 MHz, $CDCl_3$) δ 6.23 (s, 1H), 5.44 (t, *J* = 9.6 Hz, 1H), 5.34 (s, 1H), 5.08 (dd, *J* = 20.6, 12.7 Hz, 1H), 5.02 – 4.88 (m, 2H), 4.57 – 4.33 (m, 2H), 4.17 – 4.02 (m, 4H), 3.82 (m, 2H), 2.21 – 2.09 (m, 10H), 2.04 (s, 10H), 1.99 (s, 1H); and ^{13}C NMR (400 MHz, $CDCl_3$) δ 170.47, 170.25, 170.18, 170.04, 169.91, 169.42, 169.06, 101.25, 89.05, 75.84, 71.06, 70.83, 70.78, 69.75, 69.49, 69.27, 66.70, 61.56, 60.89, 21.03, 20.97, 20.93, 20.78, 20.74, 20.59, 14.28.

Preparation of 5b

A solution of **4b** in dry methanol containing 10% palladium on charcoal was exposed to hydrogen at room temperature in hydrogen. The azido group was reduced with Pd/C in the presence of hydrogen ballon. TLC analysis of the reaction mixture was performed, using ethyl acetate/hexane (1:1, v/v) as the solvent system, and it showed that the reaction had completed. The catalyst was filtered off through Celitew and was washed with methanol. The filtrate was concentrated to yield **5b** as a yellow oil (85%), which was not purified further.

Scheme for 8b

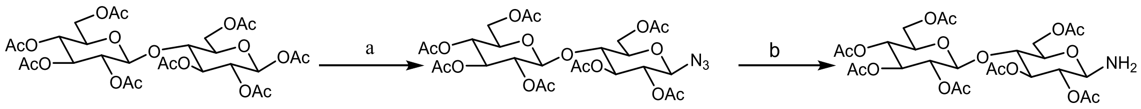

6b 7b 8b

Scheme 2. Synthetic route of **8b**. Conditions and reagents: (a) $TMSN_3$, BF_3OEt_2, and DCM; (b) Pd/C, H_2, and MeOH.

Preparation of 7b

Compound **7b** (45%) was prepared according to the procedure described for compound **4b**, starting from maltose. ^{1}H NMR (400 MHz, $CDCl_3$) δ 5.71 (d, *J* = 3.9 Hz, 1H), 5.65 (t, *J* = 10.0 Hz, 1H), 5.56 (t, *J* = 8.9 Hz, 1H), 5.35 (t, *J* = 9.9 Hz, 1H), 5.15 (dd, *J* = 10.6, 4.0 Hz, 1H), 5.08 (t, *J* = 8.9 Hz, 1H), 5.00 (d, *J* = 8.7 Hz, 1H), 4.85 – 4.75 (m, 1H), 4.60 – 4.49 (m, 2H), 4.41 (q, *J* = 7.1 Hz, 1H), 4.37 – 4.28 (m, 2H), 4.08 (d, *J* = 9.6 Hz, 1H), 2.53 – 2.27 (m, 21H); and ^{13}C NMR (400 MHz, $CDCl_3$) δ 170.67, 170.56, 170.25, 170.08, 169.64, 169.56, 95.85, 87.61, 75.23, 74.39, 72.48, 71.56, 70.12, 69.39, 68.67, 68.08, 62.67, 61.54, 20.99, 20.92, 20.89, 20.83, 20.70.

Preparation of 8b

Compound **8b** (80%) was prepared according to the procedure described for compound **5b**.

Scheme for 12b

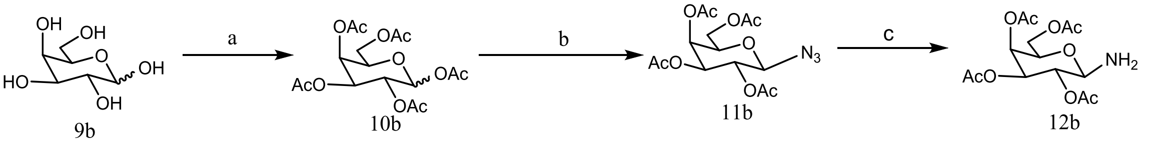

Scheme 3. Synthetic route of **12b**. Conditions and reagents: (a) Ac_2O and NaOAc; (b) $TMSN_3$, BF_3OEt_2, and DCM; (c) Pd/C, H_2, and MeOH.

Preparation of 10b

A mixture of **9b** 25 g and sodium acetate 15 g in 82 mL of acetic anhydride was stirred at 80 ℃ for 8 h. Subsequently, the mixture was poured into ice water and extracted with dichloromethane. The combined organic layer was washed with saturated sodium bicarbonate solution, dried over anhydrous sodium sulfate and concentrated. The solid obtained was recrystallized with alcohol to afford pure compound **10b** as a white solid (43.3 g, 80%).

Preparation of 11b

Compound **11b** (41%) was prepared according to the procedure described for compound **4b**. ^{1}H NMR (400 MHz, $CDCl_3$) δ 5.41 (t, *J* = 7.0 Hz, 1H), 5.18 – 5.07 (m, 1H), 5.07 – 4.94 (m, 1H), 4.59 (d, *J* = 8.7 Hz, 1H), 4.13 (pd, *J* = 14.3, 4.9 Hz, 2H), 4.00 (t, *J* = 6.5 Hz, 1H), 2.25 – 1.81 (m, 12H); and ^{13}C NMR (400 MHz, $CDCl_3$) δ 170.46, 170.21, 170.08, 169.46, 88.36, 72.92, 70.79, 68.12, 66.93, 61.31, 20.75, 20.71, 20.68, 20.61.

Preparation of 12b

Compound **12b** (80%) was prepared according to the procedure described for compound **5b**.

Scheme for 16b

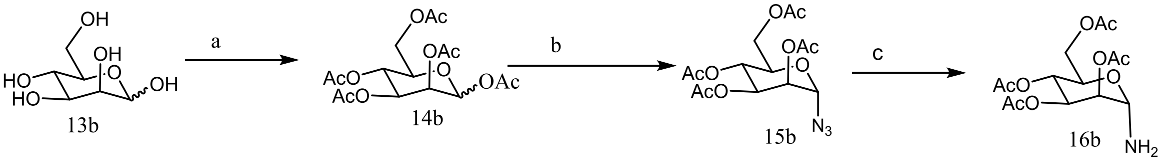

Scheme 4. Synthetic route of **16b**. Conditions and reagents: (a) Ac_2O and NaOAc; (b) $TMSN_3$, BF_3OEt_2, and DCM; (c) Pd/C, H_2, and MeOH.

Preparation of 15b

Compound **15b** (40%) was prepared according to the procedure described for compound **4b**. ^{1}H NMR (400 MHz, $CDCl_3$) δ 5.74 – 4.95 (m, 4H), 4.34 (dd, *J* = 119.4, 47.0 Hz, 3H), 2.05 (dd, *J* = 39.5, 25.8 Hz, 12H); and ^{13}C NMR (400 MHz, $CDCl_3$) δ 170.66, 169.93, 169.82, 169.70, 87.52, 70.68, 69.22, 68.29, 65.65, 62.19, 20.88, 20.77, 20.74, d 20.68.

Preparation of 16b

Compound **16b** (80%) was prepared according to the procedure described for compound **5b**.

Scheme for 24b

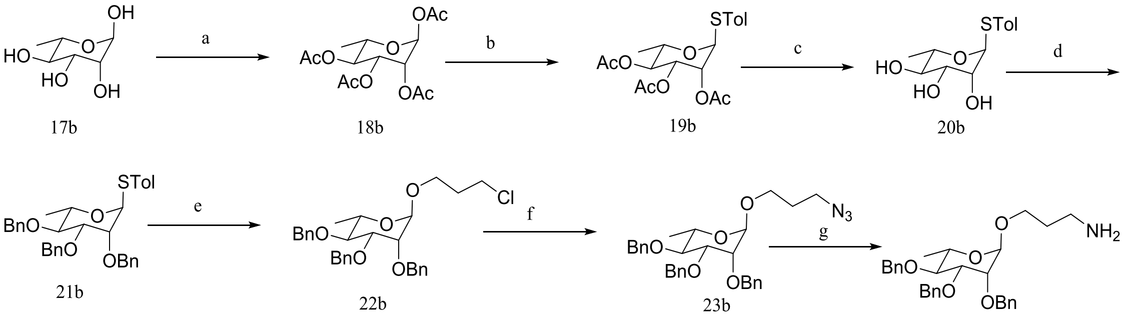

17b 18b 19b 20b

21b 22b 23b 24b

S

cheme 5. Synthetic route of **24b**. Conditions and reagents: (a) Ac_2O and NaOAc; (b) p-toluenethiol, BF_3OEt_2, and DCM; (c) MeONa and MeOH, rt, overnight; (d) BnBr, NaH, and DMF overnight; (e) TMSOTf and NIS; (f) NaN_3 and DMF overnight; (g) PPh_3, THF, and H_2O.

Preparation of 18b

Compound **18b** (85%) was prepared according to the procedure described for compound **10b**.

Preparation of 19b

To a mixture of **18b** (31.2 g, 80 mmol) and toluenethiol (11.8 g, 96 mmol) in anhydrous DCM, 200 mL of BF_3 Et_2O (35.2 mL, 280 mmol) were added at 0 ℃ dropwise. Subsequently, the mixture was reacted at room temperature overnight and was then poured into ice water ($NaHCO_3$) and extracted with dichloromethane. The combined organic layer was washed with saturated sodium bicarbonate solution, dried over anhydrous sodium sulfate and concentrated. Then, it was purified by column chromatography to yield the desired compound **19b** as a yellow solid (yield: 24.4 g, 67%).

Preparation of 20b and 21b

To a mixture of **19b** (24.4 g, 53.7 mmol) dissolved in anhydrous MeOH, 130 mL of MeONa (3.48 g, 64.4 mmol) were added and stirred for another 12 h to obtain the product **20b** as a slight yellow solid (14.7 g, 96%). To a solution of **20b** (17.3 g, 60.4 mmol) in anhydrous DMF 100 mL, NaH (8.6 g, 360 mmol) was added at 0 ℃ and stirred for another 20 min. BnBr (34.4 mL, 290 mmol) was added, and the mixture was reacted for another 12 h. The product 21b was finally purified by column chromatography as a yellow solid with a yield of 78% (30.4 g).

Preparation of 22b

A solution of **21b** (5 g, 7.7 mmol), NIS (2.61 g, 11.6 mmol), hydroxybutyric acid methyl ester (1.37 g, 11.6 mmol) and a 4A molecular sieve in anhydrous acetonitrile 20 mL was stirred at -49 ℃ for 20 min. TMSOTf (0.42 mL, 2.3 mmol) was added and reacted for another 2 h. The product 20b was finally purified by column chromatography as a slight yellow liquid with a yield of 23% (1.1 g).

Preparation of 23b

A solution of **22b** (0.50 mmol) in anhydrous DMF was treated with sodium azide (3.05 mmol), and the reaction mixture was stirred at 70 ℃. TLC analysis, using ethyl acetate/hexane (2:1, v/v), showed that the reaction had completed. The reaction mixture was concentrated to dryness under reduced pressure, dissolved in CH_2Cl_2 and then washed with deionized water. The combined organic layers were dried over magnesium sulfate, filtered and concentrated to dryness under reduced pressure. The resulting oil was then purified using column chromatography on silica gel. The relevant fractions were collected, combined and concentrated to dryness under reduced pressure to yield **23b** (73%).

Preparation of 24b

A solution of **23b** (2.33 g, 1 equiv) and PPh_3(2.4 g, 2 equiv) in THF 33 mL and H_2O 11 mL was stirred at room temperature overnight. The product **24b** was finally purified by column chromatography as a white solid with a yield of 40%. ^{1}H NMR (400 MHz, MeOD) δ 7.38 (dd, *J* = 56.2, 28.8 Hz, 15H), 4.69 (ddd, *J* = 31.9, 28.2, 8.4 Hz, 7H), 4.05 – 3.05 (m, 7H), 2.71 (s, 2H), 1.73 (s, 2H), 1.27 (s, 3H); and ^{13}C NMR (400 MHz, MeOD) δ 138.57, 138.43, 138.33, 128.10, 128.00, 127.87, 127.84, 127.64, 127.51, 127.41, 127.39, 97.70, 80.10, 79.66, 75.04, 74.88, 72.48, 71.52, 67.87, 65.10, 38.32, 31.22, 17.19.

Scheme for 32b

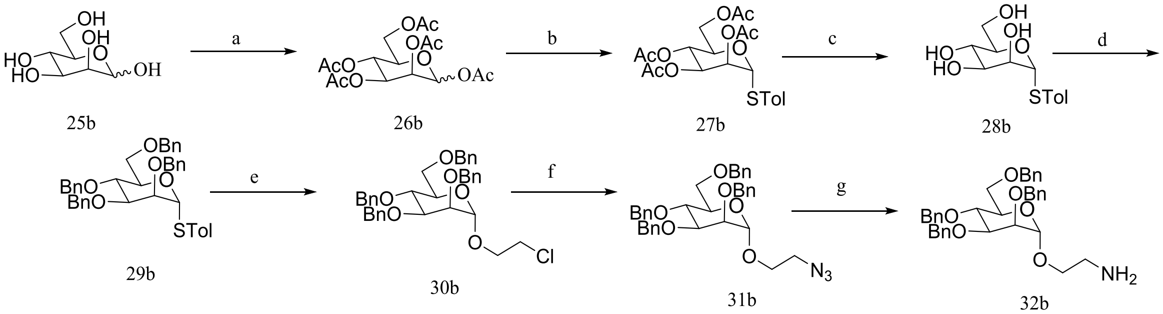

25b 26b 27b 28b 29b 30b 31b 32b

Scheme 6. Synthetic route of 32**b**. Conditions and reagents: (a) Ac_2O and NaOAc; (b) *p*-toluenethiol, BF_3OEt_2, and DCM; (c) MeONa and MeOH rt overnight; (d) BnBr, NaH, and DMF overnight; (e) TMSOTf and NIS; (f) NaN_3 and DMF overnight; (g) PPh_3, THF, and H_2O.

Preparation of 32b

Compound **32b** (35%) was prepared according to the procedure described for compound **24b**. ^{1}H NMR (400 MHz, methanol-d4) δ 7.73 – 6.87 (m, 20H), 5.01 – 4.59 (m, 7H), 4.59 – 4.35 (m, 4H), 3.91 (dd, *J* = 18.5, 9.1 Hz, 2H), 3.76 – 3.56 (m, 2H), 3.44 (dt, *J* = 10.3, 5.3 Hz, 1H), 2.79 (t, *J* = 5.0 Hz, 2H); and ^{13}C NMR (400 MHz, methanol-d4) δ 137.95, 137.88, 137.68, 127.52, 127.44, 127.28, 127.26, 127.20, 127.07, 126.88, 126.82, 97.57, 79.22, 74.14, 72.54, 71.89, 71.38, 71.08, 68.39, 67.51, 39.96.

Scheme for 38b

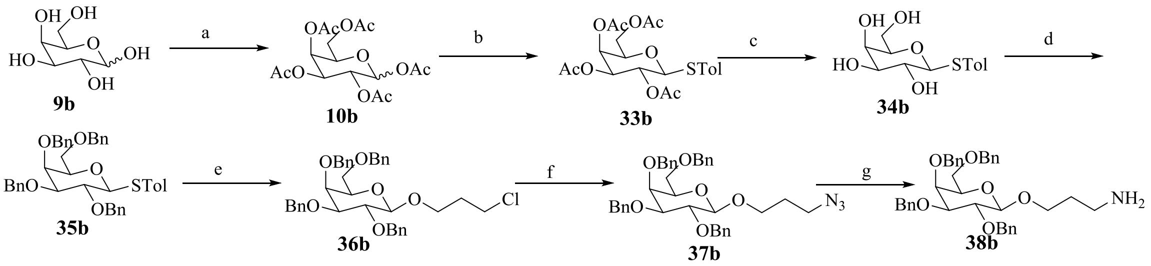

Scheme 7. Synthetic route of 38**b**. Conditions and reagents: (a) Ac_2O and NaOAc; (b) *p*-toluenethiol, BF_3OEt_2, and DCM; (c) MeONa and MeOH rt overnight; (d) BnBr, NaH, and DMF overnight; (e) TMSOTf and NIS; (f) NaN_3 and DMF overnight; (g) PPh_3, THF, and H_2O.

Preparation of 38b

Compound **38b** (38%) was prepared according to the procedure described for compound **24b**. ^{1}H NMR (400 MHz, methanol-d4) δ 8.08 – 6.54 (m, 20H), 4.90 – 4.29 (m, 8H), 4.16 – 3.13 (m, 8H), 2.80 (d, J = 6.4 Hz, 2H), 1.84 (d, J = 43.1 Hz, 3H); and ^{13}C NMR (400 MHz, methanol-d4) δ 164.89, 140.25, 139.99, 139.48, 129.59, 129.53, 129.49, 129.42, 129.19, 129.13, 128.98, 128.86, 128.81, 128.72, 105.18, 83.40, 80.75, 76.21, 75.40, 74.53, 73.89, 72.11, 70.04, 42.37, 37.07, 31.61.

Scheme for 45b

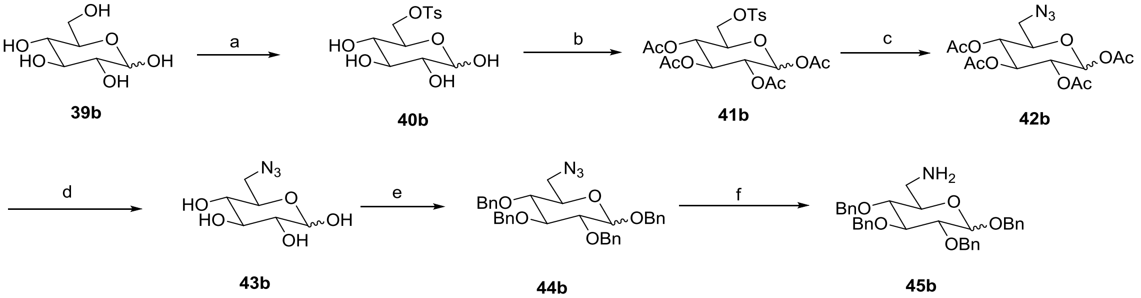

Scheme 8. Synthetic route of **45b**. Conditions and reagents: (a) TsCl and Py; (b) Ac_2O and Py; (c) NaN_3 and DMF overnight; (d) MeONa and MeOH rt overnight; (e) BnBr, NaH, and DMF overnight; (f) PPh_3, THF, and H_2O.

Preparation of 45b

Compound **45b** (38%) was prepared according to the procedure described for compound **24b**. ^{1}H NMR (400 MHz, methanol-d4) δ 7.89 – 6.80 (m, 20H), 5.05 – 4.37 (m, 10H), 4.12 – 3.34 (m, 3H), 3.06 – 2.39 (m, 2H); and ^{13}C NMR (400 MHz, methanol-d4) δ 140.12, 139.97, 139.86, 139.64, 139.58, 139.54, 139.15, 138.88, 138.05, 129.47, 129.41, 129.32, 129.28, 129.14, 129.09, 129.00, 128.94, 128.88, 128.84, 128.61, 103.95, 96.45, 85.91, 83.61, 83.07, 81.54, 80.37, 80.28, 76.81, 76.54, 76.49, 76.44, 75.86, 75.79, 75.69, 73.69, 72.90, 72.75, 72.40, 70.21, 43.69, 43.54.

Scheme for 48b

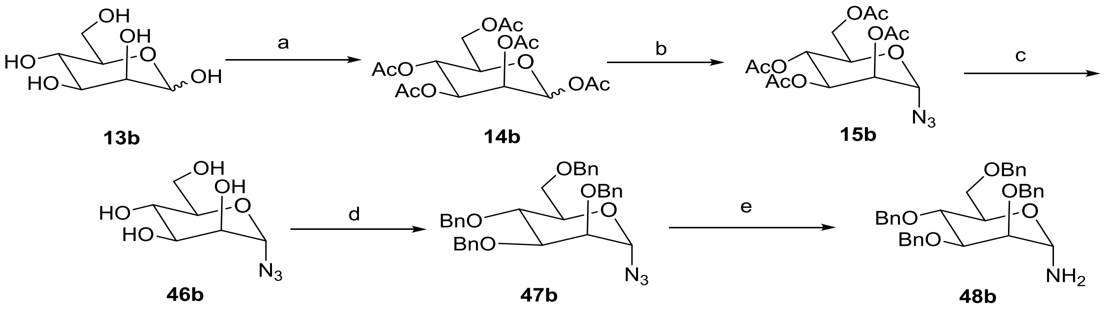

Scheme 9. Synthetic route of 38**b**. Conditions and reagents: (a) Ac_2O and NaOAc; (b) $TMSN_3$, BF_3OEt_2, and DCM; (c) MeONa and MeOH rt overnight; (d) BnBr, NaH, and DMF overnight; (e) PPh_3, THF, and H_2O.

Preparation of 46b and 47b

Compounds **46b** (38%) and **47b** (45%) were prepared according to the procedure described for compounds **20b** and **21b**, respectively.

Preparation of 48b

Compound **48b** (45%) was prepared according to the procedure described for compound **32b**. ^{1}H NMR (400 MHz, methanol-d4) δ 8.02 – 6.91 (m, 20H), 5.59 – 3.73 (m, 8H), 3.35 (dd, J = 58.5, 43.2 Hz, 2H), 1.19 (t, J = 103.5 Hz, 3H); and ^{13}C NMR (400 MHz, methanol-d4) δ 140.09, 139.97, 139.84, 139.77, 135.15, 135.04, 134.39, 134.30, 130.84, 130.71, 130.23, 130.11, 129.73, 129.61, 129.56, 129.49, 129.45, 129.38, 129.30, 129.26, 129.10, 129.03, 128.99, 128.89, 128.84, 128.78, 128.76, 128.69, 84.97, 80.08, 77.86, 77.08, 76.09, 75.70, 75.50, 74.55, 74.33, 73.82, 73.19, 72.89, 70.71, 70.44.

***In Vitro* Cellular Cytotoxicity Assays**

Cells seeded in 96-well plates were incubated in a 5% CO_2 atmosphere in 100 μL of complete medium at 37 ℃ for 24 h. Then, 100 μL of freshly prepared culture medium containing drugs at different concentrations was added and incubated for another 48 h. MTT (5 mg/mL, 20 μL) was added and incubated for 3 h. Finally, the medium was removed, and DMSO (150 μL) was added. The absorbance was measured at 570 nm using a Bio-Rad 680 microplate reader. The IC_{50} values were calculated using GraphPad Prism software. Technical repetitions and independent experiments were based on three parallel experiments.

For cytotoxicity assays using the GLUT1 inhibitor 4,6-O-ethylidene-α-D-glucose (EDG), phloretin and the OCT2 inhibitor Ctd., a similar procedure as described above was followed except that MCF-7 cells (3500 cells/well) were seeded on a 96 well plate in 100 μL of RPMI and incubated for 24 h at 37 ℃. EDG, phloretin and Ctd. containing RPMI medium were used for serial dilution of the concentrated solutions of the platinum compounds, and 100 μL/well were added (resulting in final inhibitor concentrations of 75 mM, 100 μM and 3 mM, respectively). The cytotoxicity profiles of the compounds were evaluated using the MTT assay.

Cellular Platinum Uptake and DNA Platination

The cellular uptake was measured in HeLa and MCF-7 cells. HeLa and MCF-7 cells were seeded in 6-well plates overnight and then were incubated with 30 μM and 20 μM drug under standard culture conditions for 10 h. Subsequently, the cells were washed with PBS buffer three times and harvested by trypsinization. The harvested cells were concentrated and digested by nitric acid for ICP-MS. The cell numbers were counted before digestion. A Genomic DNA Mini Preparation Kit was used for the isolation of DNA in HeLa cells, and Pt concentrations in the cellular DNA of HeLa cells digested by nitric acid were also measured by ICP-MS.

Biodistribution of 7d and Satraplatin in MCF-7 Bearing Animals

The *in vivo* biodistribution of **7d** and satraplatin in MCF-7 bearing animals was evaluated. **7d** and satraplatin were assessed *in vivo* using a mouse model of GLUT1 expressing breast cancer induced in Balb/c mice. Each treatment group consisted of 5 animals (n = 5). Intravenous bolus injections of 10 mg/kg of **7d** and satraplatin commenced when tumors reached ~50 mm^3 in size. The treatment groups included **7d** and satraplatin and a control group receiving PBS. Platinum complexes were freshly prepared by dissolution in PBS prior to injection. The biodistribution was assessed by ICP-MS at 9 h and 24 h post-administration (i.v.) in a mouse model bearing MCF-7 cancer cells overexpressing GLUT1. To investigate the GLUT1-mediated uptake, we used satraplatin, a non-targeted platinum complex, as a control. Quantification of the platinum concentration in organ tissue was measured by ICP-MS at 9 h and at 24 h following administration of **7d** and satraplatin after nitric acid digestion.

Computational details [1-11]

A Molecular Operating Environment (MOE 2015.10) was used for molecular docking. A crystal structure of GLUT1 was downloaded from the protein data bank (PDB ID: 4PYP). This structure was protonated in MOE 2015.10. The active site was defined with a 6 Å radius around the bound of 6 in the crystal structure. The triangle matcher algorithm of the MOE software package was selected to dock the identified hit compounds into the protein active site. The scoring function had to comply with the following parameters: (1) specification of ASE scoring to rank the pose output by the placement stage; (2) specification of force field refinement to relax the poses; and (3) specification of Affinity dG Scoring to rank the poses using the refinement stage. The free energy of binding was calculated from the contributions of the hydrophobic, ionic, and hydrogen bonds, van der Waals interactions between the protein and the ligand, intramolecular hydrogen bonds and the strains of the ligand. We observed that the docking poses were ranked by the binding free energy calculation in the S field.

Reduction of Pt(IV) complexes by Vc

To investigate the binding properties of DNA with Pt(II) complexes, 5'-GMP was selected as a model of DNA. The oxaliplatin was incubated with 5'-GMP at 37 ℃ for 24 h. The results revealed that new peaks of Oxp-Pt(II)-GMP (the conjugated complexes of oxaliplatin with 5'-GMP) were generated by the mixture, which demonstrated the potency of 5'-GMP to combine with Pt(II) complexes. Further experiments were

designed to test the reduction potential of Pt(IV) complexes. The results proved that the glycosylated platinum(IV) complexes could be reduced by Vc and could release Pt(II) complexes. Then, the Pt(II) compounds combined with 5'-GMP to form OxpPt(II)-GMP, as confirmed by HRMS.

NMR spectra of compounds.

^{1}H-NMR spectrum for compound 4b

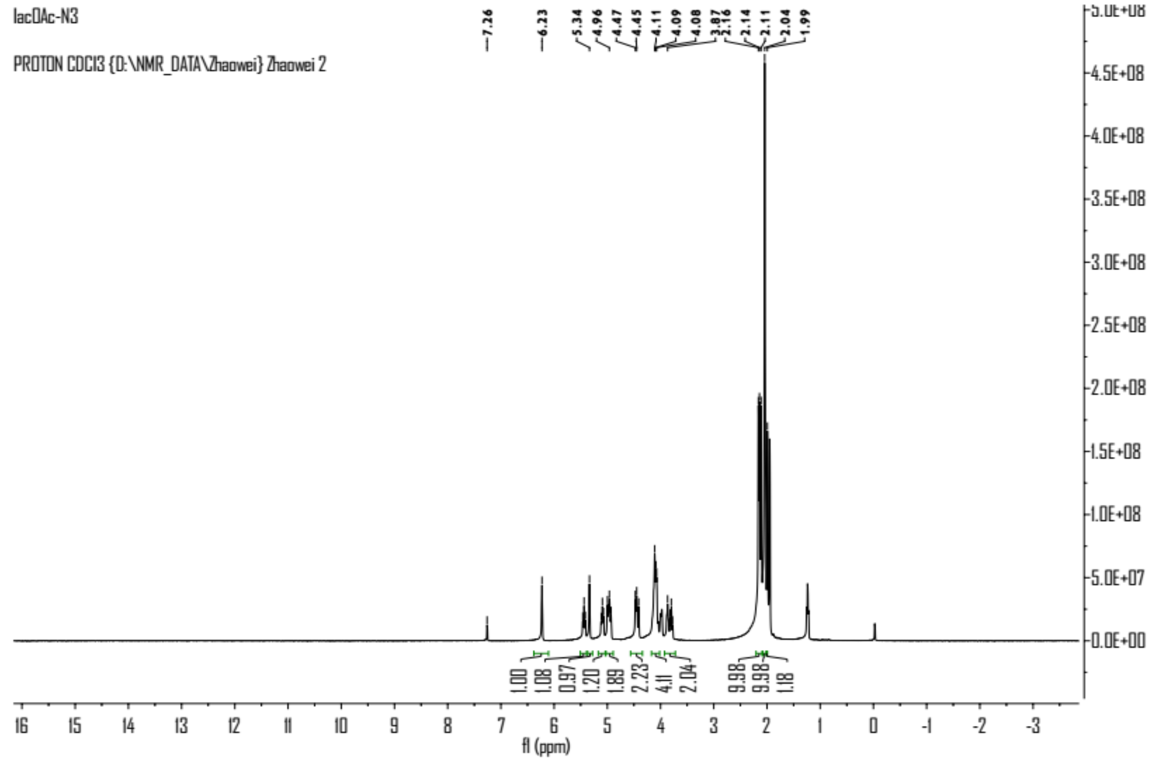

^{13}C NMR spectra for compound 4b

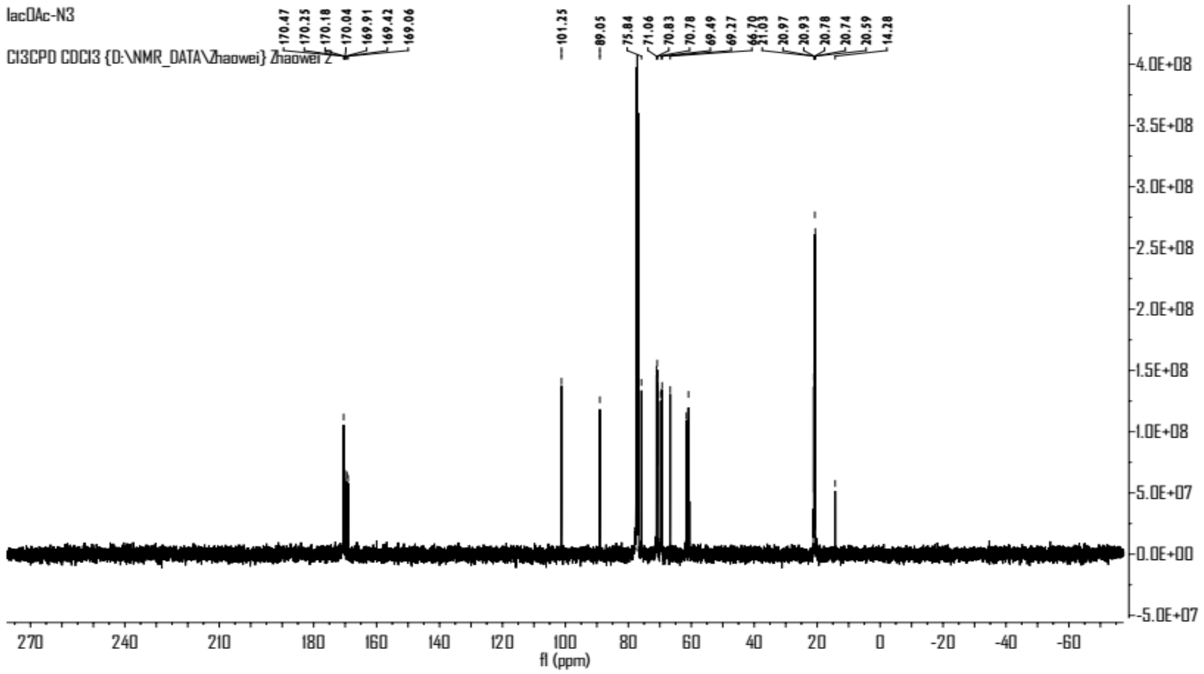

^{1}H-NMR spectrum for compound 7b

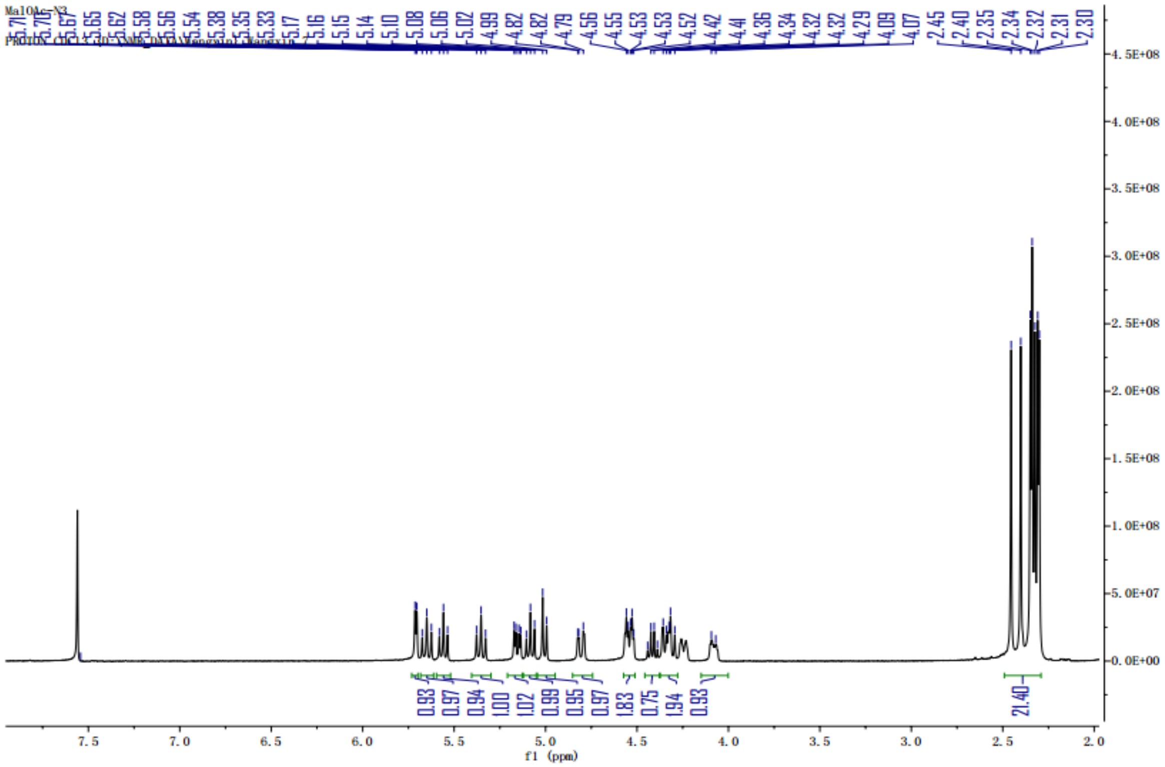

^{13}C NMR spectra for compound 7b

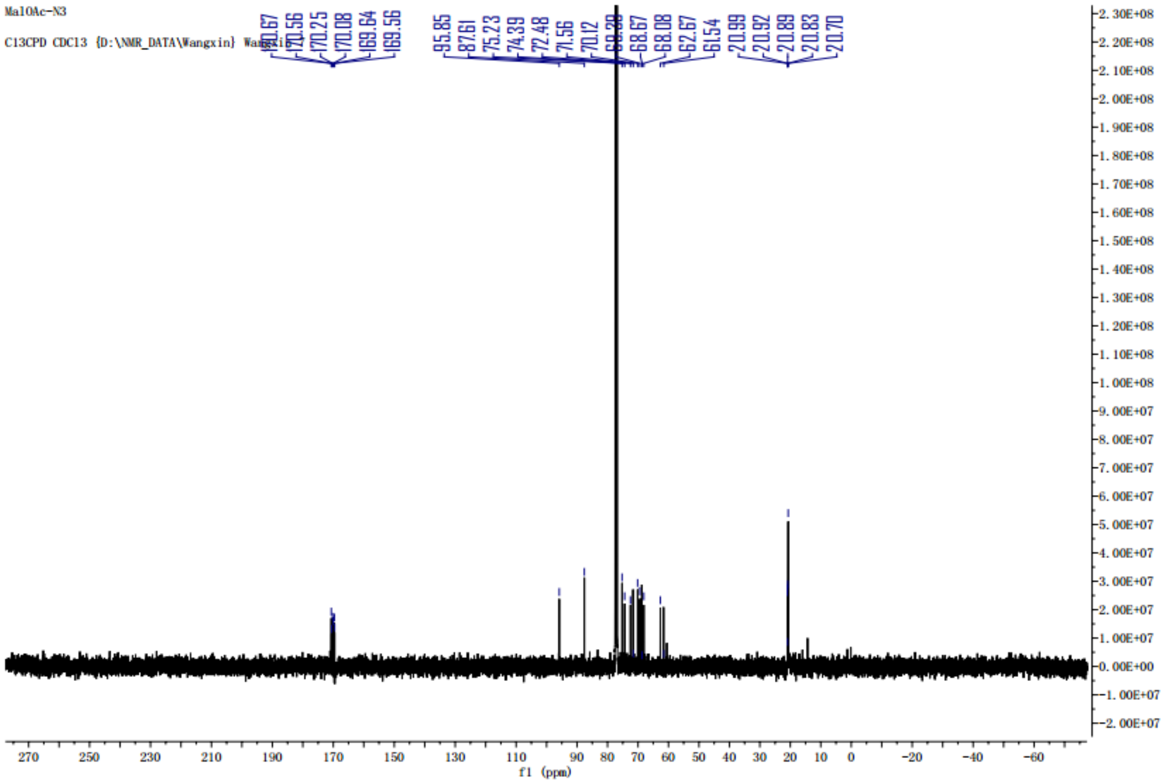

^{1}H-NMR spectrum for compound 11b

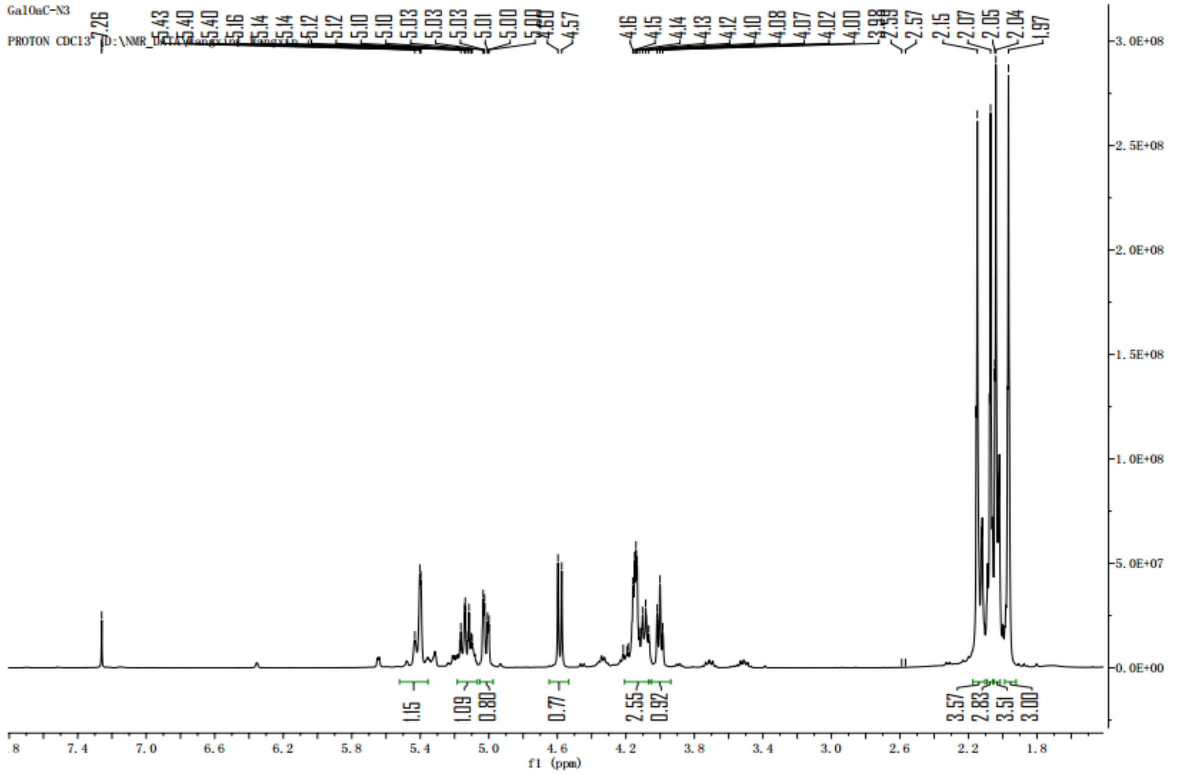

^{13}C NMR spectra for compound 11b

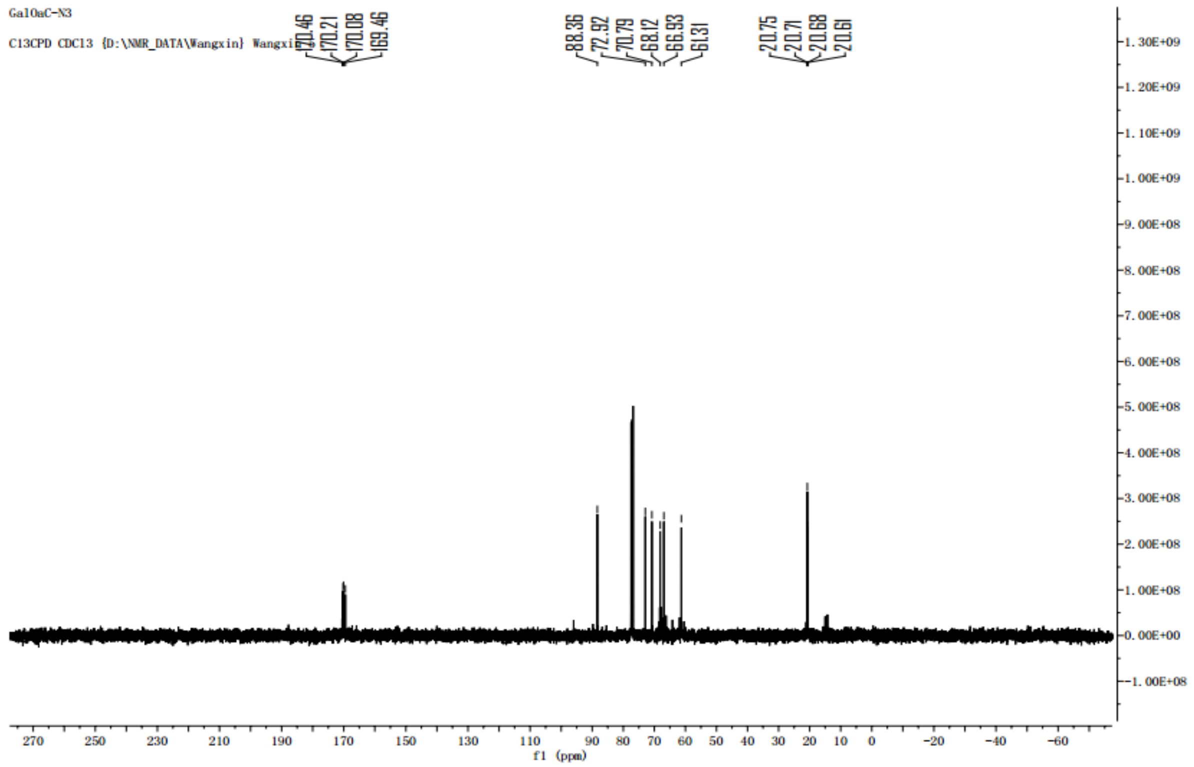

^{1}H-NMR spectrum for compound 15b

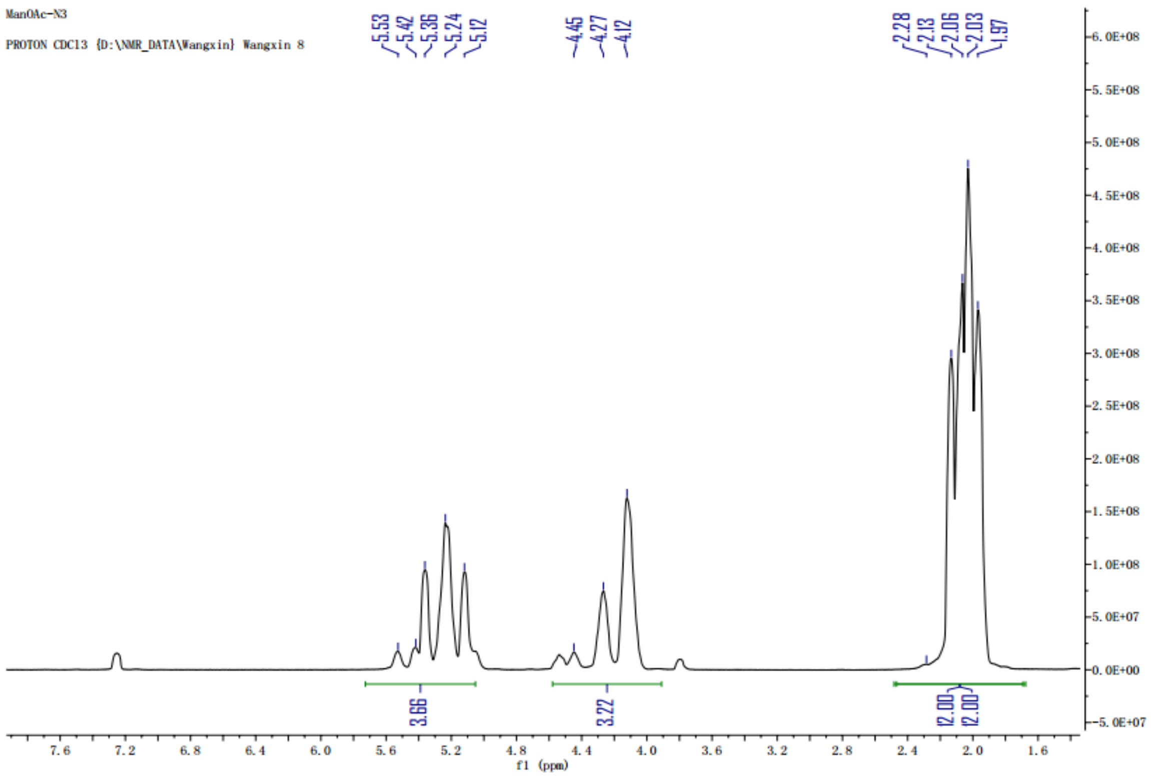

^{13}C NMR spectra for compound 15b

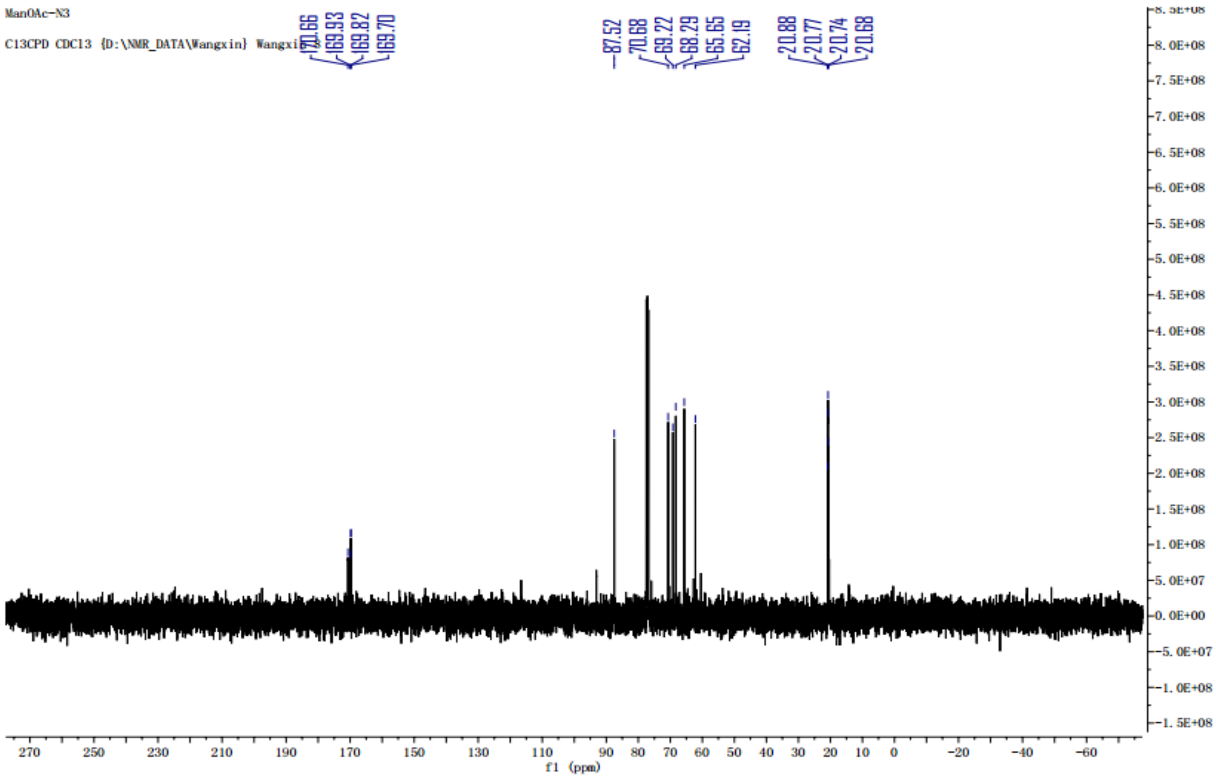

^{1}H-NMR spectrum for compound 24b

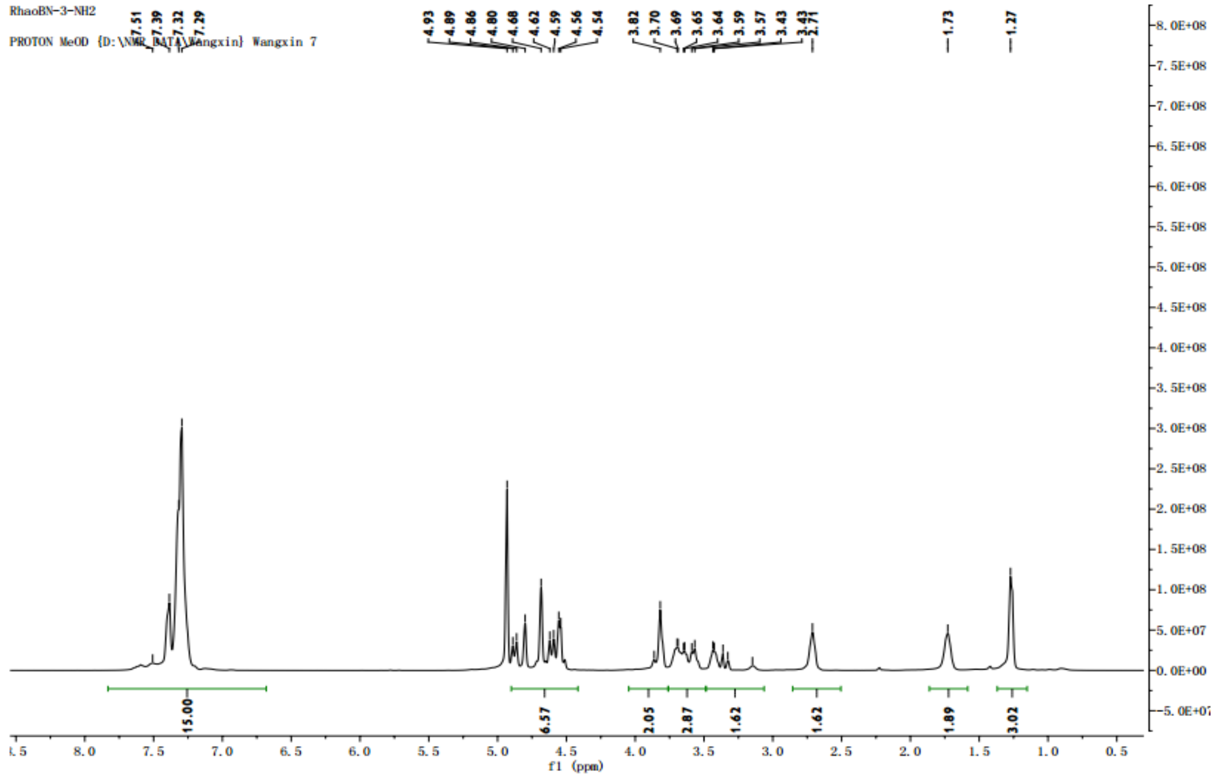

^{13}C NMR spectra for compound 24b

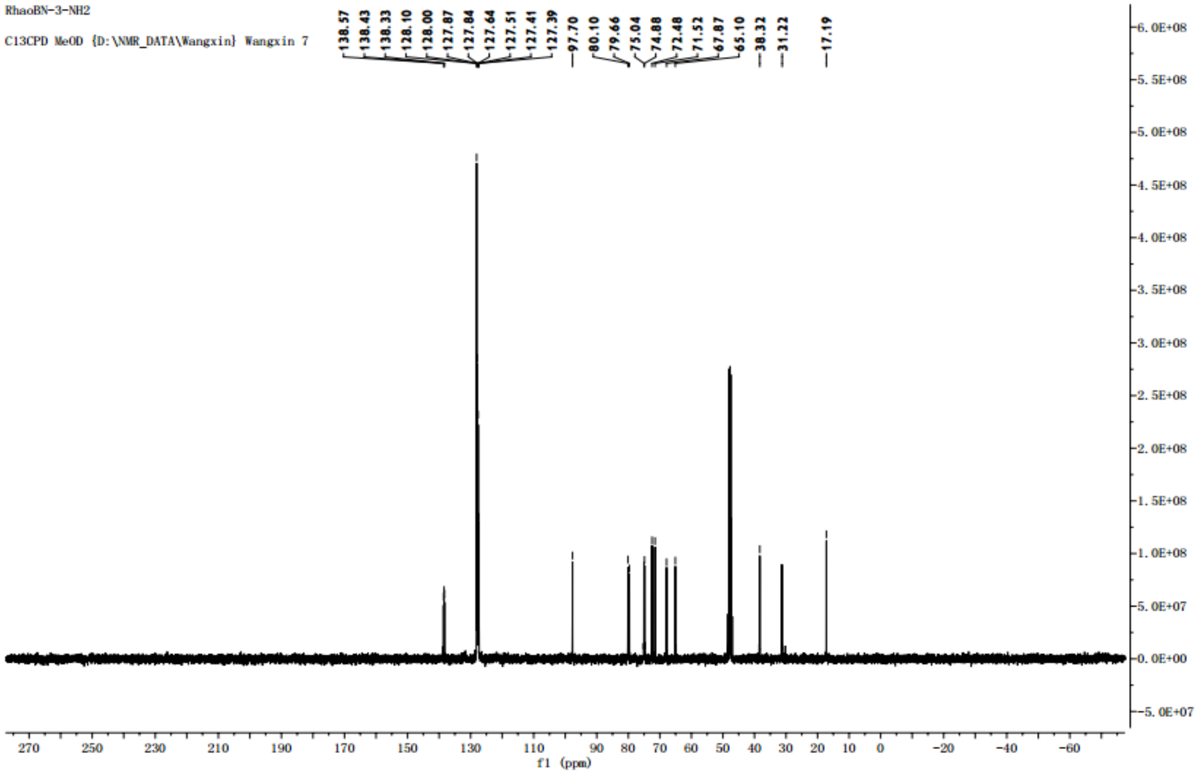

^{1}H-NMR spectrum for compound 32b

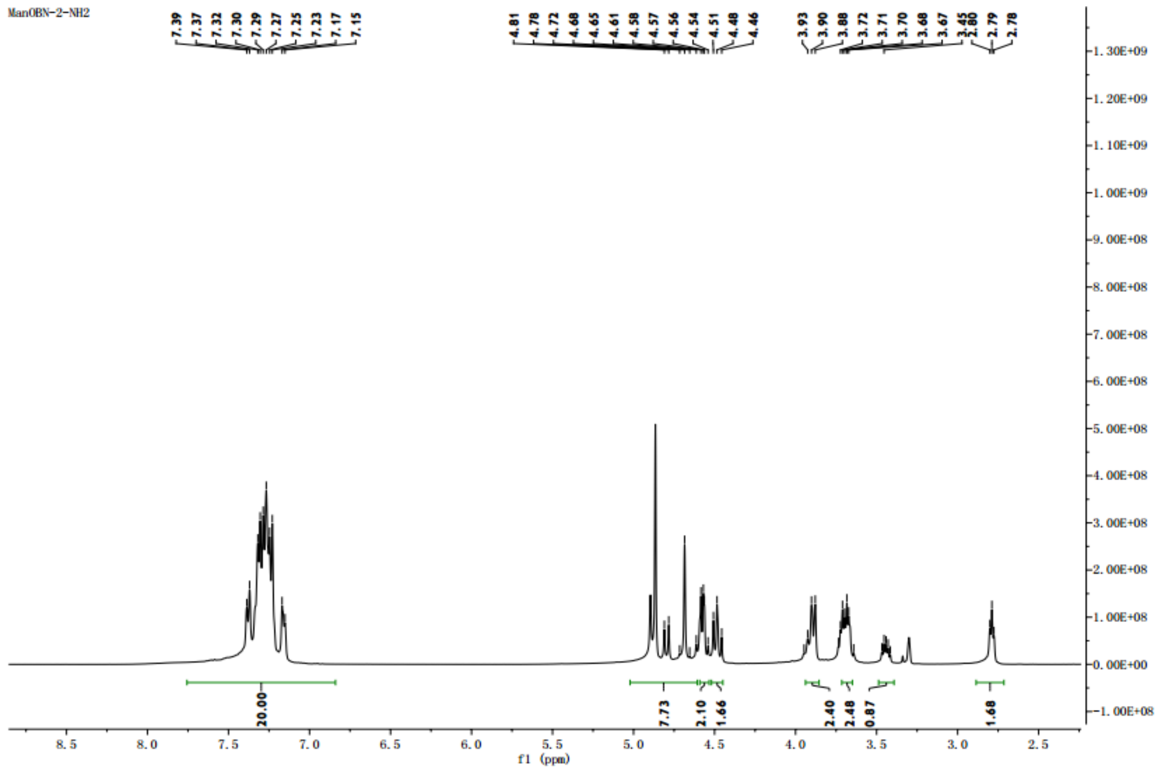

13C NMR spectra for compound 32b

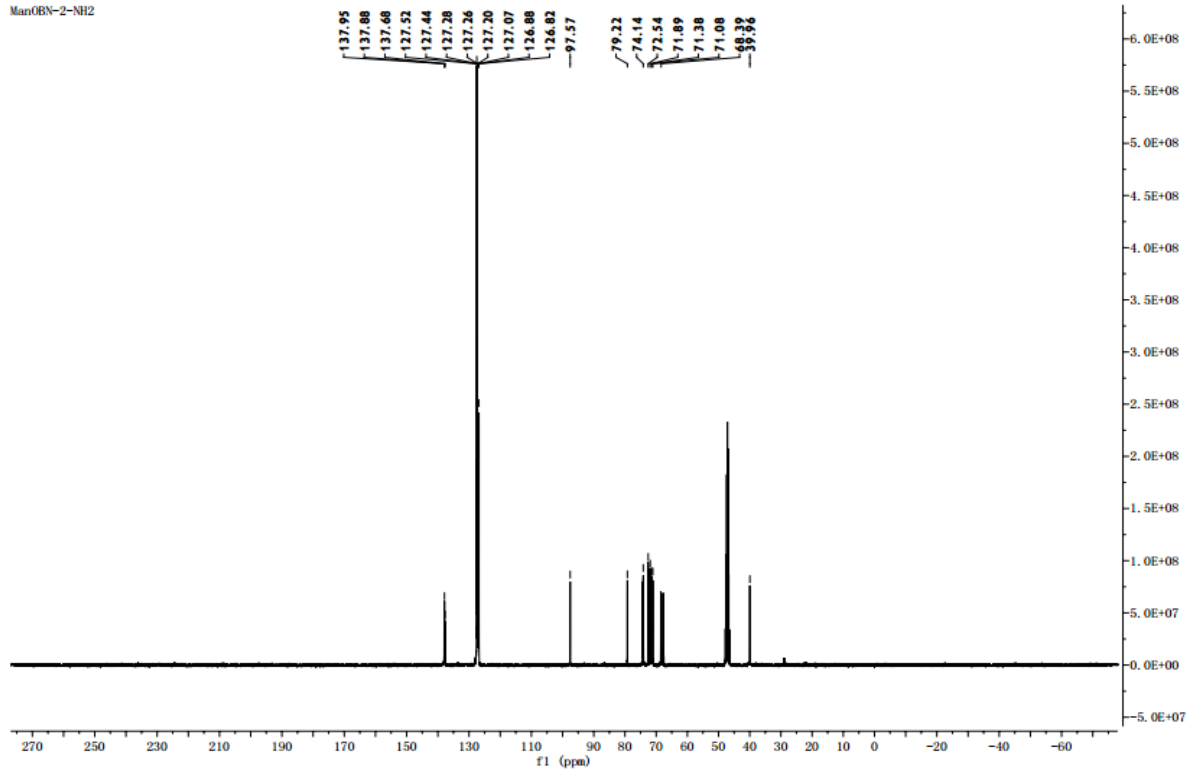

1H-NMR spectrum for compound 38b

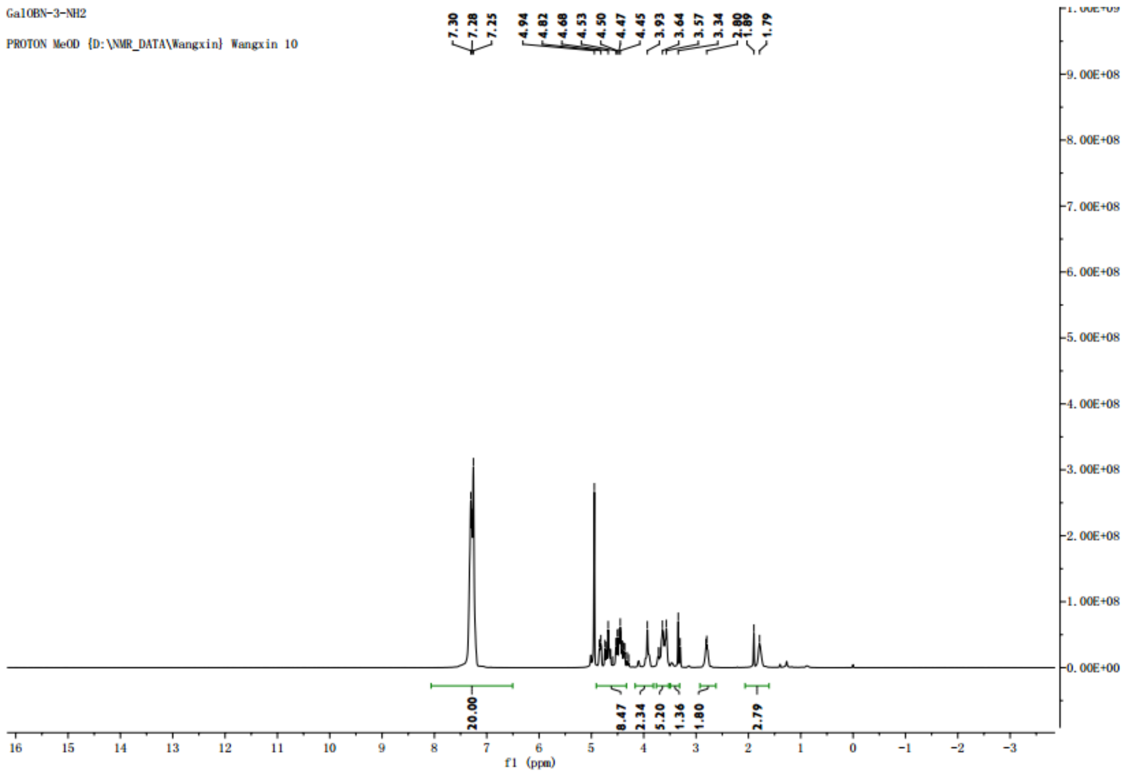

^{13}C NMR spectra for compound 38b

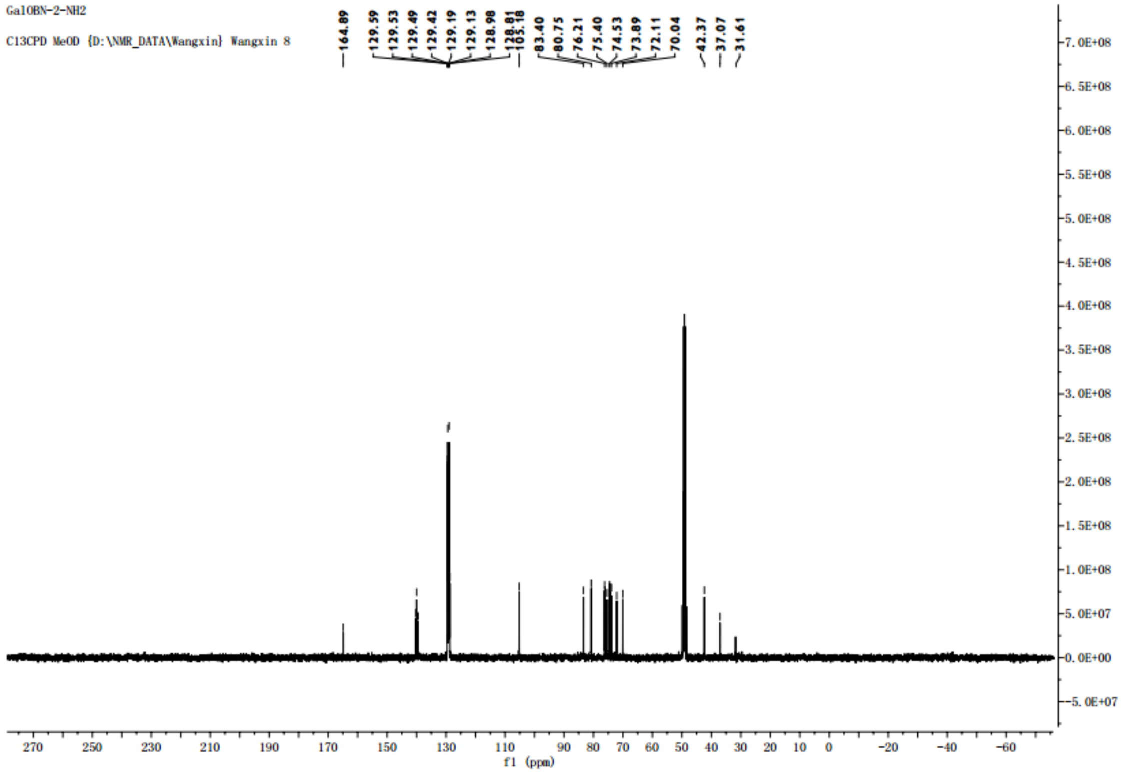

^{1}H-NMR spectrum for compound 45b

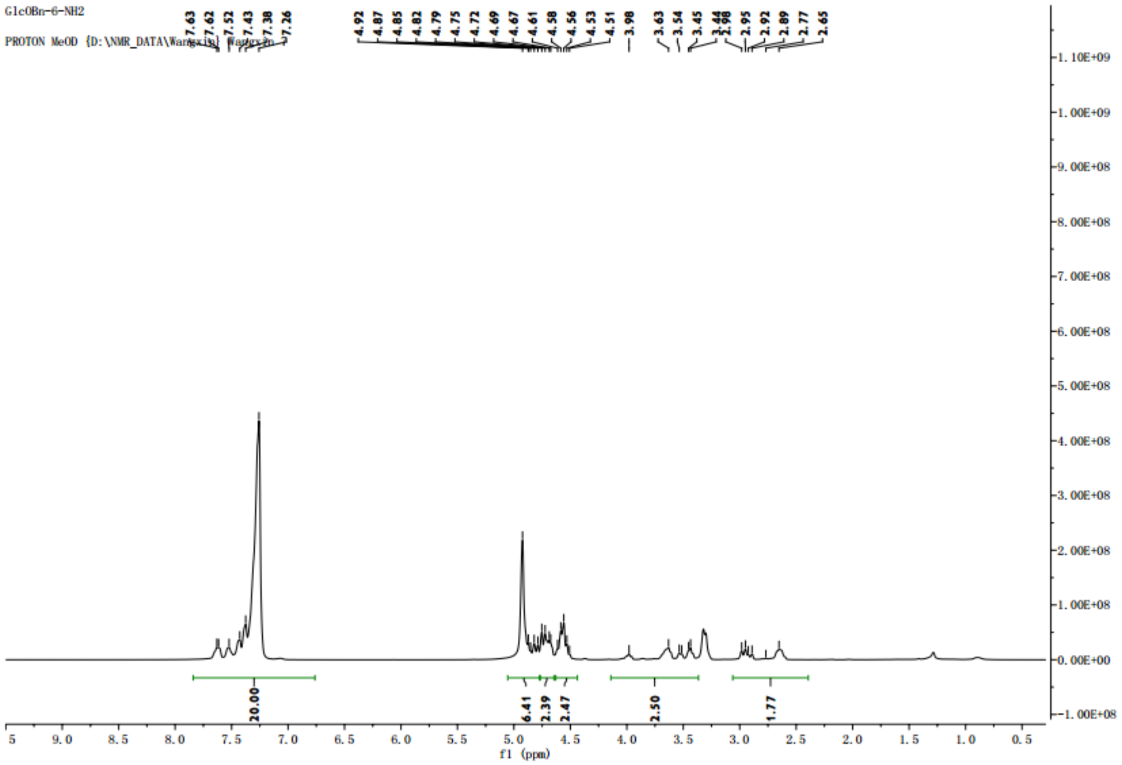

^{13}C NMR spectra for compound 45b

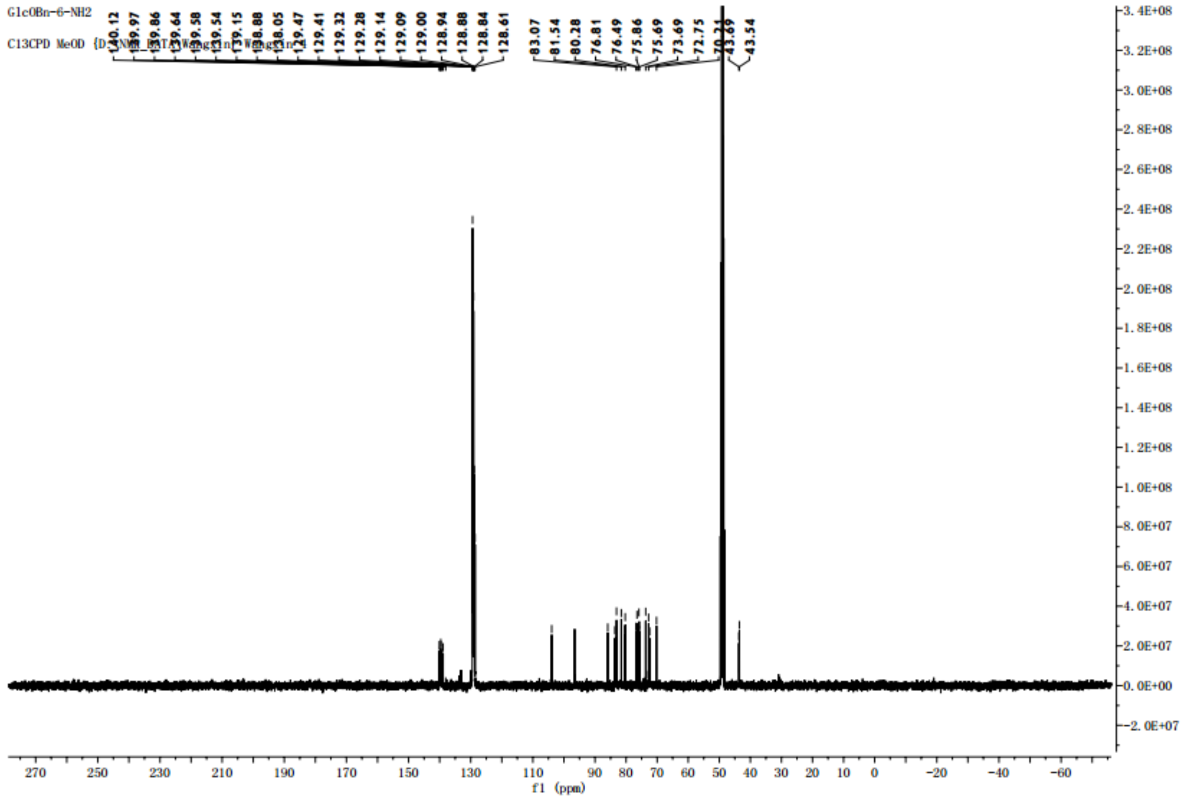

^{1}H-NMR spectrum for compound 48b

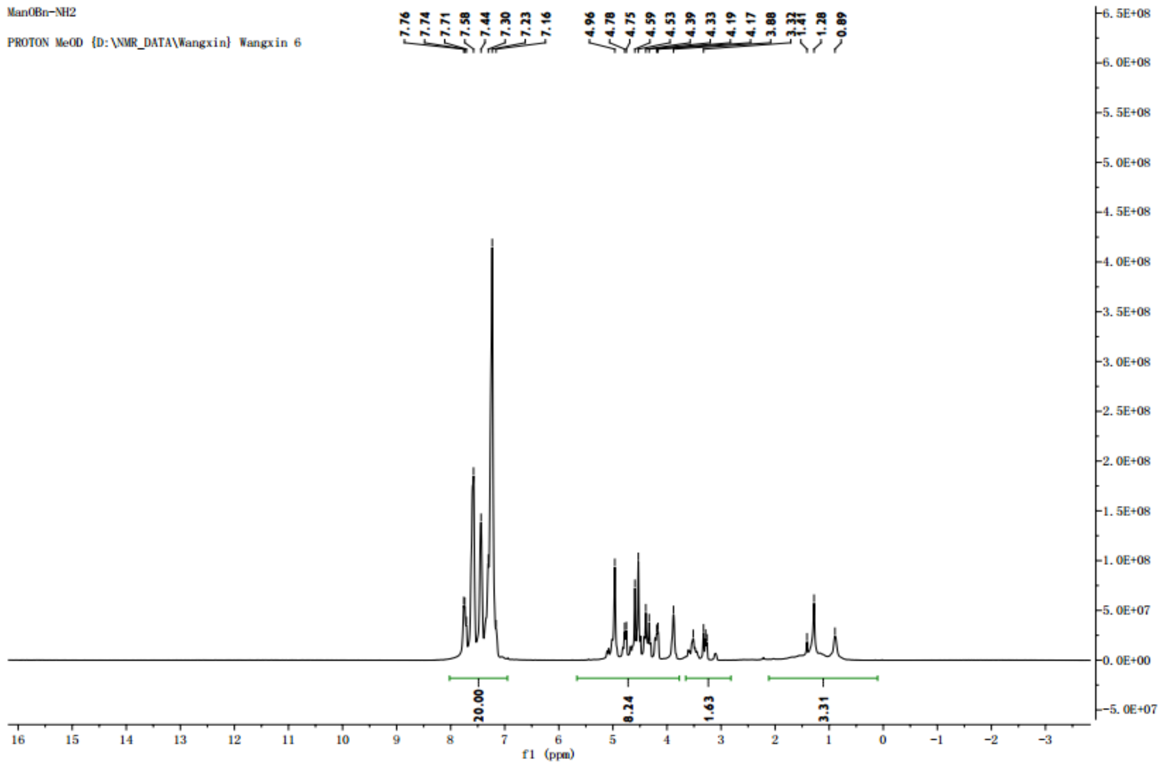

^{13}C NMR spectra for compound 48b

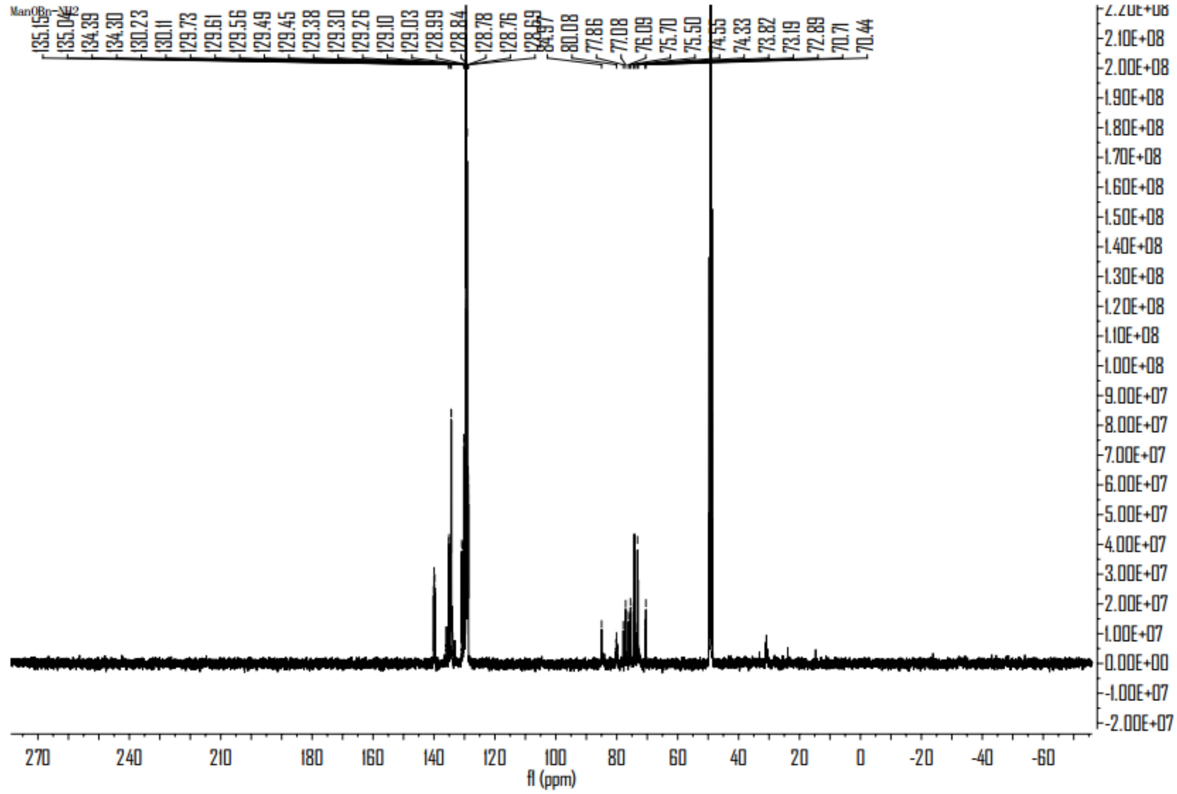

^{1}H-NMR spectrum for compound 1c

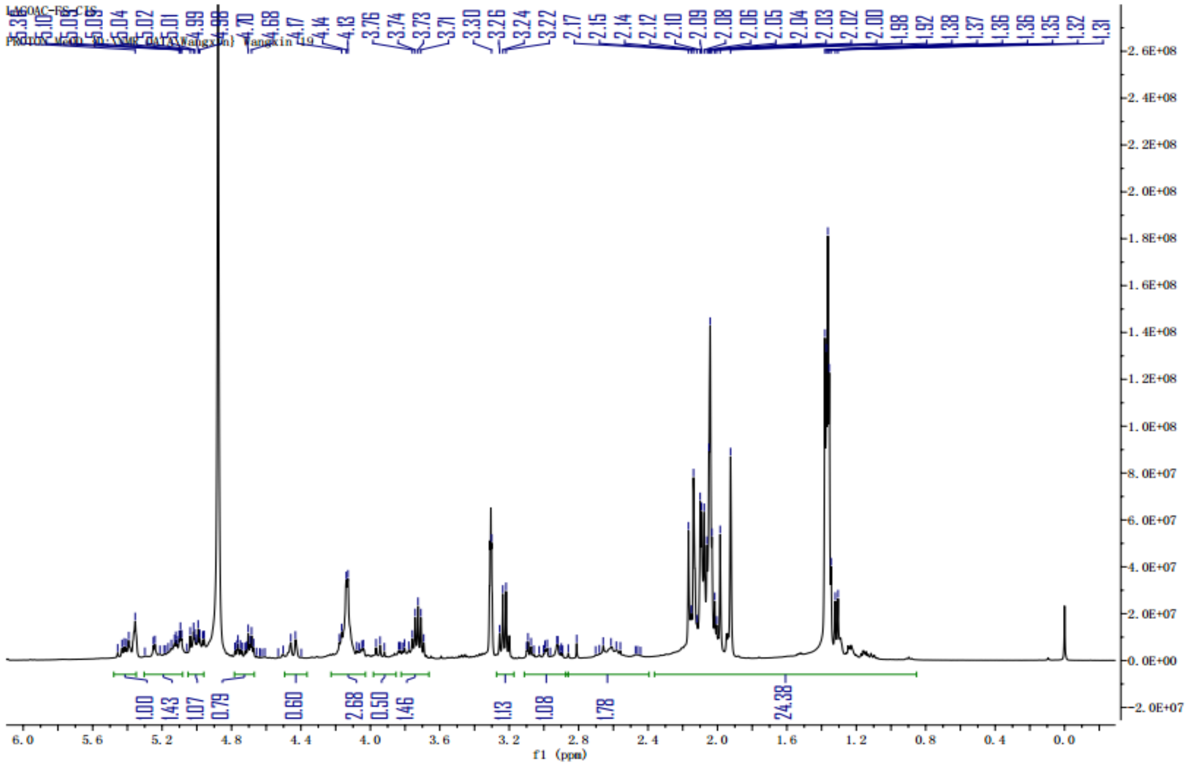

¹³C NMR spectra for compound 1c

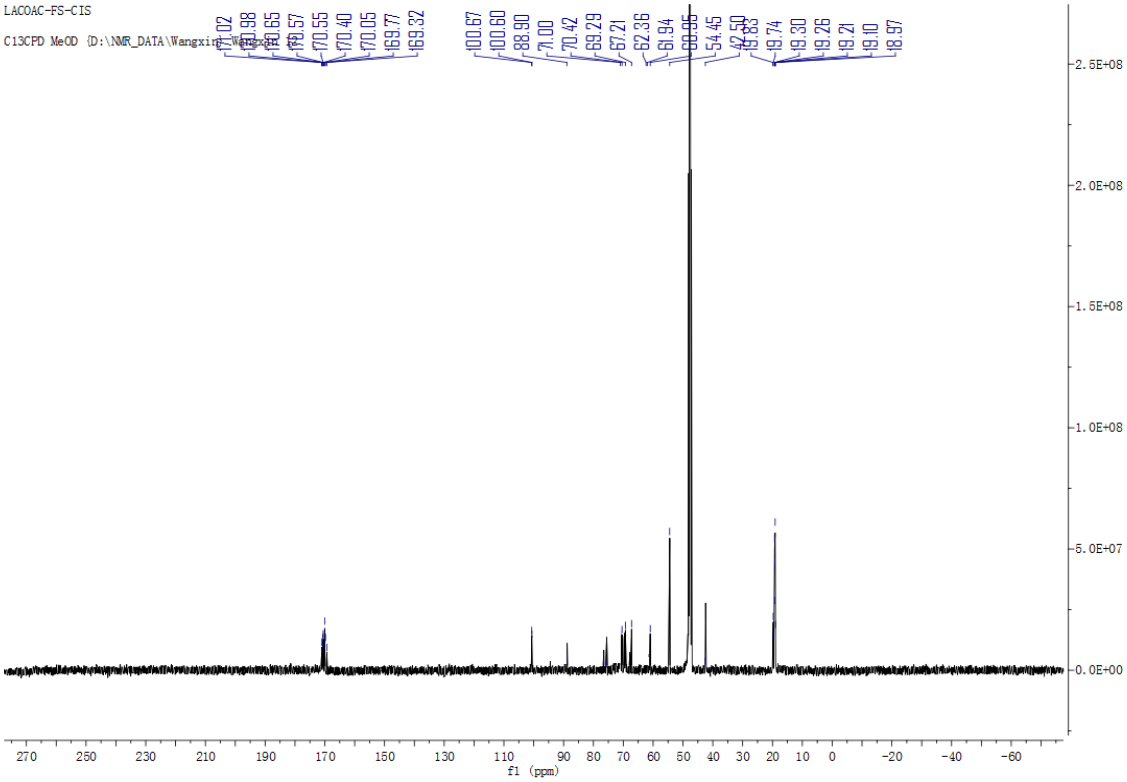

HRMS spectrum for compound 1c

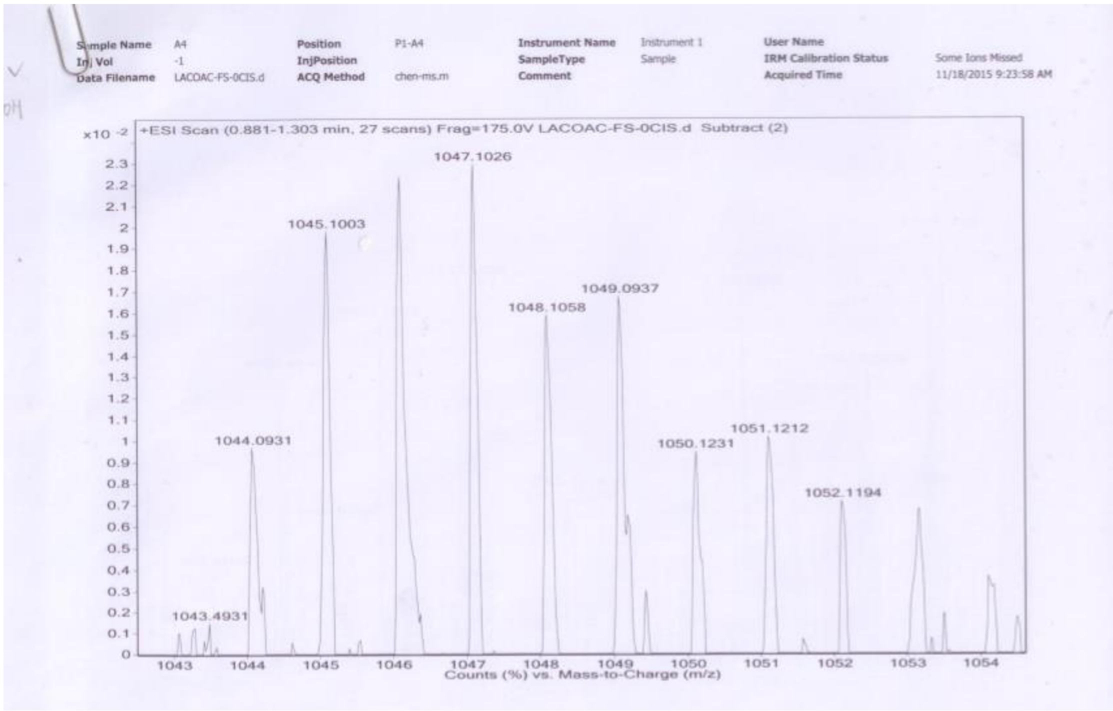

^{1}H-NMR spectrum for compound 2c

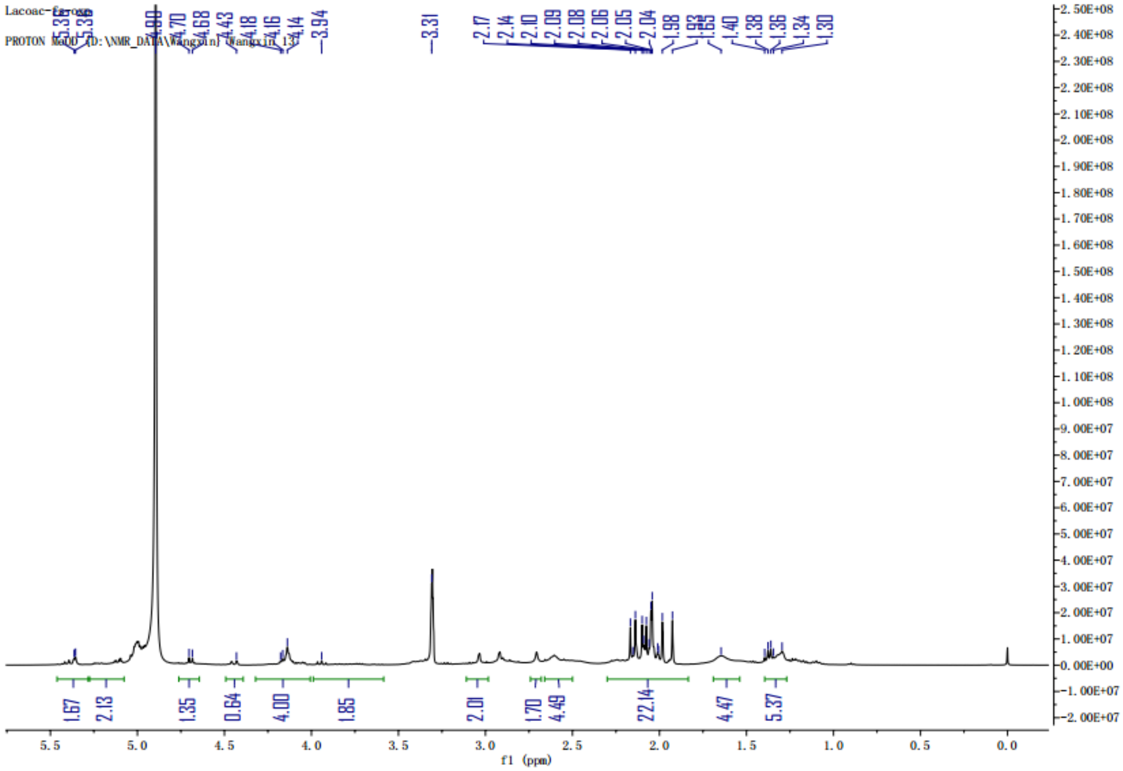

^{13}C NMR spectra for compound 2c

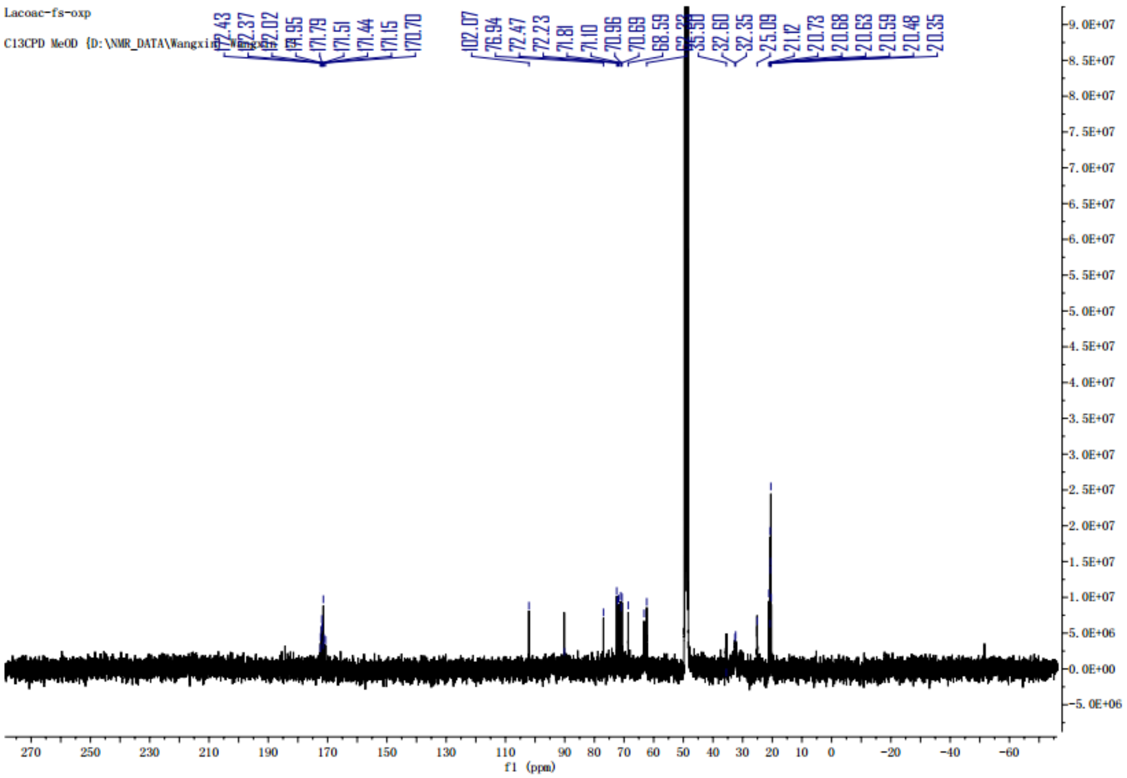

HRMS spectrum for compound 2c

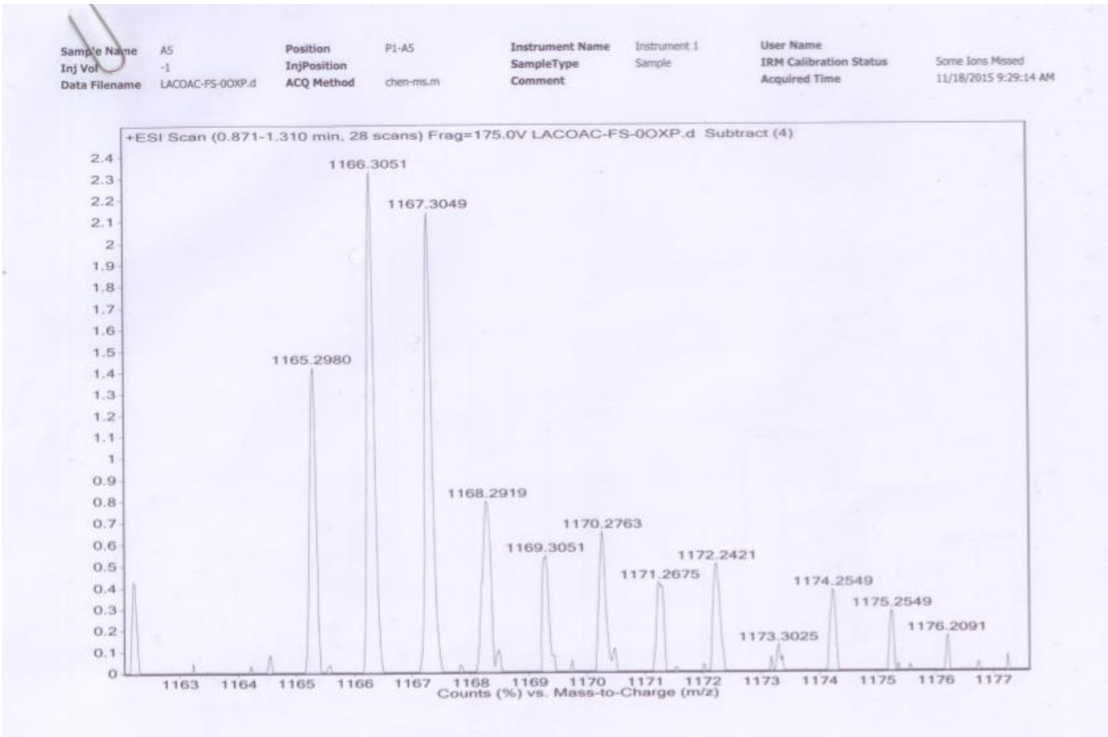

^{1}H-NMR spectrum for compound 3c

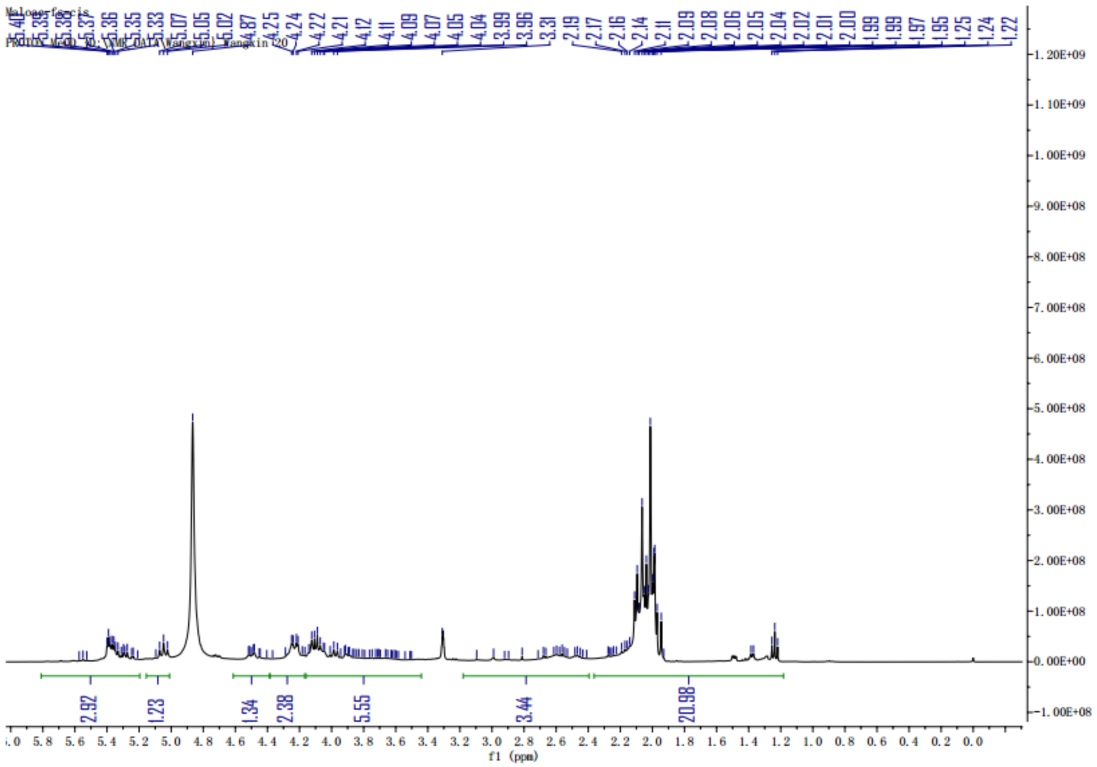

^{13}C NMR spectra for compound 3c

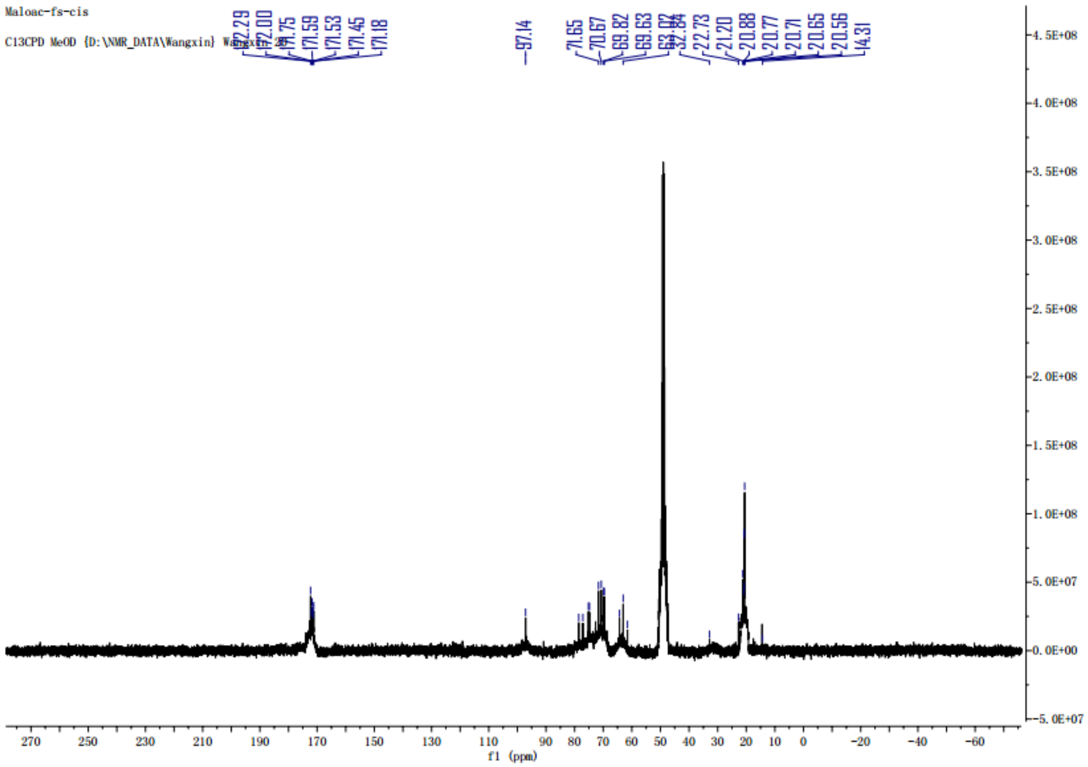

HRMS spectrum for compound 3c

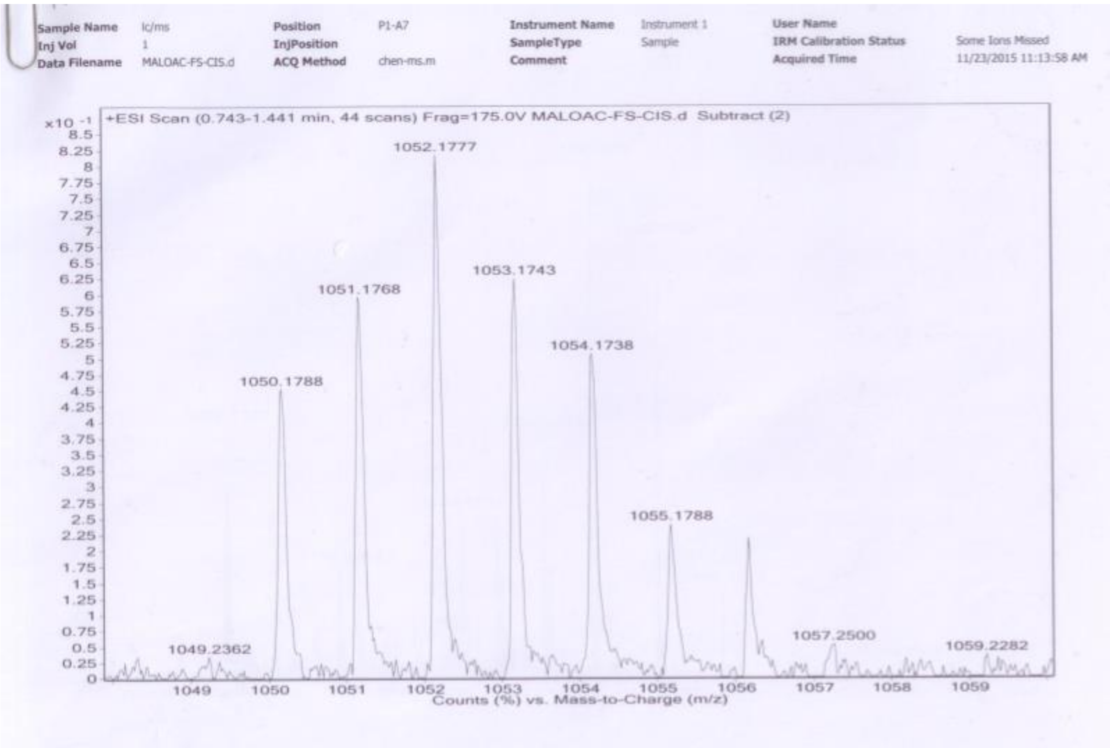

^{1}H-NMR spectrum for compound 4c

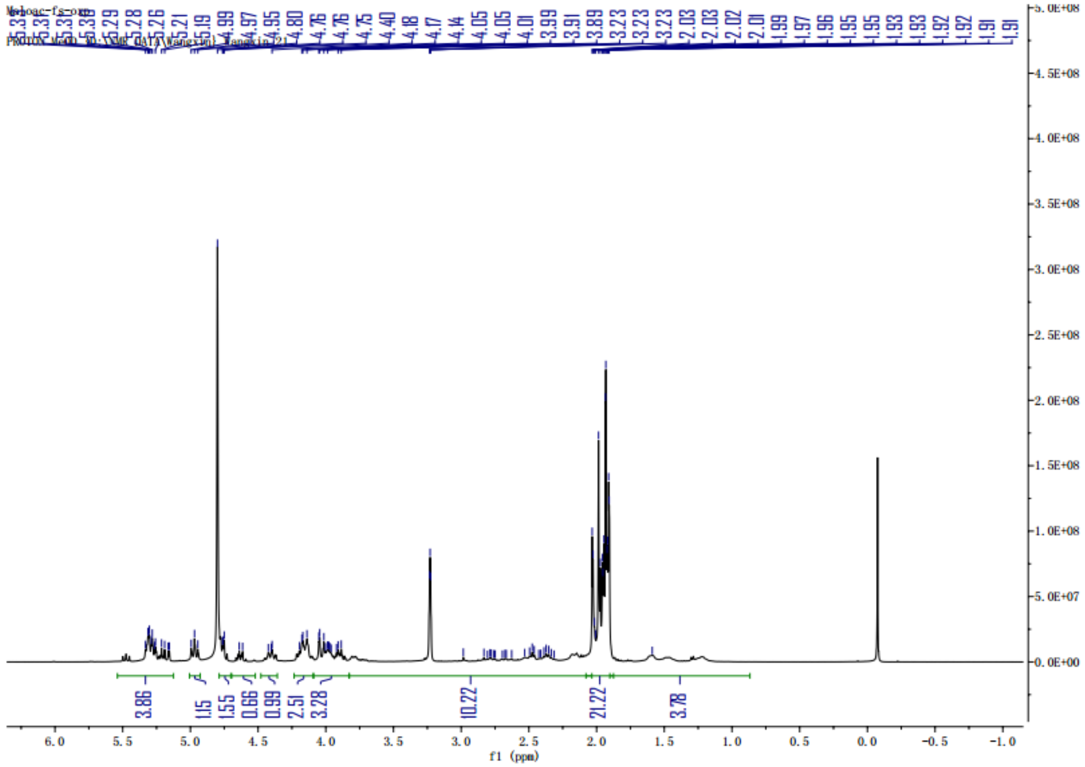

^{13}C NMR spectra for compound 4c

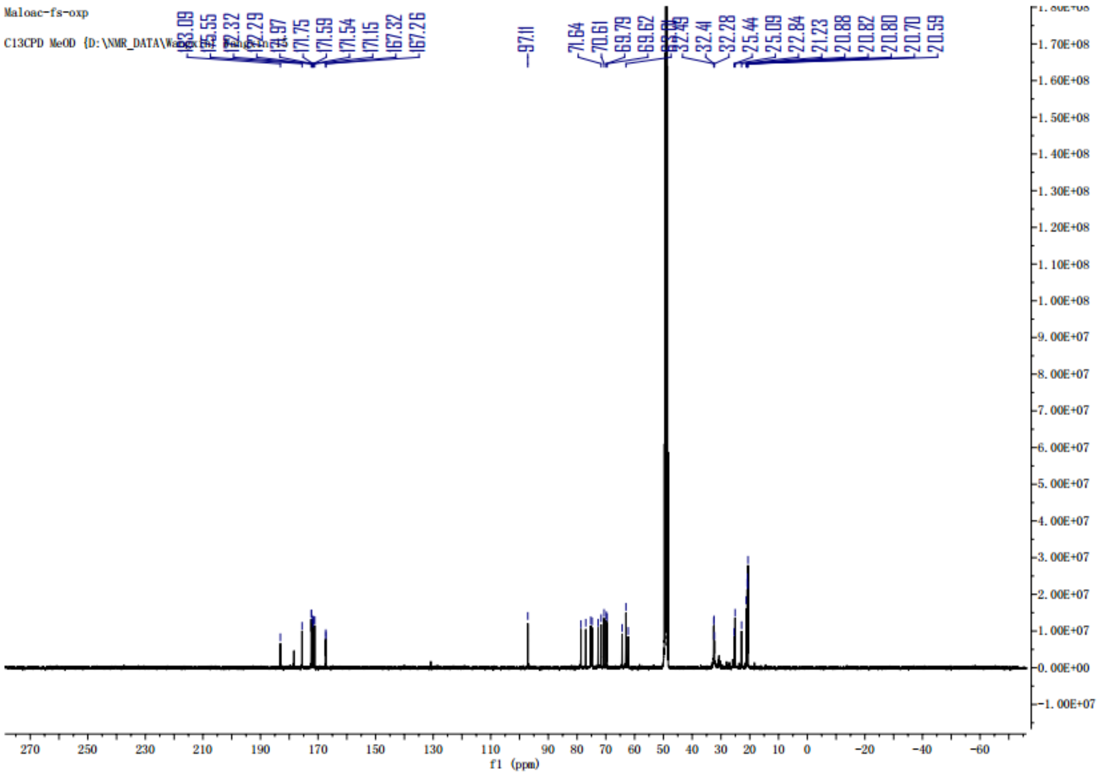

HRMS spectrum for compound 4c

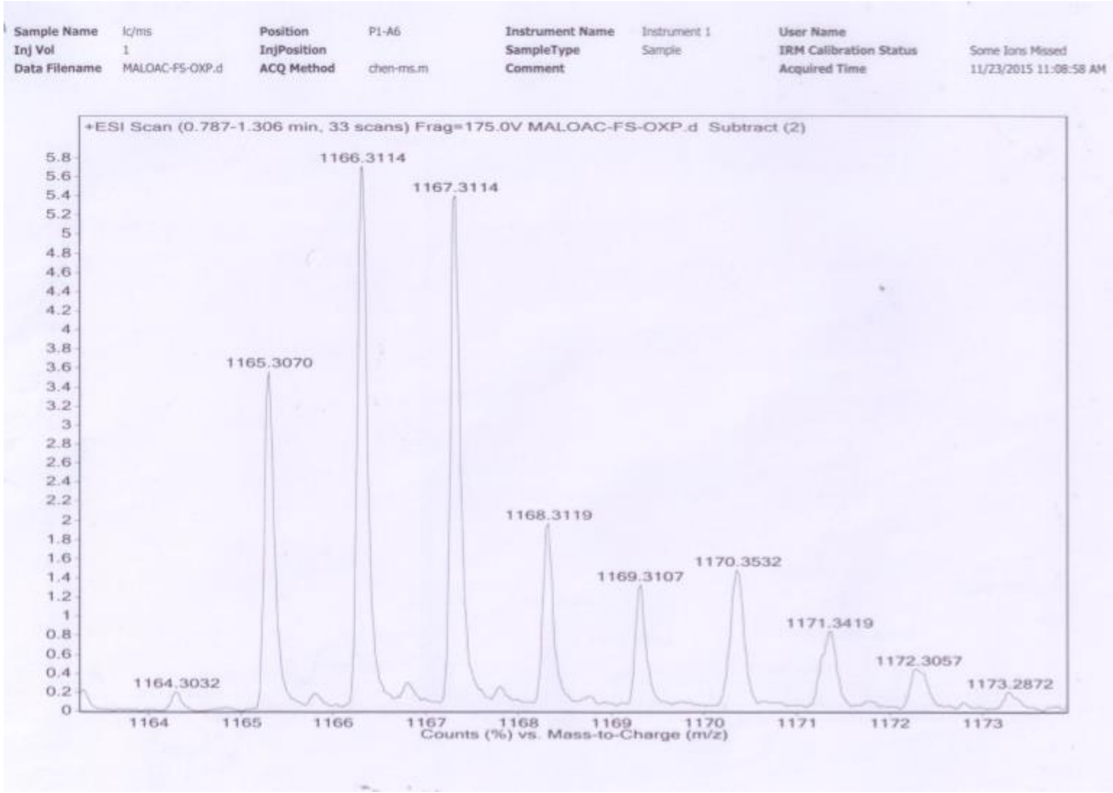

^{1}H-NMR spectrum for compound 5c

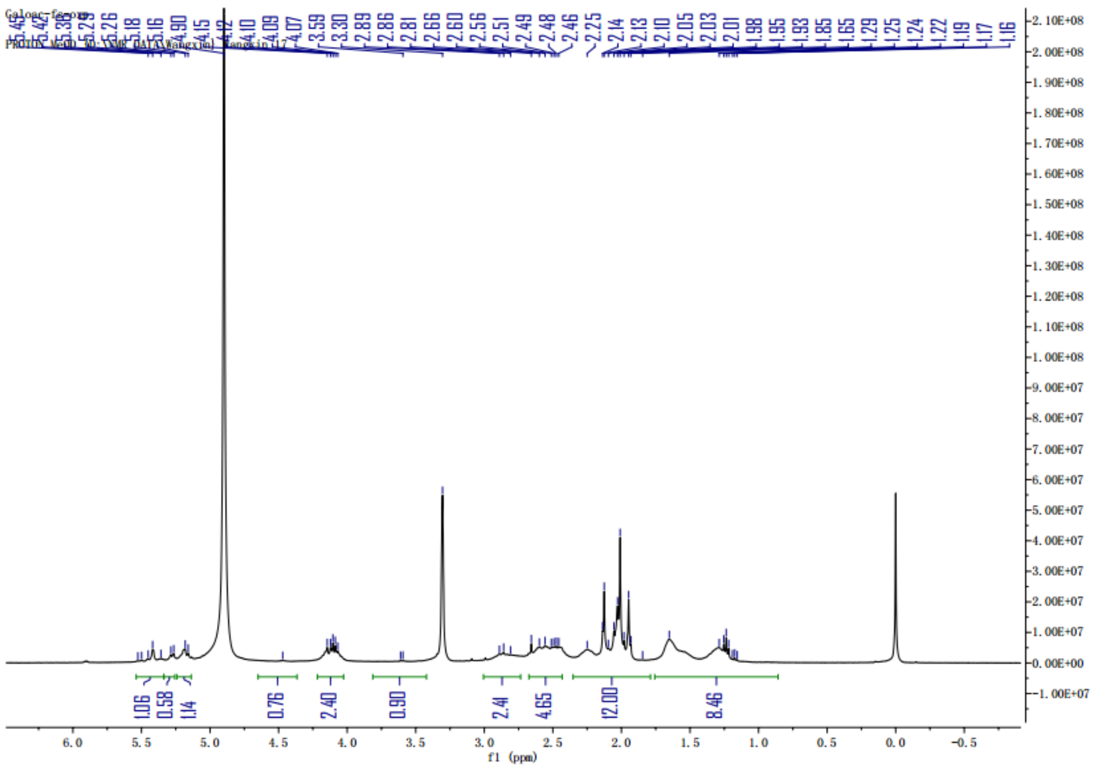

^{13}C NMR spectra for compound 5c

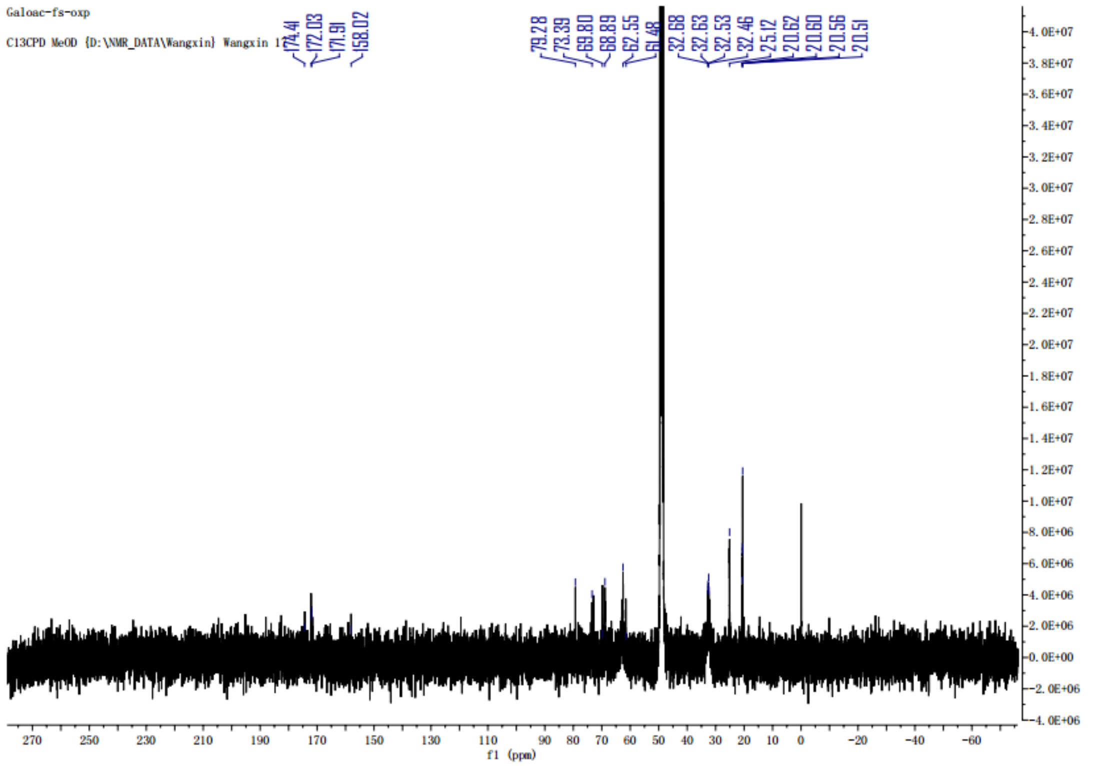

HRMS spectrum for compound 5c

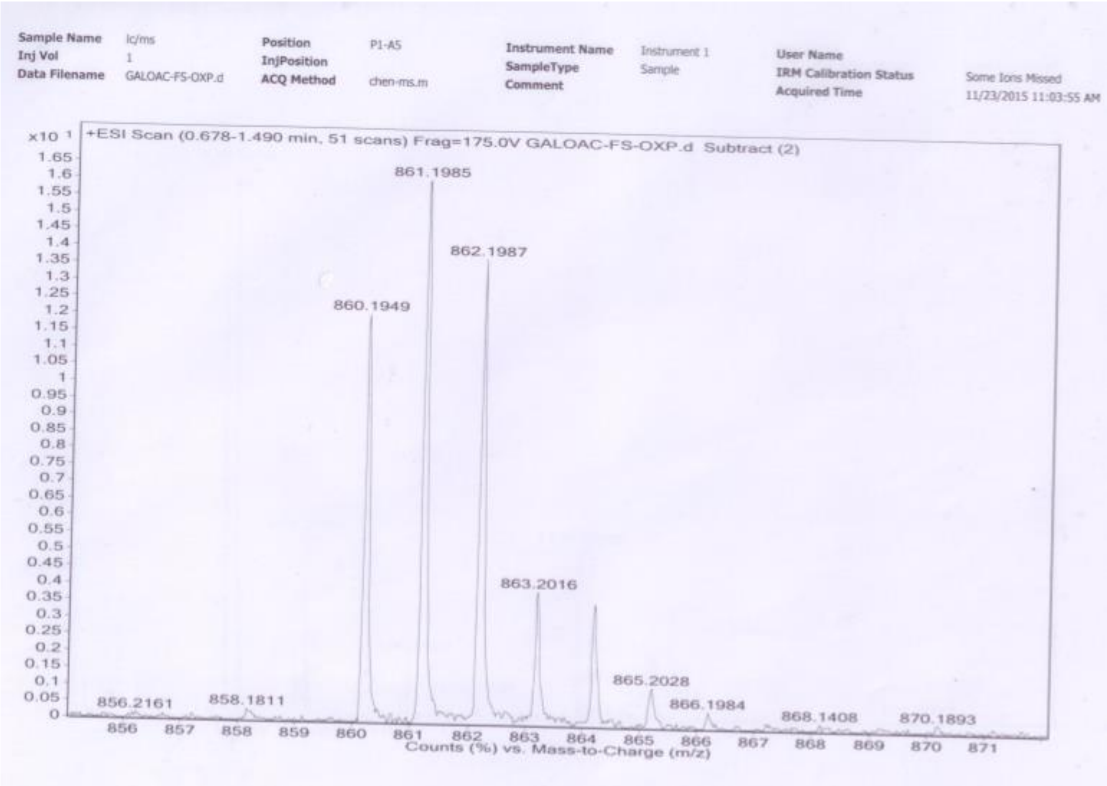

^{1}H-NMR spectrum for compound 6c

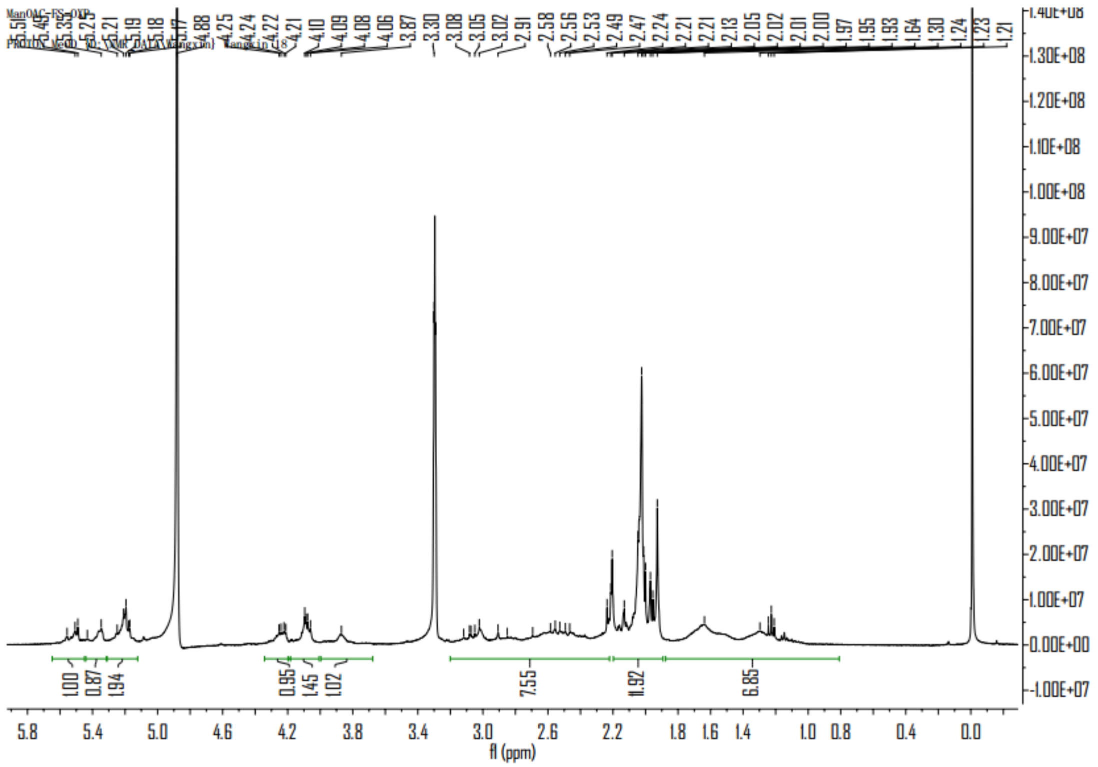

^{13}C NMR spectra for compound 6c

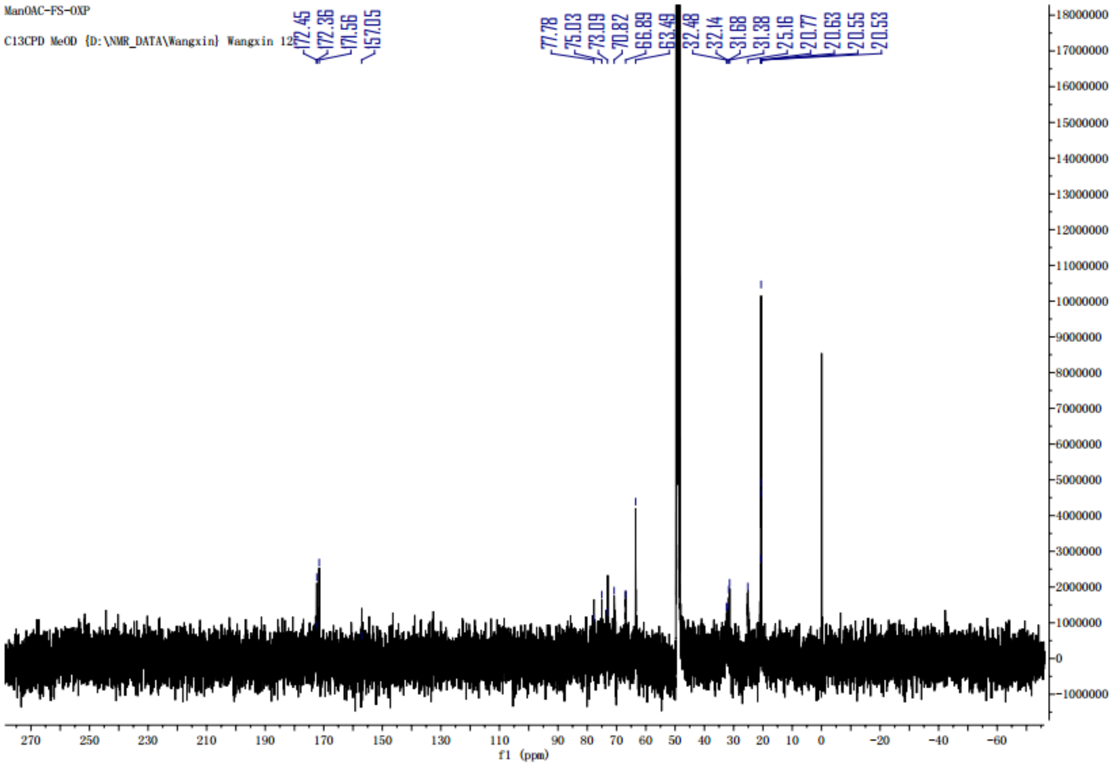

HRMS spectrum for compound 6c

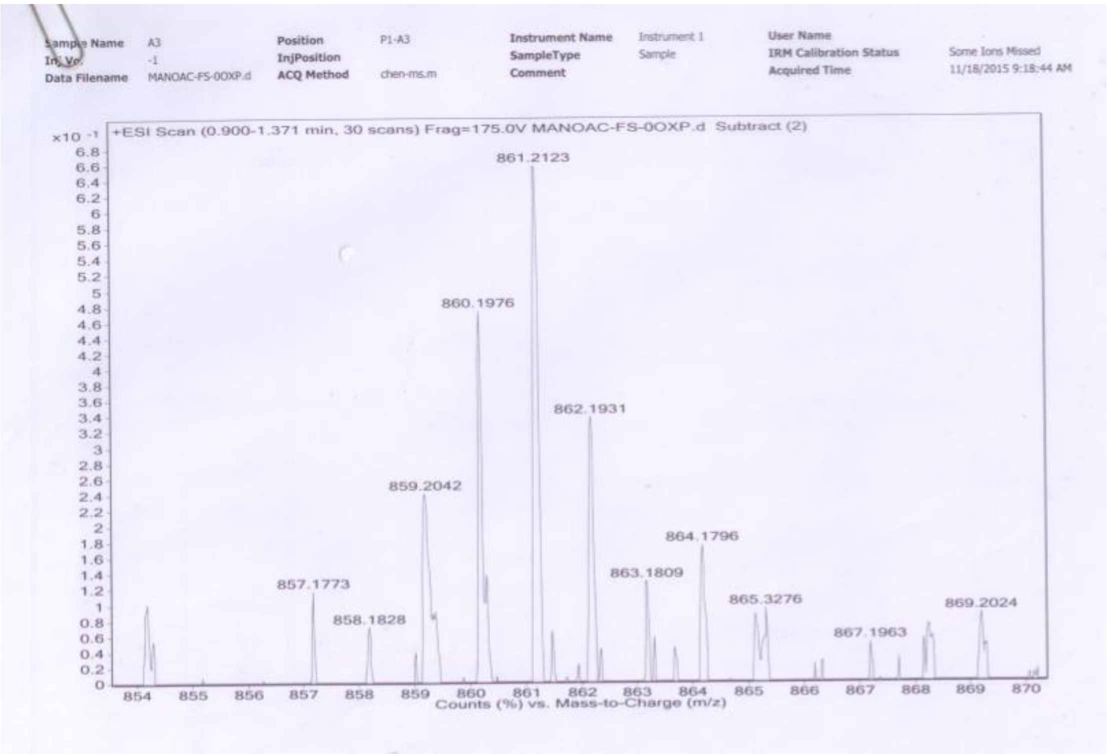

^{1}H-NMR spectrum for compound 1d

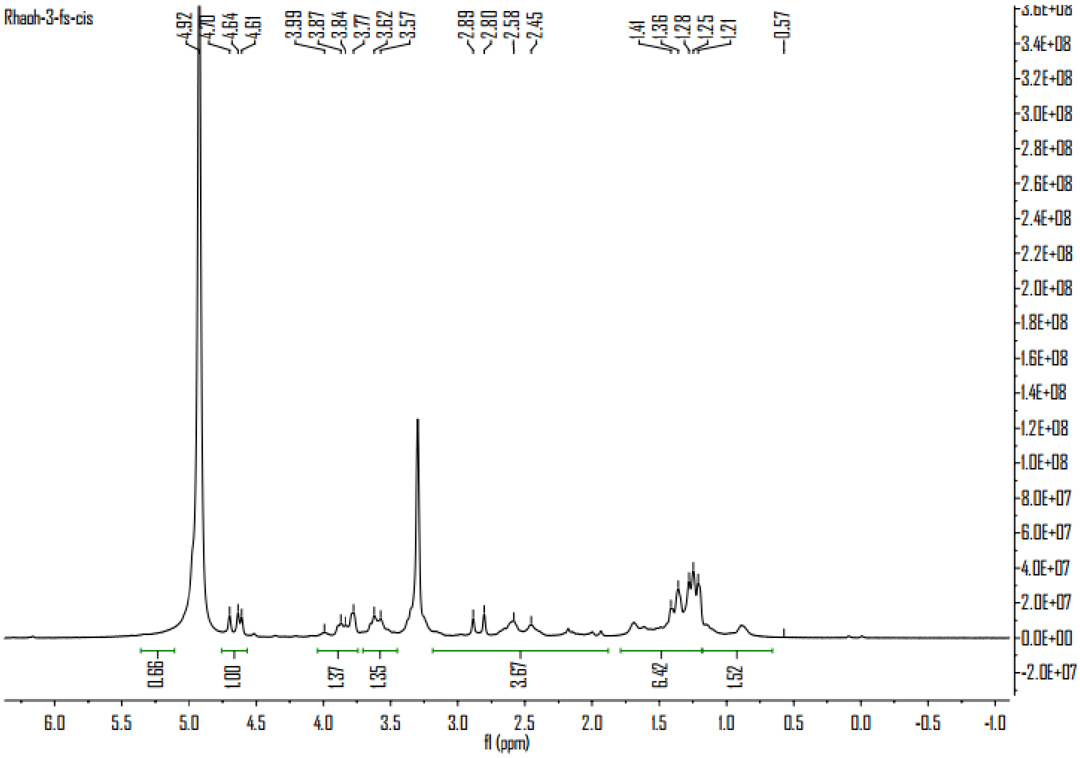

^{13}C NMR spectra for compound 1d

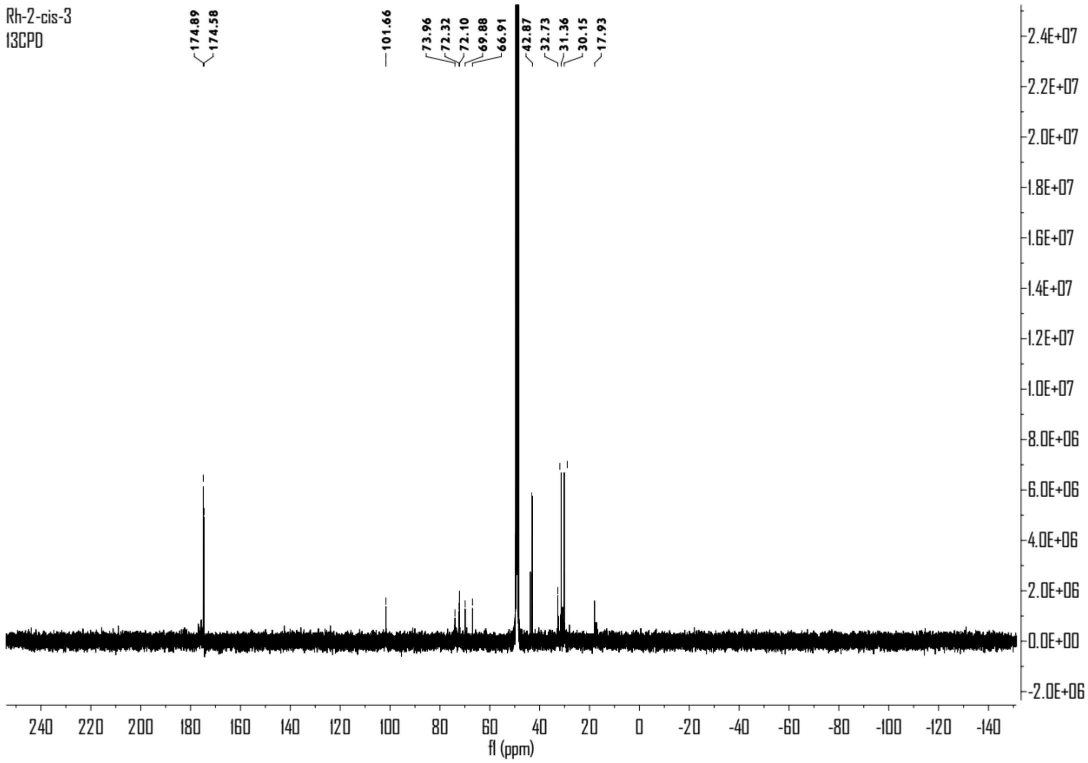

HRMS spectrum for compound 1d

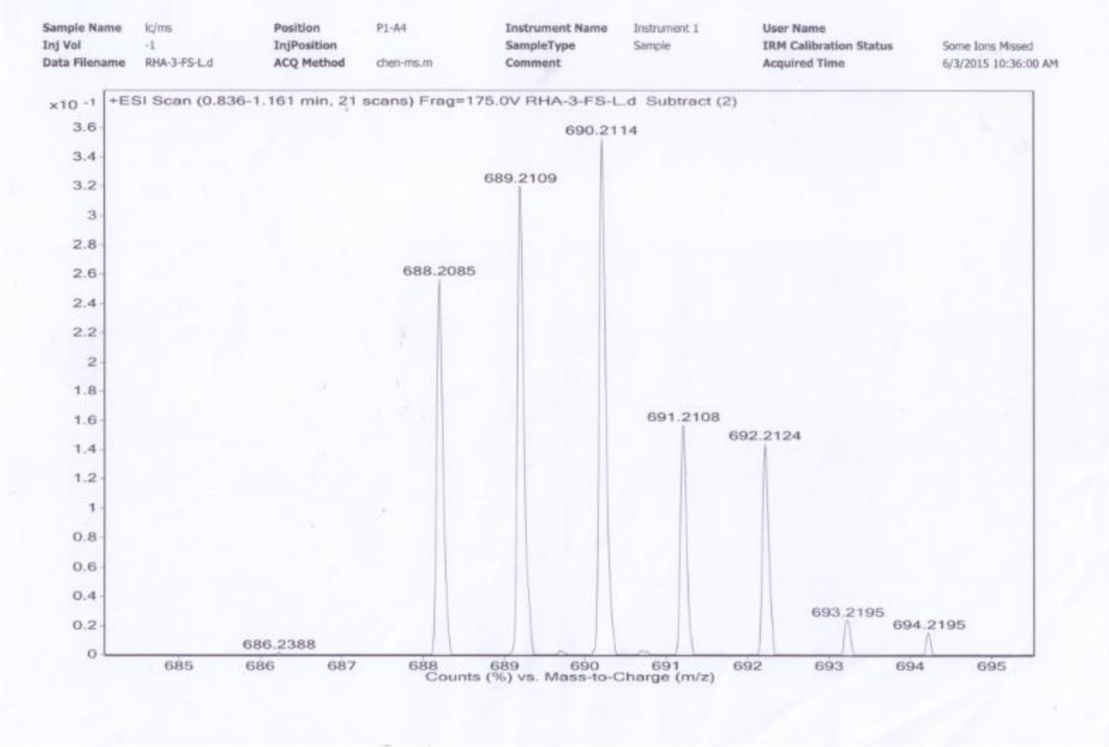

^{1}H-NMR spectrum for compound 2d

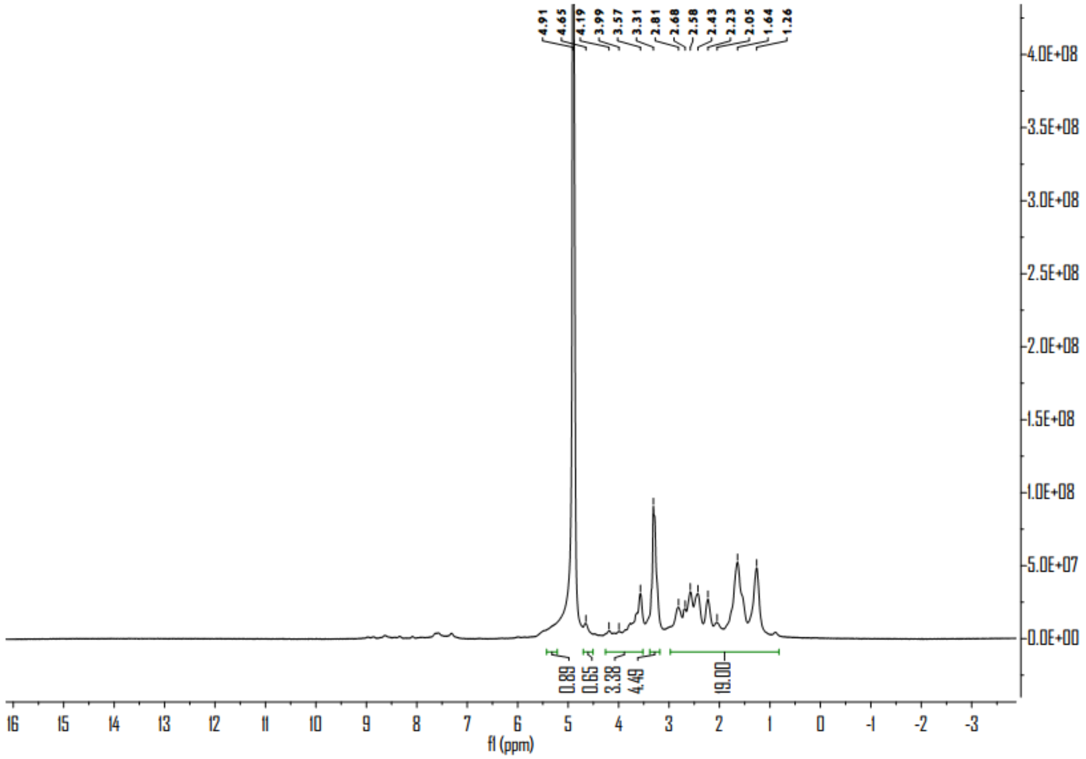

^{13}C NMR spectra for compound 2d

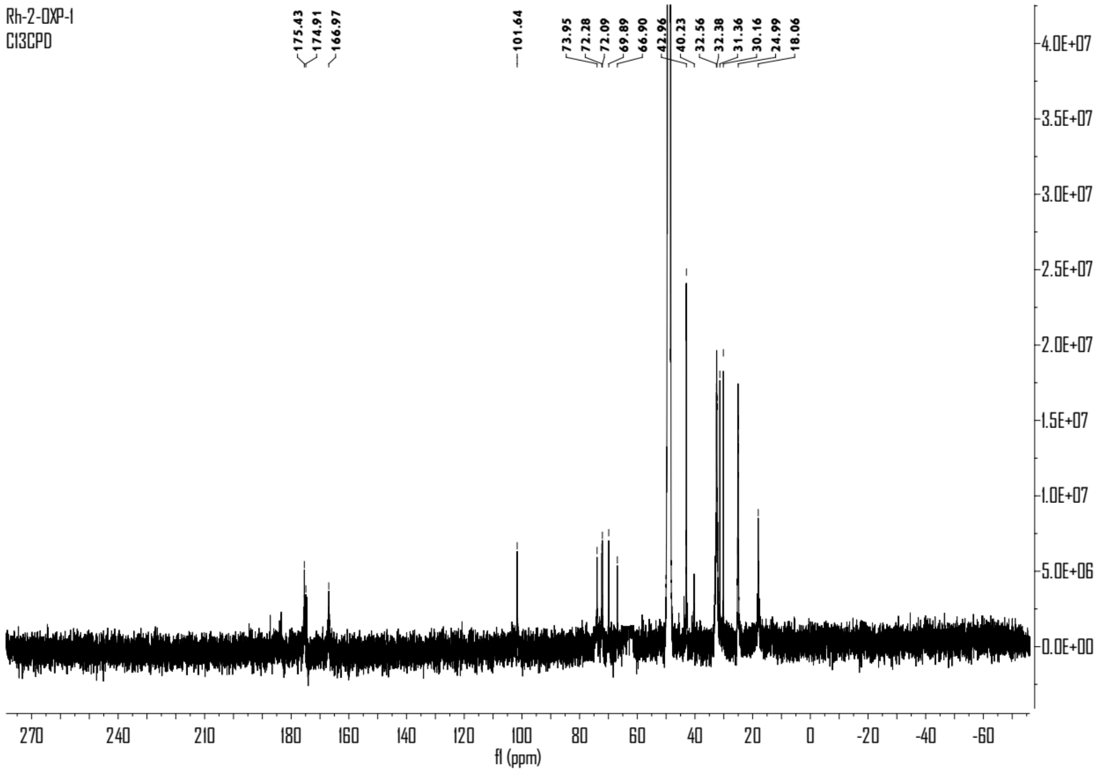

HRMS spectrum for compound 2d

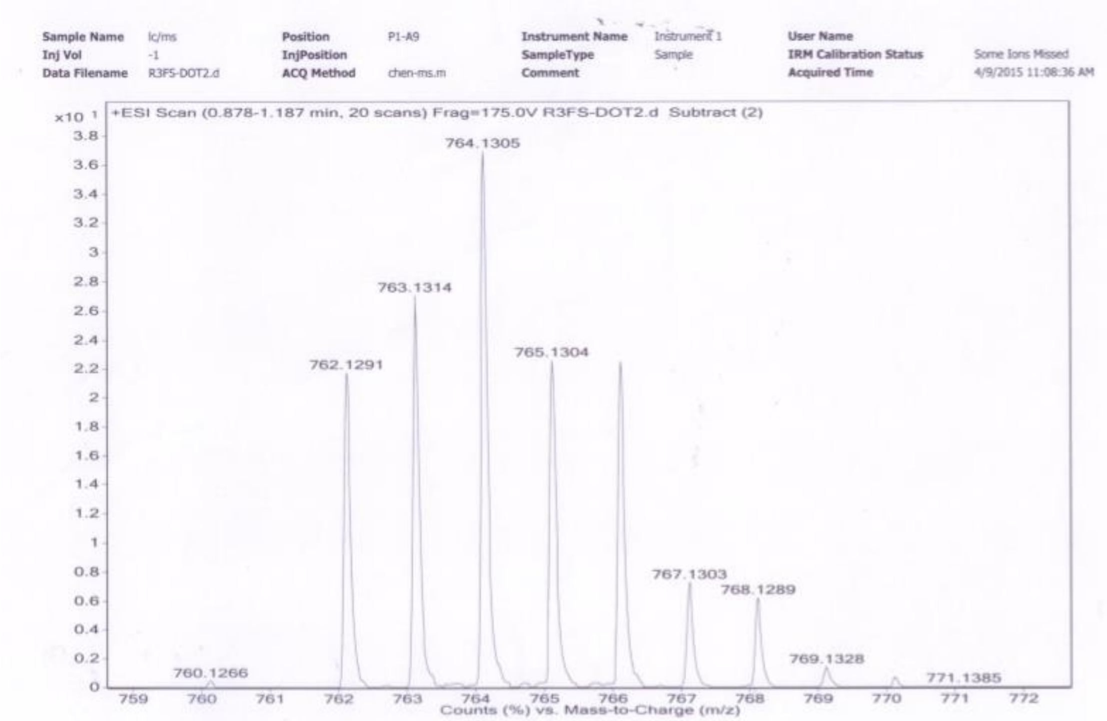

^{1}H-NMR spectrum for compound 3d

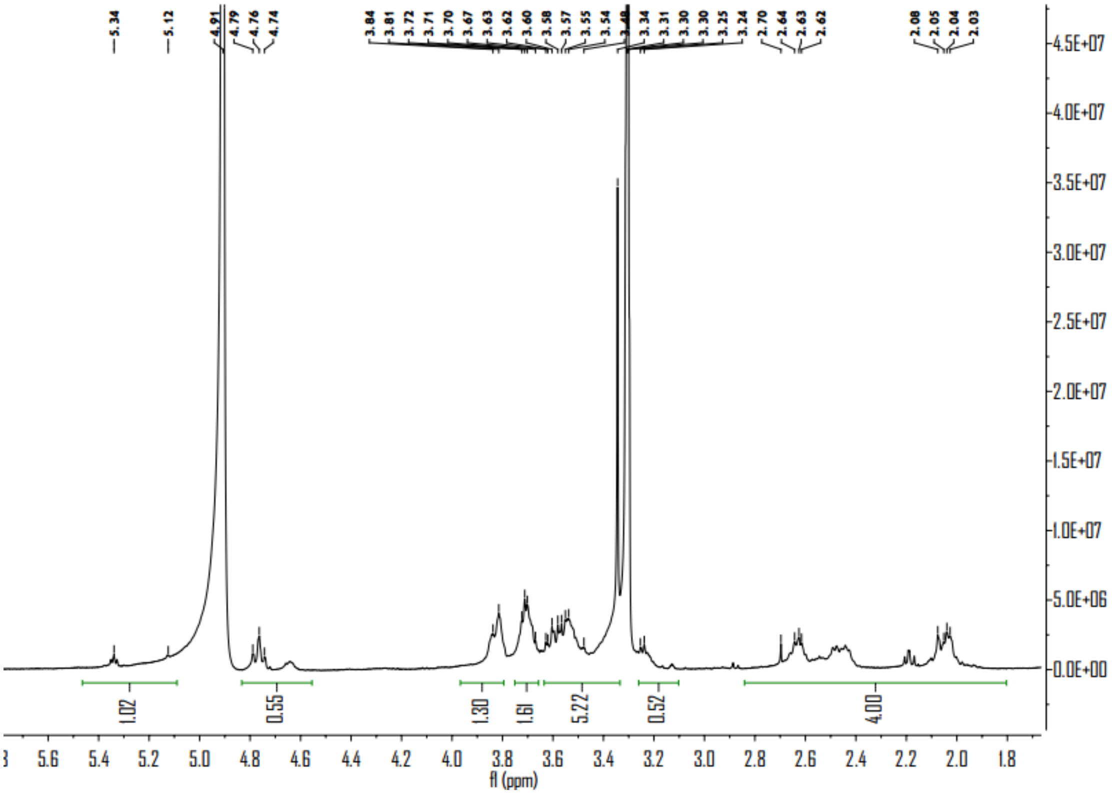

^{13}C NMR spectra for compound 3d

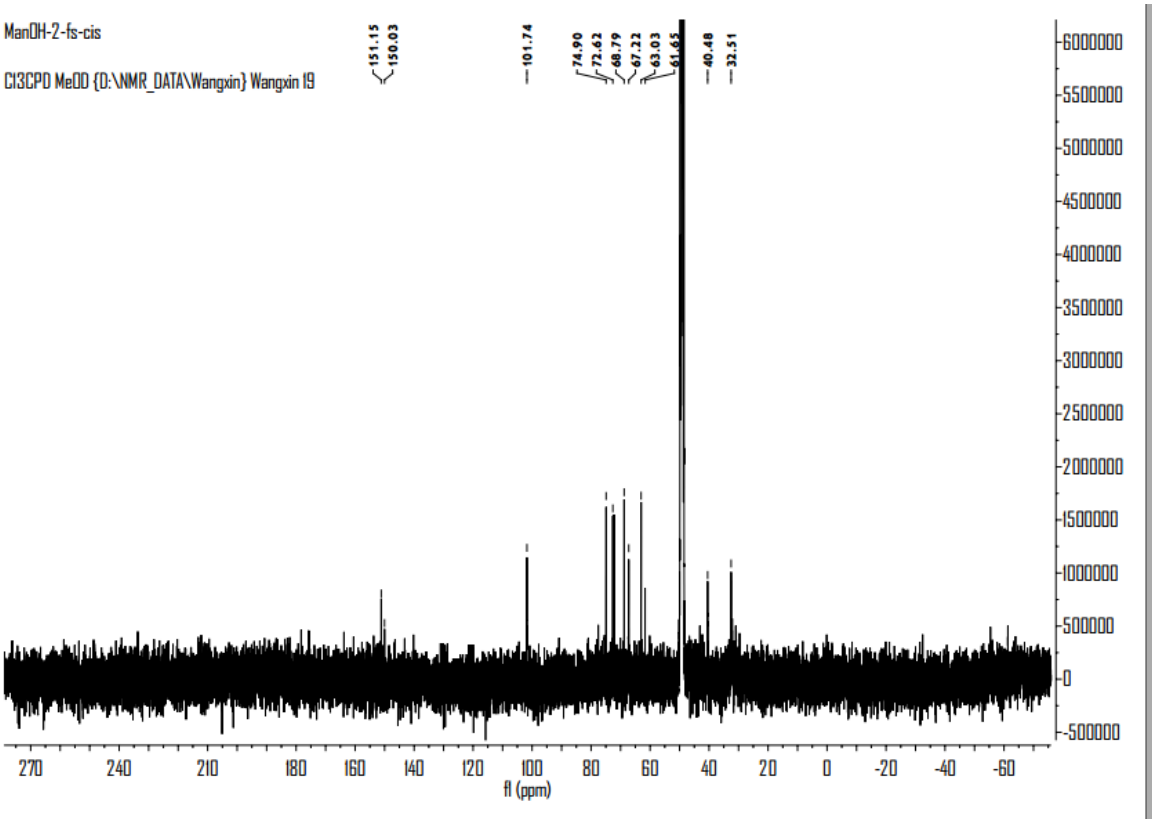

HRMS spectrum for compound 3d

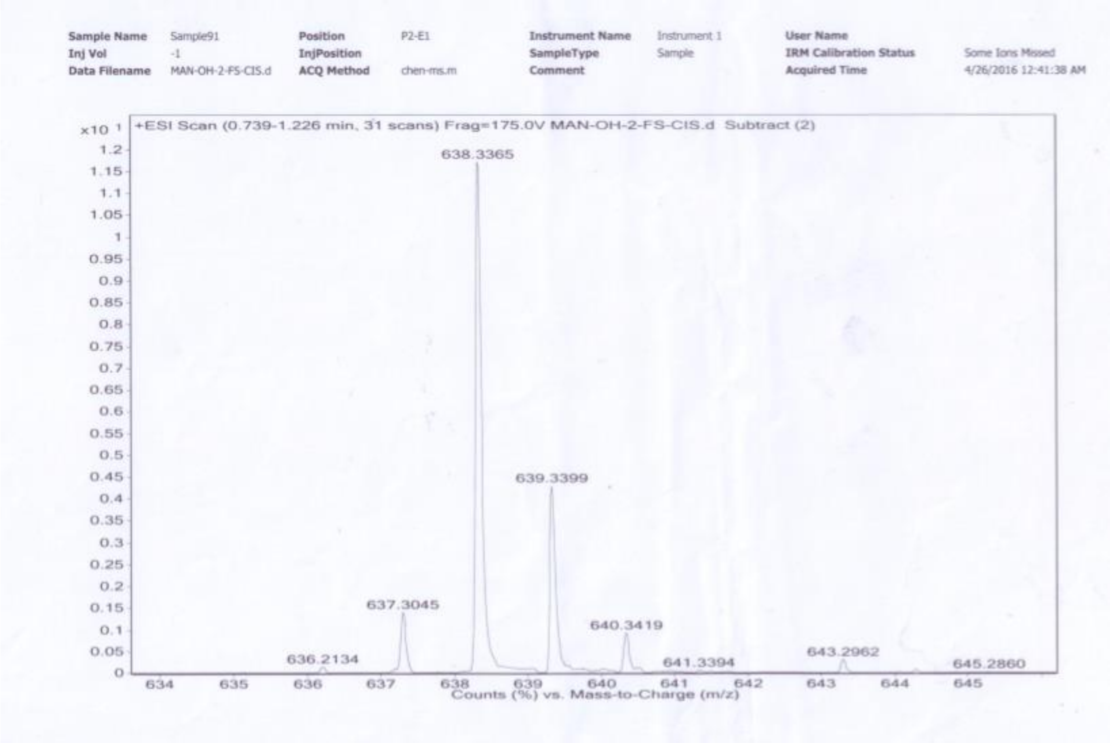

^{1}H-NMR spectrum for compound 4d

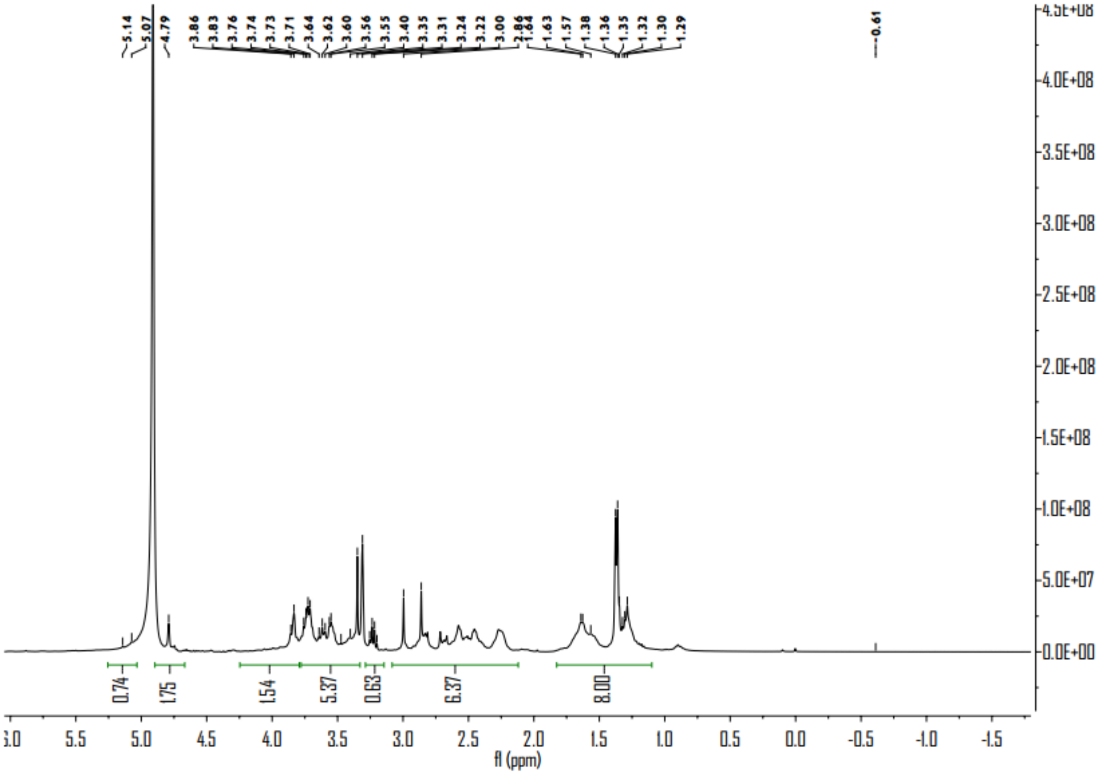

^{13}C NMR spectra for compound 4d

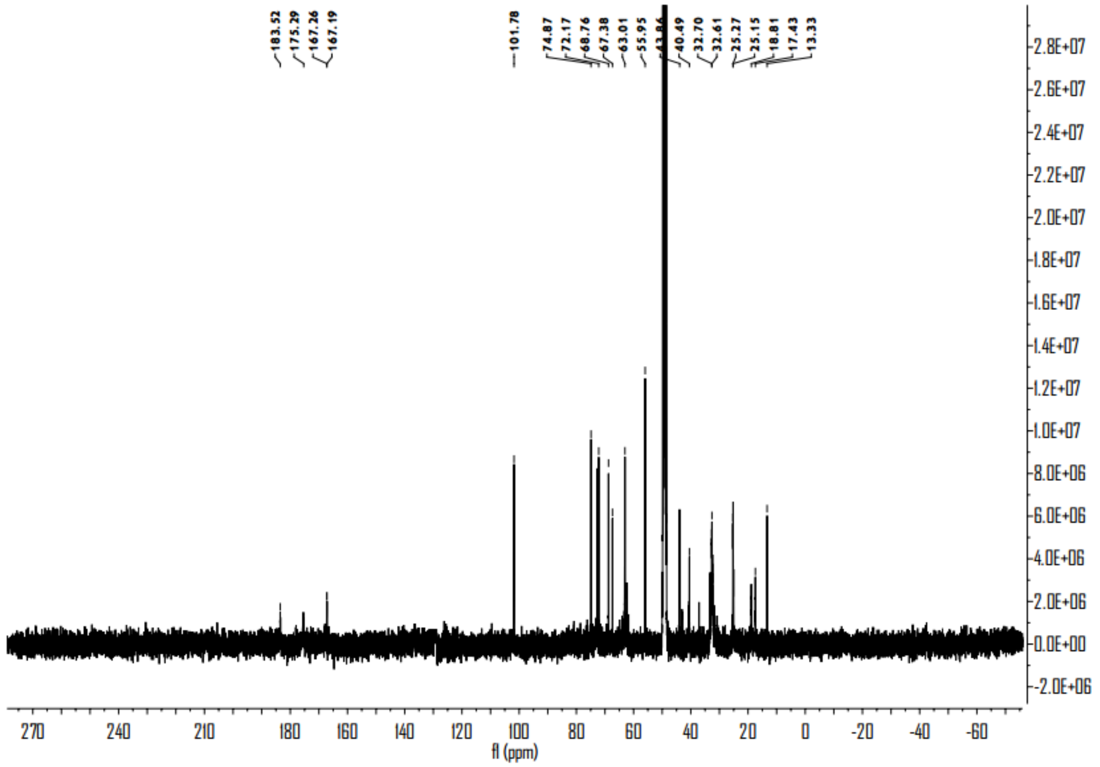

HRMS spectrum for compound 4d

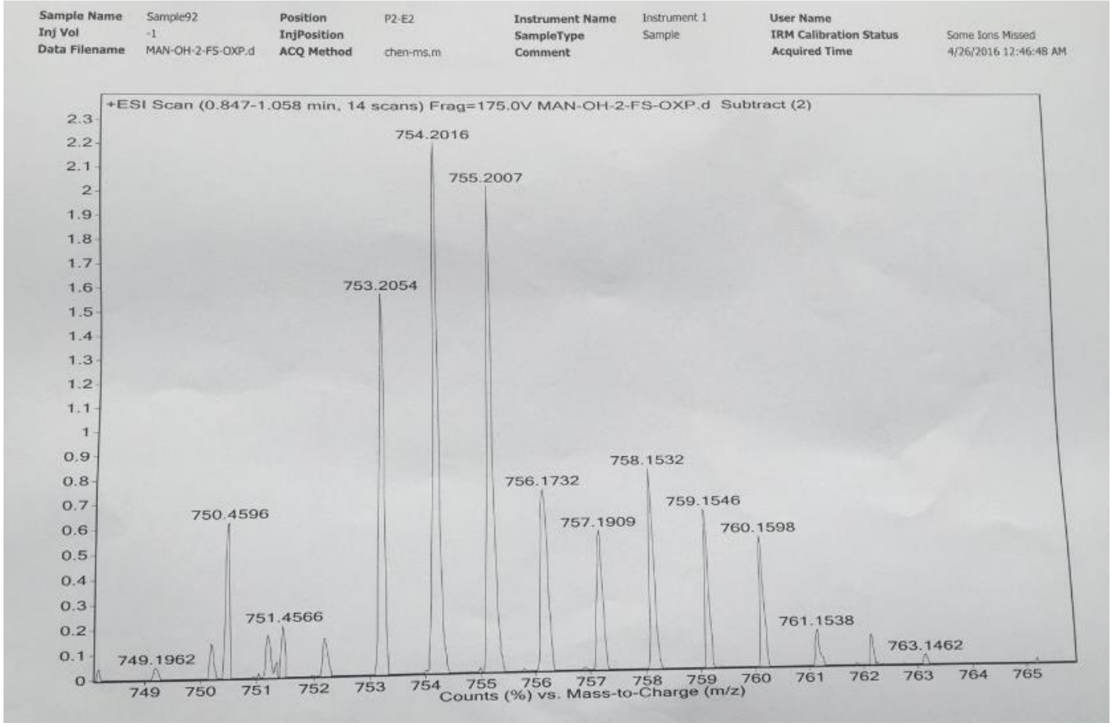

^{1}H-NMR spectrum for compound 5d

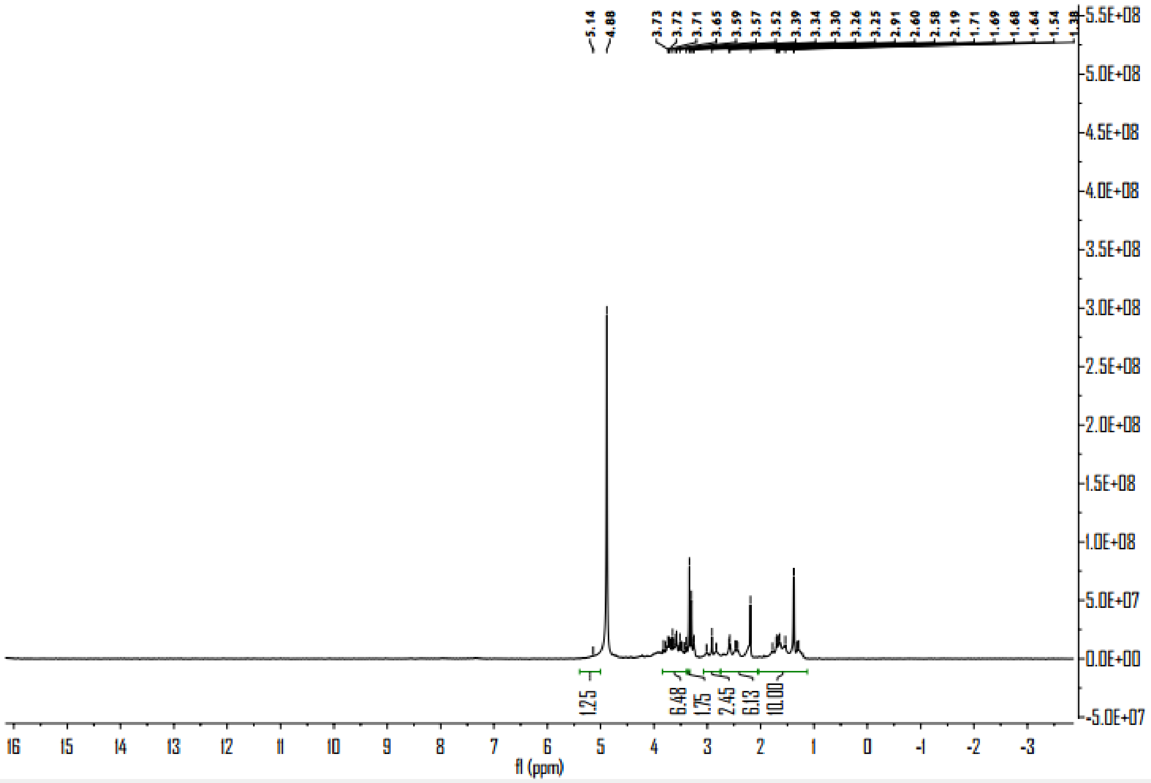

^{13}C NMR spectra for compound 5d

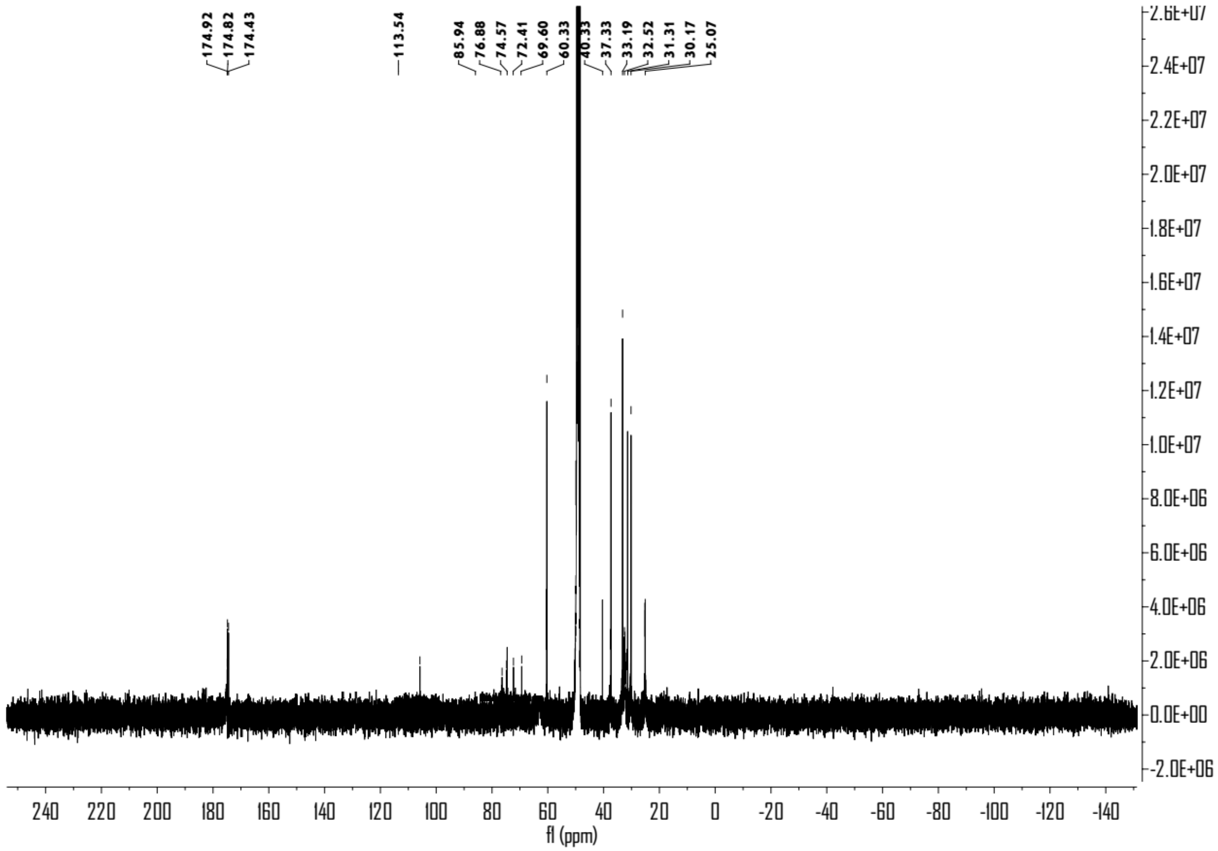

HRMS spectrum for compound 5d

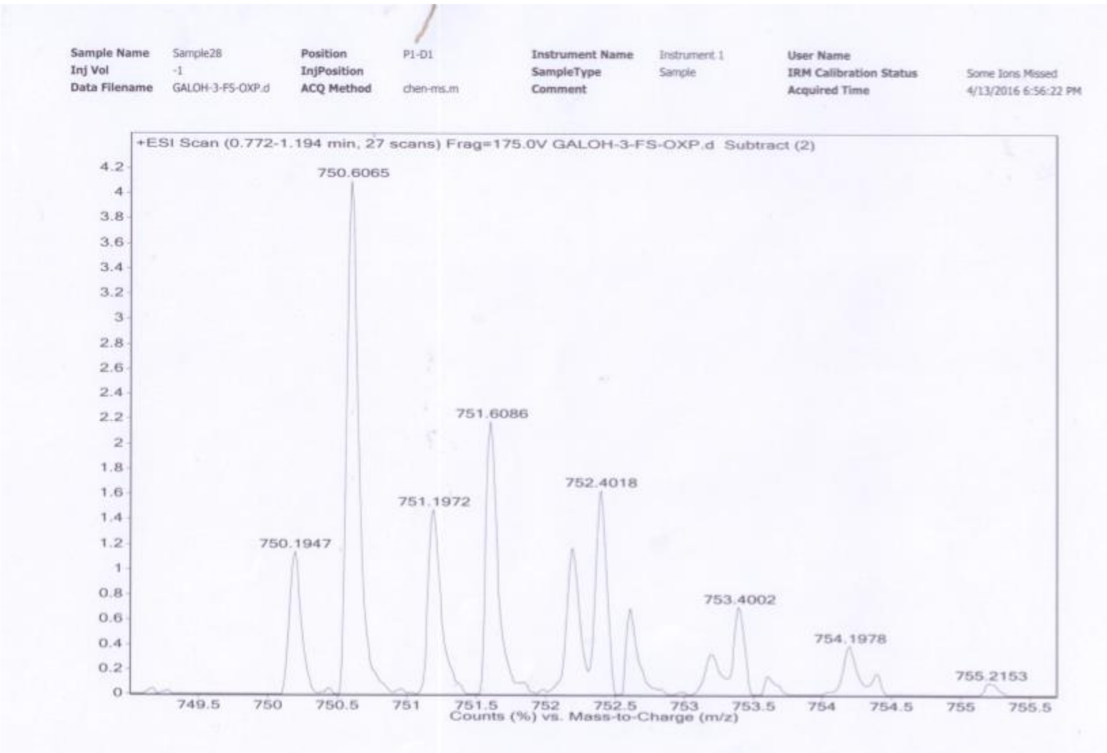

^{1}H-NMR spectrum for compound 6d

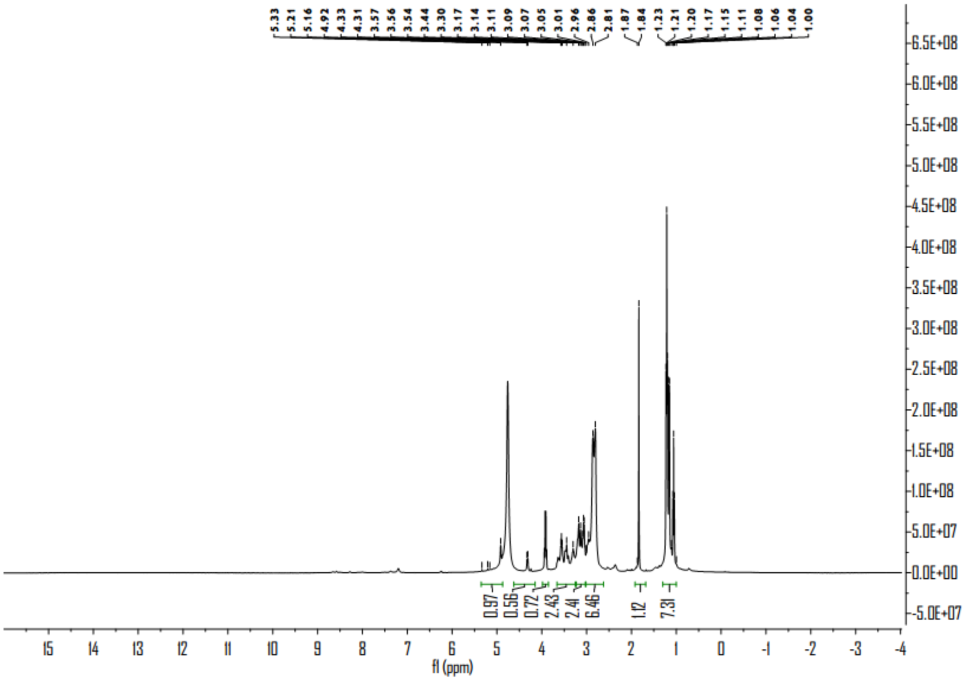

^{13}C NMR spectra for compound 6d

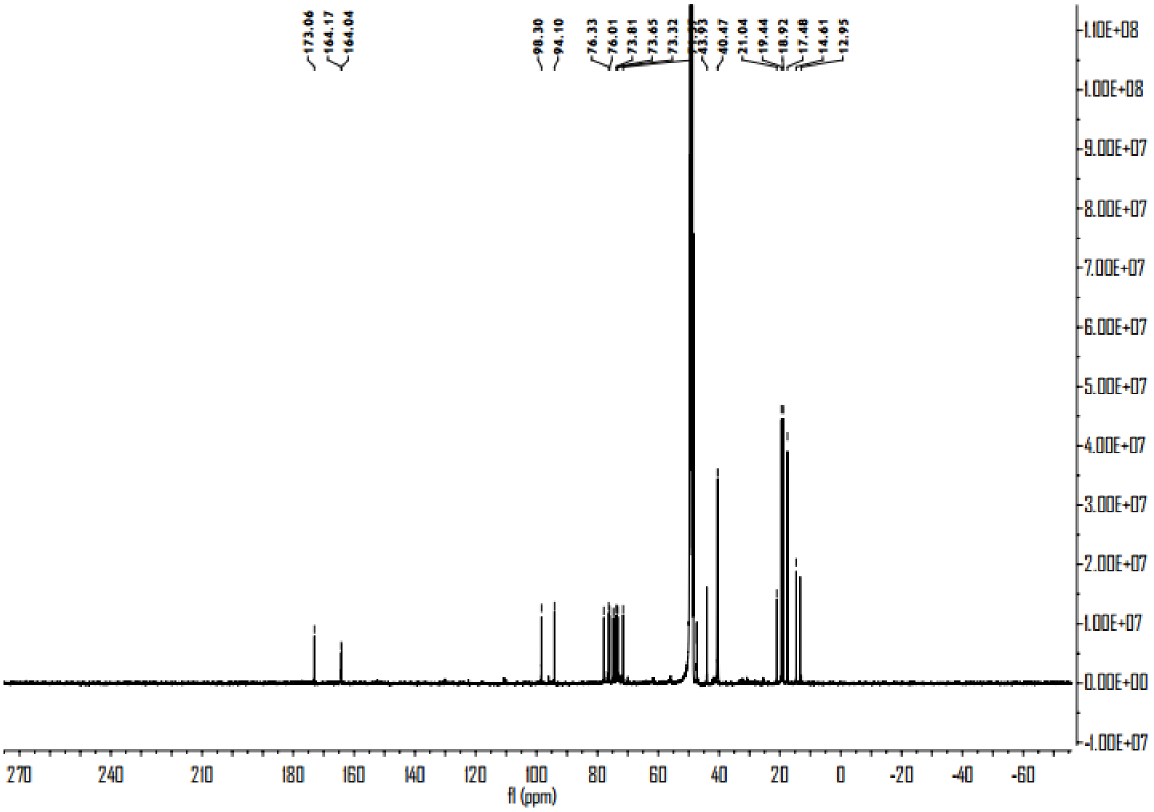

HRMS spectrum for compound 6d

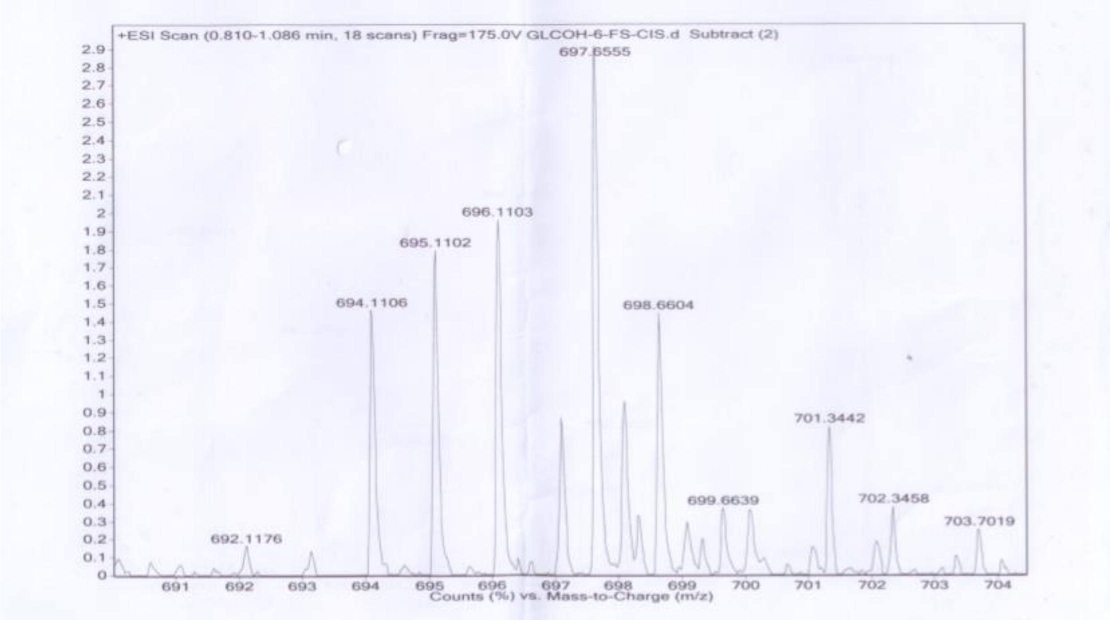

^{1}H-NMR spectrum for compound 7d

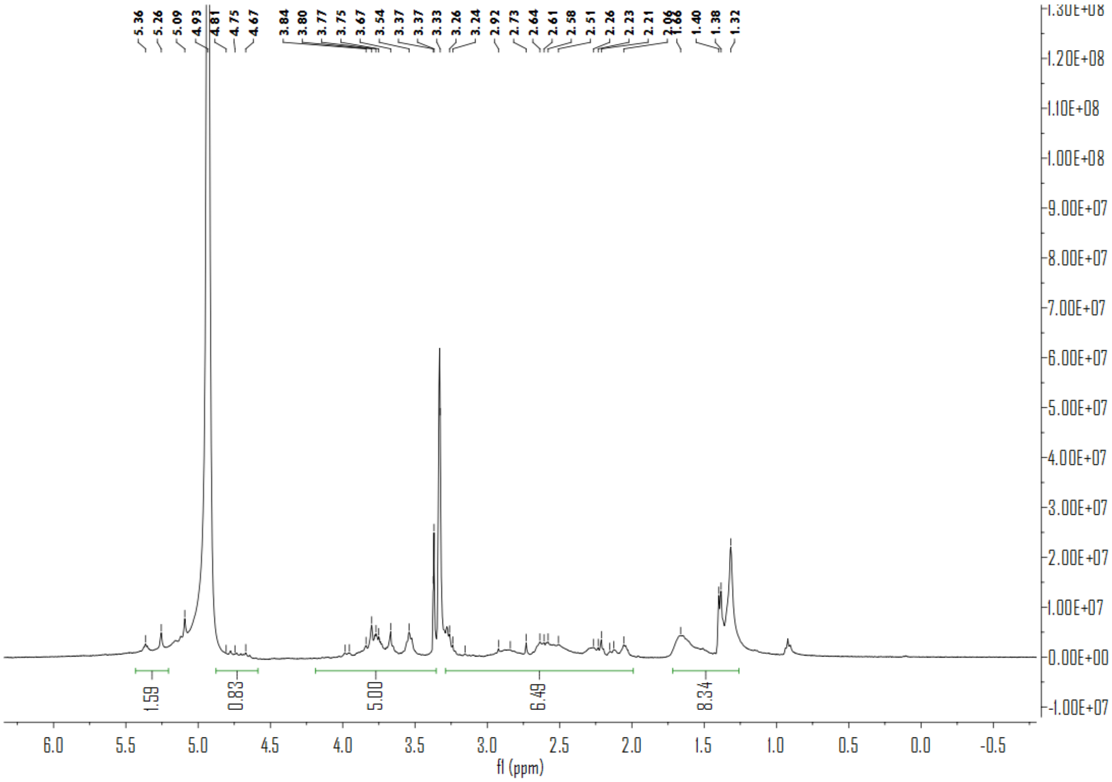

^{13}C NMR spectra for compound 7d

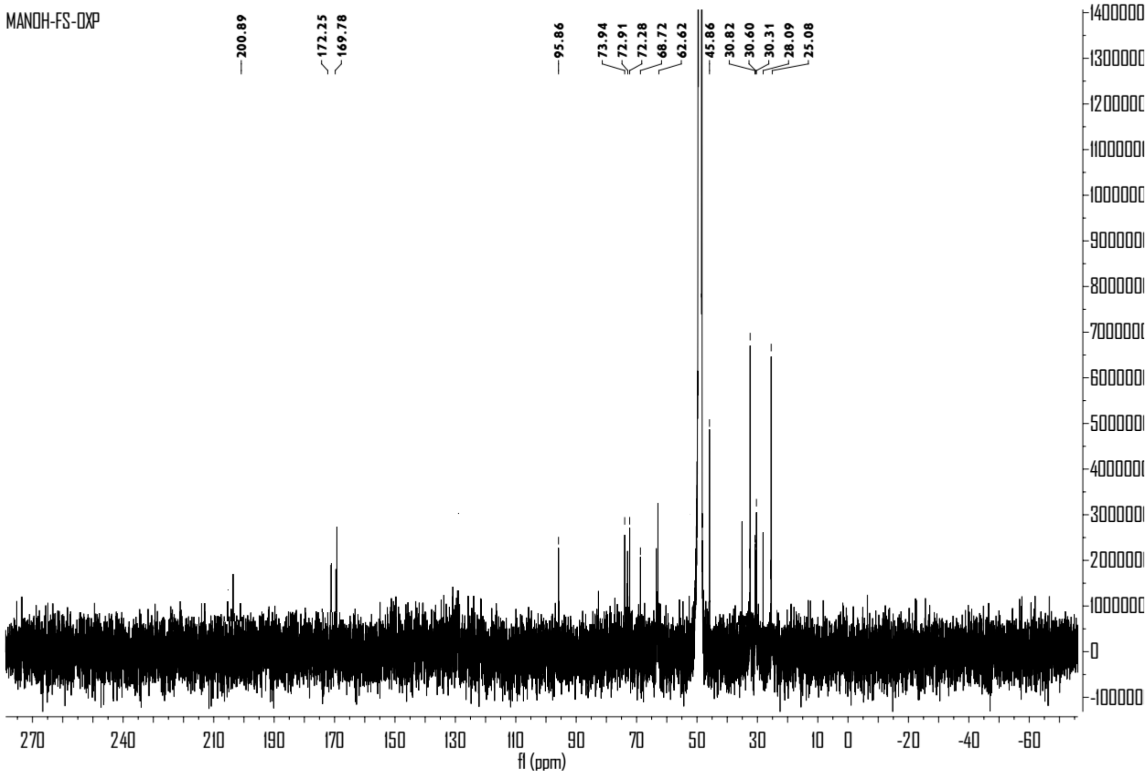

HRMS spectrum for compound 7d

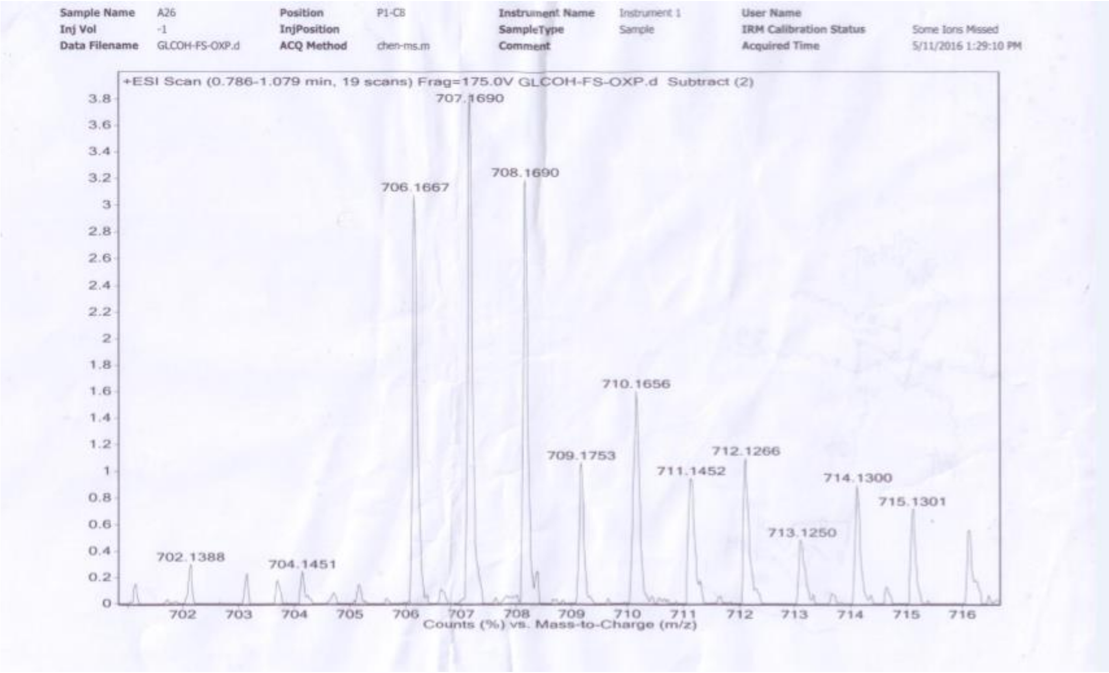

References

[1] F. Neese, Wiley Interdisciplinary Reviews: *Computational Molecular Science* **2012**, *2*, 73-78.

[2] a) A. D. Becke, *Phys Rev A* **1988**, *38*, 3098-3100; b) J. P. Perdew, *Phys Rev B* **1986**, *33*, 8822-8824.

[3] F. Neese, *J Comput Chem* **2003,** *24*, 1740-1747.

[4] F. Weigend, R. Ahlrichs, *Physical Chemistry Chemical Physics* **2005**, *7*, 3297-3305.

[5] D. A. Pantazis, X.-Y. Chen, C. R. Landis, F. Neese, *Journal of Chemical Theory and Computation*, **2008**, *4*, 908-919.

[6] F. Weigend, *Physical Chemistry Chemical Physics* **2006**, *8*, 1057-1065.

[7] M. F. Sanner, *J Mol Graph Model* **1999**, *17*, 57-61.

[8] L. Sun, X. Zeng, C. Yan, X. Sun, X. Gong, Y. Rao, N. Yan, *Nature* **2012**, *490*, 361-366.

[9] O. Trott, A. J. Olson, *J Comput Chem* **2010**, *31*, 455-461.

[10] B. K. Ho, F. Gruswitz, *Bmc Struct Biol* **2008**, *8*, 49.

[11] Version 1.5.0.4 ed., Schrödinger, LLC.
